# Functional divergence of the two Elongator subcomplexes during neurodevelopment

**DOI:** 10.15252/emmm.202115608

**Published:** 2022-06-13

**Authors:** Monika Gaik, Marija Kojic, Megan R Stegeman, Tülay Öncü‐Öner, Anna Kościelniak, Alun Jones, Ahmed Mohamed, Pak Yan Stefanie Chau, Sazia Sharmin, Andrzej Chramiec‐Głąbik, Paulina Indyka, Michał Rawski, Anna Biela, Dominika Dobosz, Amanda Millar, Vann Chau, Aycan Ünalp, Michael Piper, Mark C Bellingham, Evan E Eichler, Deborah A Nickerson, Handan Güleryüz, Nour El Hana Abbassi, Konrad Jazgar, Melissa J Davis, Saadet Mercimek‐Andrews, Sultan Cingöz, Brandon J Wainwright, Sebastian Glatt

**Affiliations:** ^1^ Malopolska Centre of Biotechnology Jagiellonian University Krakow Poland; ^2^ The University of Queensland Diamantina Institute Faculty of Medicine The University of Queensland Woolloongabba QLD Australia; ^3^ Department of Medical Biology and Genetics Faculty of Medicine Dokuz Eylül University İzmir Turkey; ^4^ Institute for Molecular Bioscience The University of Queensland Brisbane QLD Australia; ^5^ Bioinformatics Division The Walter and Eliza Hall Institute of Medical Research Parkville VIC Australia; ^6^ Colonial Foundation Healthy Ageing Centre The Walter and Eliza Hall Institute of Medical Research Parkville VIC Australia; ^7^ The Department of Medical Biology Faculty of Medicine Dentistry and Health Science The University of Melbourne Parkville VIC Australia; ^8^ School of Biomedical Sciences The University of Queensland Brisbane QLD Australia; ^9^ National Synchrotron Radiation Centre SOLARIS Jagiellonian University Krakow Poland; ^10^ Department of Paediatrics (Neurology) The Hospital for Sick Children and University of Toronto Toronto ON Canada; ^11^ Department of Pediatric Neurology Dr. Behçet Uz Children's Hospital İzmir Turkey; ^12^ Department of Genome Sciences University of Washington School of Medicine Seattle WA USA; ^13^ Howard Hughes Medical Institute University of Washington Seattle WA USA; ^14^ Department of Pediatric Radiology School of Medicine Dokuz Eylül University İzmir Turkey; ^15^ Postgraduate School of Molecular Medicine Medical University of Warsaw Warsaw Poland; ^16^ The Department of Clinical Pathology Faculty of Medicine, Dentistry and Health Science The University of Melbourne Parkville VIC Australia; ^17^ The Hospital for Sick Children Toronto ON Canada; ^18^ Department of Medical Genetics Faculty of Medicine and Dentistry The University of Alberta Edmonton AB Canada

**Keywords:** Elongator complex, Elp4, Elp6, neurodevelopment, tRNA modification, Genetics, Gene Therapy & Genetic Disease, Neuroscience, RNA Biology

## Abstract

The highly conserved Elongator complex is a translational regulator that plays a critical role in neurodevelopment, neurological diseases, and brain tumors. Numerous clinically relevant variants have been reported in the catalytic Elp123 subcomplex, while no missense mutations in the accessory subcomplex Elp456 have been described. Here, we identify *ELP4* and *ELP6* variants in patients with developmental delay, epilepsy, intellectual disability, and motor dysfunction. We determine the structures of human and murine Elp456 subcomplexes and locate the mutated residues. We show that patient‐derived mutations in Elp456 affect the tRNA modification activity of Elongator *in vitro* as well as in human and murine cells. Modeling the pathogenic variants in mice recapitulates the clinical features of the patients and reveals neuropathology that differs from the one caused by previously characterized *Elp123* mutations. Our study demonstrates a direct correlation between *Elp4* and *Elp6* mutations, reduced Elongator activity, and neurological defects. Foremost, our data indicate previously unrecognized differences of the Elp123 and Elp456 subcomplexes for individual tRNA species, in different cell types and in different key steps during the neurodevelopment of higher organisms.

## Graphical abstract

The Elongator complex plays a crucial role in neurodevelopment. Patient‐derived amino acid substitutions in the accessory Elp456 subcomplex were shown to affect different types of neurons than previously known mutations in the catalytic core of the complex.

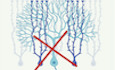

**The paper explained**

**Problem**

Brain development is a fundamental process driven by highly regulated genetic programs. Any disruption of essential neurodevelopmental processes results in neurodevelopmental disorders (NDDs) characterized by an inability to reach cognitive and motor milestones. NDDs affect more than 3% of children worldwide. High co‐occurrence of different NDDs is indicative of shared etiology. Genome‐wide analysis allows identification of associated genetic factors, but without detailed functional studies, the underlying cause and pathophysiology of certain groups of NDDs remain elusive.

**Results**

This study provides the first clinical evidence for missense mutations in the Elongator accessory subcomplex ELP456 to cause neurodevelopmental disorders. Pathogenic variants of *ELP4* and *ELP6* were identified in patients with severe clinical presentation of NDDs. Modeling the patient‐derived mutations in mice recapitulated the complex neurodevelopmental phenotype and revealed neuron‐specific consequences of the mutations. Reconstitution of the Elongator complex *in vitro* showed that the *ELP4*/*6* variants affected its activity by decreasing tRNA modification levels in human and murine cells. Foremost, the obtained data reveal previously unrecognized differences between the ELP123 and ELP456 subcomplexes and their involvement in neuropathology.

**Impact**

The presented work provides a fundamental basis to understand the role of the Elongator complex in the development of neurons and brain structures. The findings highlight novel concepts for studying clinically relevant mutations in mouse models and provide a direct link between mutations in the *ELP4*/*6*, the compromised function of the complex and severe neurodevelopmental phenotypes.

## Introduction

Neurodevelopmental disorders (NDDs) such as global development delay, intellectual disability (ID), and epilepsy are characterized by developmental deficits in cognition, learning, control, and execution of motor skills due to abnormalities in the brain development (Parenti *et al*, 2020). High co‐occurrence of NDDs indicates a shared underlying molecular cause of certain groups of pathologies. Despite major efforts in the field to find the genes driving the various subgroups of disorders, the phenotypic heterogeneity of these conditions impedes the confident identification of common genetic factors (Hormozdiari *et al*, 2015; Chow *et al*, 2019; Carvill *et al*, 2021; Peng *et al*, 2021).

Elongator is a large and highly conserved multi‐subunit macromolecular complex that acts as a global translational regulator by modifying the so‐called “wobble” base (U_34_) in the anticodon of 12 mammalian tRNAs to form 5‐carboxymethyl‐uridine (cm^5^U_34_). Subsequently, cm^5^U_34_ can be converted into either 5‐carbamoylmethyl‐uridine (ncm^5^U_34_), 5‐methoxycarbonylmethyl‐uridine (mcm^5^U_34_), or 5‐methoxycarbonymethyl‐2‐thiouridine (mcm^5^s^2^U_34_) (Schäck *et al*, 2020) by other enzymes. All of these modifications have a strong impact on the kinetics of translation elongation by regulating efficiency and accuracy of codon‐anticodon pairing in the A‐site of ribosomes (Nedialkova & Leidel, 2015). Elongator consists of two copies of each of the six subunits (Elp1‐6) and forms two discrete subcomplexes, namely Elp123 and Elp456 (Dauden *et al*, 2017). In yeast, each of the six subunits is equally important for the integrity and function of the fully assembled Elongator complex. The Elp123 subcomplex acts as a scaffold for the Elp3 subunit, which catalyzes the actual RNA base modification reaction (Dauden *et al*, 2019; Lin *et al*, 2019). The hetero‐hexameric Elp456 subcomplex is attached asymmetrically to Elp123 (Dauden *et al*, 2017) and forms a ring‐like structure that possesses adenosine triphosphatase hydrolysis (ATPase) activity and regulates tRNA binding (Glatt *et al*, 2012; Dauden *et al*, 2019).

A growing body of evidence indicates that the Elongator complex is one of the master regulators of neurodevelopment in higher eukaryotes (Kojic & Wainwright, 2016; Hawer *et al*, 2018). Pathogenic variants in Elongator subunits have been identified in a number of cases with severe and complex NDDs, including *ELP1* pathogenic variants in familial dysautonomia (FD) (Anderson *et al*, 2001; Slaugenhaupt *et al*, 2001), *ELP3* variants associated with amyotrophic lateral sclerosis (ALS) (Simpson *et al*, 2009; Kwee *et al*, 2012), and pathogenic variants in *ELP2* found in patients with global developmental delay, ID, autism, and epilepsy (Strug *et al*, 2009; Najmabadi *et al*, 2011; Reinthaler *et al*, 2014; Addis *et al*, 2015; Cohen *et al*, 2015; Alizadeh *et al*, 2018; Toral‐Lopez *et al*, 2020; Kojic *et al*, 2021). In addition, the *ELP4*‐*PAX6* locus has been linked to Rolandic epilepsy (Panjwani *et al*, 2016; Duan *et al*, 2021), which was questioned by others (Reinthaler *et al*, 2014). As the genomic alterations seem to affect the non‐coding and intronic regions, the ELP4 protein remains most likely unaffected, and the misregulation of PAX6 seems more likely to cause the condition (Panjwani *et al*, 2016). Decreased levels of mcm^5^s^2^U_34_ have been found in FD (Karlsborn *et al*, 2014) and ALS (Bento‐Abreu *et al*, 2018) patients, indicating that impairment of tRNA modification could directly drive the underlying diseases. Our current understanding of the role of the Elongator complex in guiding brain development and disease is largely limited to results from conditional loss‐of‐function (LOF) mouse models and primarily mutations in the catalytic Elp123 subcomplex (Laguesse *et al*, 2015; Chaverra *et al*, 2017; Goffena *et al*, 2018). Recent efforts have been made to fully define the underlying neuropathology and the molecular mechanism behind *ELP2* pathogenic variants in patients with NDDs (Kojic *et al*, 2021). Although we have previously investigated role of the *Elp6* gene in neurological disorders in mice (Kojic *et al*, 2018), no clinically relevant patient‐derived pathogenic variants in the accessory Elp456 subcomplex have been modeled and studied *in vitro* nor *in vivo*, yet. Thus, it remains unclear whether this subcomplex plays a role for the central nervous system (CNS) development and associated pathologies.

In this work, we provide the clinical and molecular evidence for the direct involvement of ELP456 in NDDs by identifying a patient with *ELP4* and two patients with *ELP6* variants presenting with a range of severe neurodevelopmental defects. We demonstrate that these variants decrease the stability of the subcomplex, impair the activity of the Elongator complex, and are sufficient to recapitulate the clinical phenotype in mice. Foremost, our comprehensive study of the clinically relevant Elongator mutations reveals yet unrecognized differences of the Elongator subcomplexes in epitranscriptomic processes in neuronal subtypes, both leading to profound but distinguishable CNS defects in model systems.

## Results

### *ELP4*/*6* pathogenic variants cause a complex neurodevelopmental phenotype

**Figure 1:**
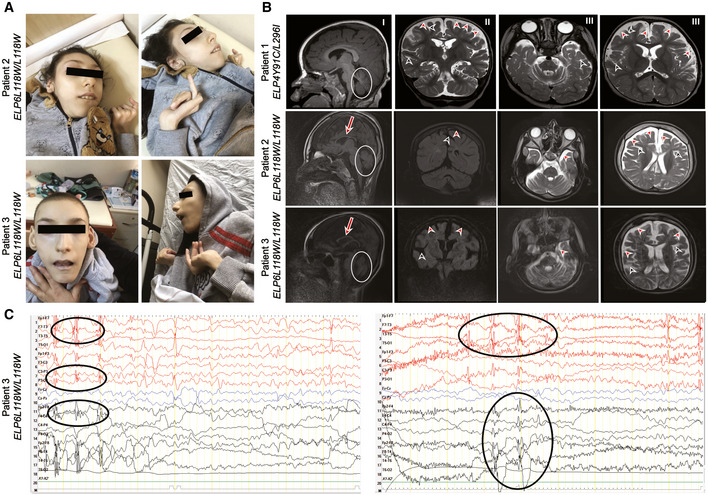
Clinical information of the patients with neurodevelopmental disorders and identified *ELP4*/*6* variants Anterior and lateral photographs of two patients with *ELP6* variants showing dysmorphic features at 20 (Patient 2) and 19 (Patient 3) years of age.Mid‐sagittal (I), coronal (II) and transversal (III) MRI scans of Patient 1 with newly identified *ELP4* variants and Patient 2 and Patient 3 with *ELP6* variants. Atrophy of the cerebellum with prominence of the cerebellar folia is marked by circles. Arrows indicate thin corpus callosum, red arrowheads point to enlarged extra‐axial CSF spaces, and black arrowheads to cerebral sulci in keeping with cerebral and cerebellar volume loss.EEG of Patient 3. Left panel: 1–2 Hz left fronto‐temporal spike and multi‐spike slow wave discharges (Fp1‐F7 and T3‐T5) spread to the homologous same hemisphere and opposite hemisphere, predominantly seen on bipolar montage EEG. Right panel: Spike‐ and multi‐spike slow wave activities together with fast rhythms originating from bilateral fronto‐temporal‐frontocentral regions indicate electrographic seizure activity. Described changes are marked by circles.

**Figure EV1:**
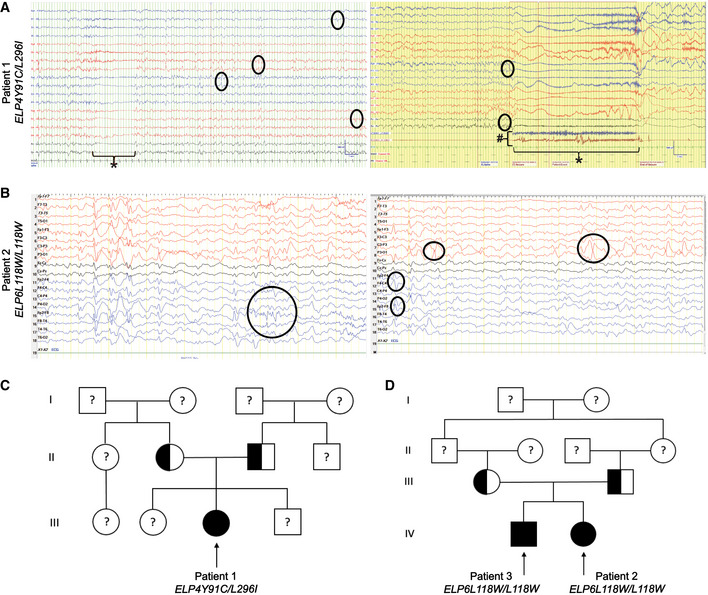
Representative EEG recordings and pedigree trees of the patients with identified *ELP4* and *ELP6* variants Bipolar EEG montage for Patient 1. Left panel: Frequent interictal epileptiform discharges (few examples circled) are seen in the bilateral hemispheres. These occur in non‐evolving runs lasting up to bilateral posterior head regions for seconds. Occasional to intermittent, low‐amplitude, attenuated cerebral activity of electrodecremental episodes (*) without clinical signs are captured. Right panel: A typical electroclinical seizure (*) consisting of arm and leg extension, is captured during the recording. One of the legs may be shaking, possibly due to vibration tonic posturing. Low to medium‐amplitude polyspikes (examples circled) over the bilateral frontal central region followed by high‐amplitude slow waves and low‐amplitude fast waves with muscle artifact (#). The initial polyspikes varied in length and locations. The seizures vary from 6 to 47 s.EEG of Patient 2. Left panel: Bilateral centroparietal‐parietooccipital multispike‐wave activities and bilateral frontocentral‐frontotemporal sharp‐slow wave discharges occur asynchronously. Right panel: Centroparietal‐parietooccipital (indicated on left) and frontocentral‐frontotemporal (indicated on right) sharp and slow multifocal wave activities occur by forming phase encounters. Described changes are marked by circles.Pedigree map of the patient with *ELP4Y91C*/*L296I* variants. Squares indicate male and circles female family members. Solid symbols mark the affected patient (arrowhead; Patient 1), half‐filled symbols the variant carriers and question marks unknown genotypes.Pedigree map of the patients with *Elp6L118W* variants. Squares indicate male and circles female family members. Solid symbols mark the affected patients (arrowheads; Patients 2 and 3), half‐filled symbols the variant carriers and question marks unknown genotypes.

During routine clinical examination, we identified one individual with two missense variants in *ELP4* and two individuals with a missense variant in *ELP6* presenting with a global developmental delay and dysmorphic features (Table 1 and Fig 1A). Other prominent clinical features consisted of ID, seizures, and motor dysfunction. Further examination revealed muscle spasm, hypotonia, lack of language, feeding difficulties, and inability to walk. Brain magnetic resonance imaging (MRI) showed microcephaly with cerebral and cerebellar atrophy and a thin corpus callosum indicating myelination defects or axon deficits (Table 1 and Fig 1B). Electroencephalography (EEG) detected abnormal background activity and generalized seizures (Table 1 and Figs 1C and, EV1A and B). *ELP4* and *ELP6* variants were identified by whole‐exome sequencing. The affected individuals harbored either compound heterozygous missense variants in *ELP4*, *ELP4Y91C*/*L296I*, or homozygous missense variant in *ELP6*, *ELP6L118W*/*L118W*. Healthy parents were heterozygous carriers for the identified variants (Fig EV1C and D). Subsequently, we set out to establish a complete biochemical characterization of the murine and human Elp456 subcomplexes to further understand the direct consequences of the variants on Elongator complex function.

**Table 1:** Clinical features of patients carrying disease‐causing *ELP4* and *ELP6* variants.

	Patient 1	Patient 2	Patient 3
Elongator variants	ELP4 p.L296I + p.Y91C	ELP6 p.L118W	ELP6 p.L118W
Sex, age	Female, 8 y, 9 m	Female, 20 y	Male, 19 y
Prenatal/neonatal course	Uncomplicated/hypotonia in the neonatal period	Uncomplicated	Uncomplicated
Developmental delay	Severe	Severe	Severe
Intellectual disability	Severe	Severe	Severe
Age of sitting/walking	Never achieved	Never achieved	Never achieved
Language abilities	Non‐verbal	Non‐verbal	Non‐verbal
Use of hands	No purposeful movements	No purposeful movements	No purposeful movements
Behavior/ASD	Cries for needs	No evidence available	No evidence available
Feeding difficulty	Yes	Yes	Yes
Neurological exam	Generalized hypotonia, increased muscle tone in right arm, decreased reflexes in lower extremities; reflexes 3+ UE and 4+ knee jerk with clonus and ankle jerk with 4+ reflexes and clonus	Spastic tetraparesis, hyperactive deep tendon reflexes	Spastic tetraparesis, hyperactive deep tendon reflexes
Ophtalmological features	Cortical vision impairment bilaterally	Normal vision	Strabismus
Epilepsy	Yes	Yes	Yes
Age of onset/seizure type	History of infantile spasms, first seizure at 5 m/Type A: tonic clonic; Type B: bilateral arm extension, unsure if seizure activity, limbs jerking and screams involuntarily	1 y, 6 m/myoclonic	New‐born/myoclonic, gelastic, focal clonic
EEG	Poorly organized background, frequent multifocal independent spike foci, poorly organized sleep features	Background rhythm in the first EEG found to be slowed by age. Bilateral frontocentral‐frontotemporal and left hemisphere centroparietal‐parietooccipital regions were found to have phase encounters, spike‐polyspike slow wave activities and generalization of these activities followed by electrodecremental response detected in the last EEG.	In the first EEG, burst suppression pattern detected. Most recent EEG demonstrated that bilateral parietooccipital regions had focused epileptic activity with generalization.
AED received	Levetiracetam, CBD oil	Phenobarbital, levetiracetam	Phenobarbital, levetiracetam
Seizure frequency	Type A: once in 3–4 days, Type B: once a week	Unknown	Unknown
Brain MRI	Enlarged extra‐axial CSF spaces and interfoliate sulci in keeping with cerebral and cerebellar volume loss, microcephaly	Thin corpus callosum, cerebral and cerebellar atrophy, microcephaly	Thin corpus callosum, cerebral and cerebellar atrophy, microcephaly
Congenital malformations/ additional features	Scoliosis, hip dislocation bilaterally, congenital portosystemic shunt incidentally seen on abdominal US, ankle contractures	Low set ears, scoliosis, thin long fingers, overriding toes	Low set ears, scoliosis, thin long fingers, overriding toes, big tongue, ankle contractures, simian line, pit palate, multiple café‐au‐lait spots (2 × 2 cm)

AED, antiepileptic drugs; ASD, autism spectrum disorder; CBD, cannabidiol; CSF, cerebrospinal fluid; EEG, electroencephalography; m, months; MRI, magnetic resonance imaging; UE, upper extremities; y, years.

### Mammalian Elp456 subcomplexes form hetero‐hexameric rings

**Figure EV2:**
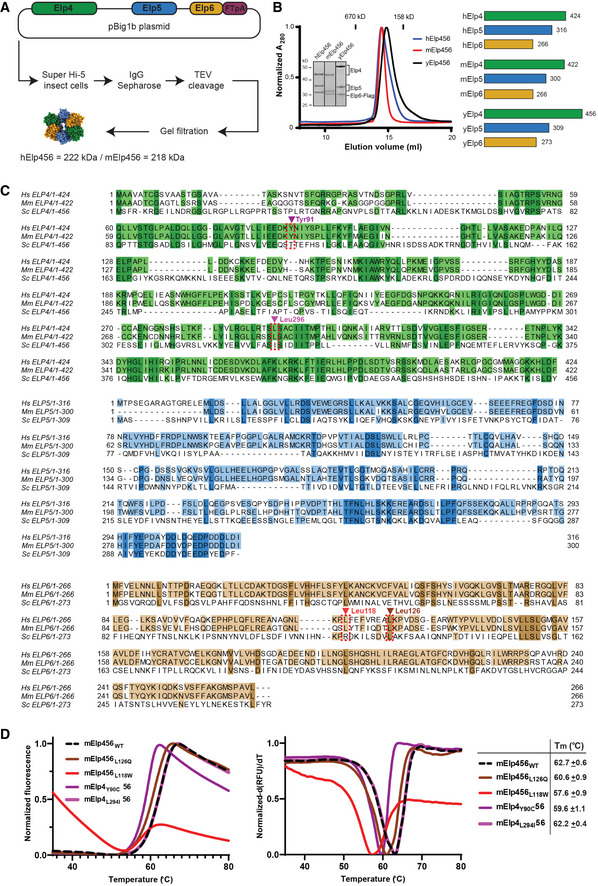
The conservation of human, murine, and yeast Elp456 complexes Workflow scheme for Elp456 protein production from insect cell expression system.SDS–PAGE gel showing the purified human, murine and yeast Elp456 complexes with corresponding gel filtration profiles indicating the estimated elution volume of approximately 200 kDa on the Superose 6 Increase 10/300 GL column (left panel). The schematic overview of Elp4, Elp5, and Elp6 proteins from human, mouse, and yeast with the amino acid length indicated (right panel).The multisequence alignments of Elp4 (green), Elp5 (blue), and Elp6 (brown) proteins show the evolutionary conservation of amino acid sequences.Averaged melting curves from thermal shift assay for mElp456 variants with calculated melting temperatures (Tm) (mean ± SD) on the left and their respective first derivative (right panel), *n* = 3 independent measurements. Source data are available online for this figure.

**Figure 2:**
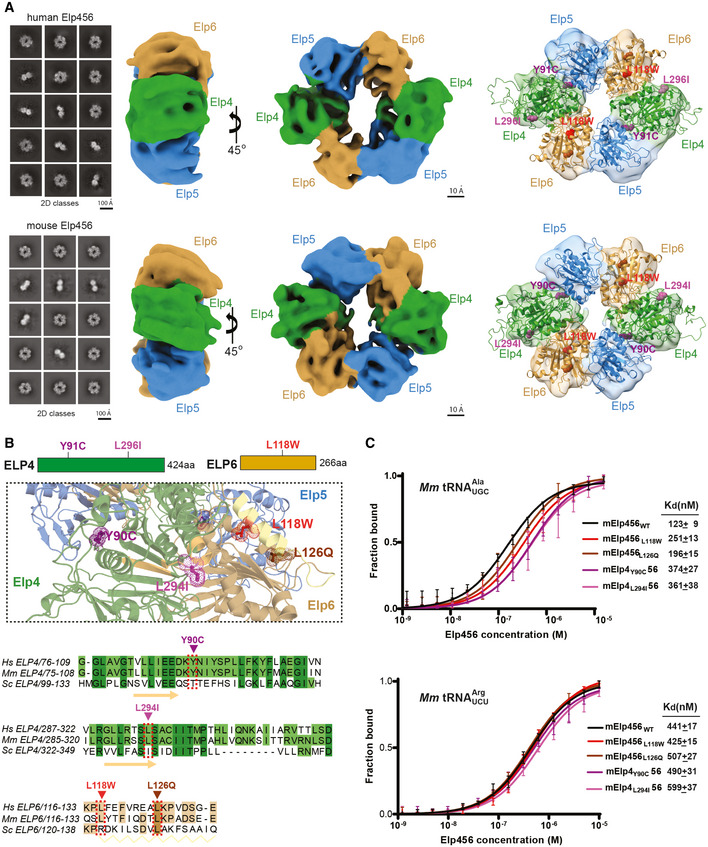
Mammalian Elp456 subcomplexes form tRNA binding assemblies that are affected by *ELP4*/*6* mutations Cryo‐EM analyses of the reconstituted mammalian Elp456 (human—hElp456 and murine—mElp456) complexes. Reference‐free 2D class averages of the human (top) and murine (bottom) Elp456 subcomplexes (left panels, scale bar 100 Å), cryo‐EM maps (middle panels, scale bar 10 Å) and atomic models fit into the cryo‐EM density maps with marked localization of patient‐derived *ELP4* and *ELP6* mutations (right panels).Close‐up view of Elp456 atomic models highlighting the positions of the *Elp6* (L118—red and L126—brown) and *Elp4* (L296/L294—magenta and Y91/Y90—violet) mutations. Elp4 is depicted in green, Elp5 in blue and Elp6 in brown. Mutations are marked on the respective sequence alignments of *Elp6* and *Elp4* with color‐coded arrows.Microscale thermophoresis (MST) analysis of Elp456_WT_ and *Elp4*/*6* variants binding to murine (*Mm*—*Mus musculus*) tRNA^Ala^_UGC_ (top) or *Mm* tRNA^Arg^_UCU_ (bottom) with estimated dissociation constant values (K_d_) and K_d_ fitting errors (mean ± SD), *n* = 3 independent measurements.

In yeast, the Elp456 subcomplex (yElp456) forms a hetero‐hexameric ring which promotes ATP hydrolysis and release of tRNA during crucial steps of Elongator’s reaction cycle (Glatt *et al*, 2012). We employed the BigBac system (Weissmann *et al*, 2016) to simultaneously produce all three subunits of full length human (hElp456) and murine (mElp456) complexes in insect cells. Consequently, we obtained pure, homogenous, and stoichiometric samples of both mammalian subcomplexes (Fig EV2A). The complexes eluted at an estimated molecular weight of ~ 220 kDa, showing no signs of proteolytic cleavage. Hence, hElp456 and mElp456 form a hetero‐hexameric ring harboring two copies of each of the three subunits, like yElp456 (Fig EV2B). Next, we vitrified the purified samples for cryo‐EM single particle analyses (SPA). After particle picking, iterative rounds of 2D and 3D classification and refinement, the obtained datasets resulted in maps of hElp456 at a resolution of 4.32 Å (EMD‐14626) and mElp456 at a resolution of 4.03 Å (EMD‐14627) (Fig 2A; Appendix Fig S1; Appendix Table S2). Our reconstructions have been complicated by a preferred orientation of the ring/disc shape of the Elp456 molecules, which despite various experimental approaches (e.g., addition of various detergents, tilted data collections) could not be circumvented. Based on the known crystal structure of yeast Elp456 (Glatt *et al*, 2012) (PDB 4a8j), we generated atomic models (Bertoni *et al*, 2017) of both human and murine Elp456 subcomplexes (Fig 2A). Despite the limited resolution and the reduced quality of the maps caused by the orientational bias, the models fit unambiguously into the densities, confirming the relative arrangement of subunits in mammals, and creating dimers of trimers with one rather flat and one rather uneven side of the ring (Fig 2A). Due to intrinsic flexibility, we were not able to identify additional densities for the N‐terminus of Elp4 and the predicted C‐terminal domain of Elp5. The high structural similarity of yeast, murine, and human Elp456 indicates a functional conservation of the subcomplex across eukaryotes.

### *Elp4* and *Elp6* mutations affect tRNA binding

Next, we performed *in vitro* analyses of purified mElp456 complexes to assess the molecular consequences of the single amino acid substitutions found in the affected patients. Based on the high amino acid sequence conservation among the eukaryotic Elp4, Elp5, and Elp6 proteins (Fig EV2C), we were able to precisely locate the individual patient‐derived mutations in the obtained atomic models of human and murine Elp456 subcomplexes (Fig 2A and B). The human variants *ELP4Y91C* and *ELP4L296I* correspond to murine *Elp4Y90C*/*L294I* and are positioned in unstructured flexible loop regions located at the interface between Elp4 and Elp5 subunits (Y91C mutation) and Elp4 and Elp6 subunits (L296I mutation; Fig 2A and B). The *Elp6L118W* substitution is found in the α‐helix of the solvent exposed domain of Elp6 (Fig 2B). Notably, the previously described *Elp6* mutation, *Elp6L126Q*, that causes an ataxia‐like phenotype in *wobbly* mice (Kojic *et al*, 2018) is located within the same conserved region of the α‐helix, which likely represents a crucial hotspot for the stability of the Elp456 subcomplex. To define molecular consequences of the patient‐derived mutations, we introduced the substitutions into the murine Elp456 to obtain mElp4_Y90C_56, mElp4_L294I_56, mElp456_L118W_, and mElp456_L126Q_ variants. We managed to purify all variants and subsequently compared their temperature‐dependent unfolding rates to the wild‐type Elp456 (mElp456_WT_; Fig EV2D). Although none of the mutations lead to a complete disassembly of the subcomplex *in vitro*, all Elp456 variants, apart from *Elp4L294I*, displayed a lower thermal stability profile than the wild‐type Elp456. Strikingly, the *Elp6L118W* induced a significant destabilization of the mElp456 ring, as observed by a high fluorescent signal already at low temperatures. In summary, we show that the patient‐derived *Elp4* and *Elp6* variants lead to decreased stability of the isolated murine Elp456 subcomplex.

Next, we tested the affinity of the purified mElp456 variants for *in vitro*‐transcribed and fluorescently labeled murine tRNA^Ala^_UGC_ and tRNA^Arg^_UCU_ carrying the Elongator‐modifiable uridine at position 34 (U_34_; Fig 2C). Using microscale thermophoresis (MST), we observed a noticeable reduction in tRNA binding affinity toward tRNA^Ala^_UGC_ for all tested Elp456 variants up to a three‐fold affinity decrease for the Elp4_L294I_56 variant (K_d_ = 361 ± 31 nM; Fig 2C) in comparison to the Elp456_WT_ (K_d_ = 123 ± 9 nM; Fig 2C). We initially suspected that the reduced thermostability directly leads to the reduced recognition of tRNA molecules. However, we did not observe such a strong correlation between thermostability and the reduction of affinity for the mouse arginine tRNA_UCU_, which is bound by all Elp456 complexes with a lower, but rather similar affinity (K_d_ between 440 and 600 nM). Our results indicate a selective effect of the identified variants in Elp456 subcomplexes toward specific tRNAs species, which can only partially be attributed to the observed decreased thermostability of the complexes *in vitro*.

### *ELP4* and *ELP6* variants impair the function of the Elongator complex

**Figure 3:**
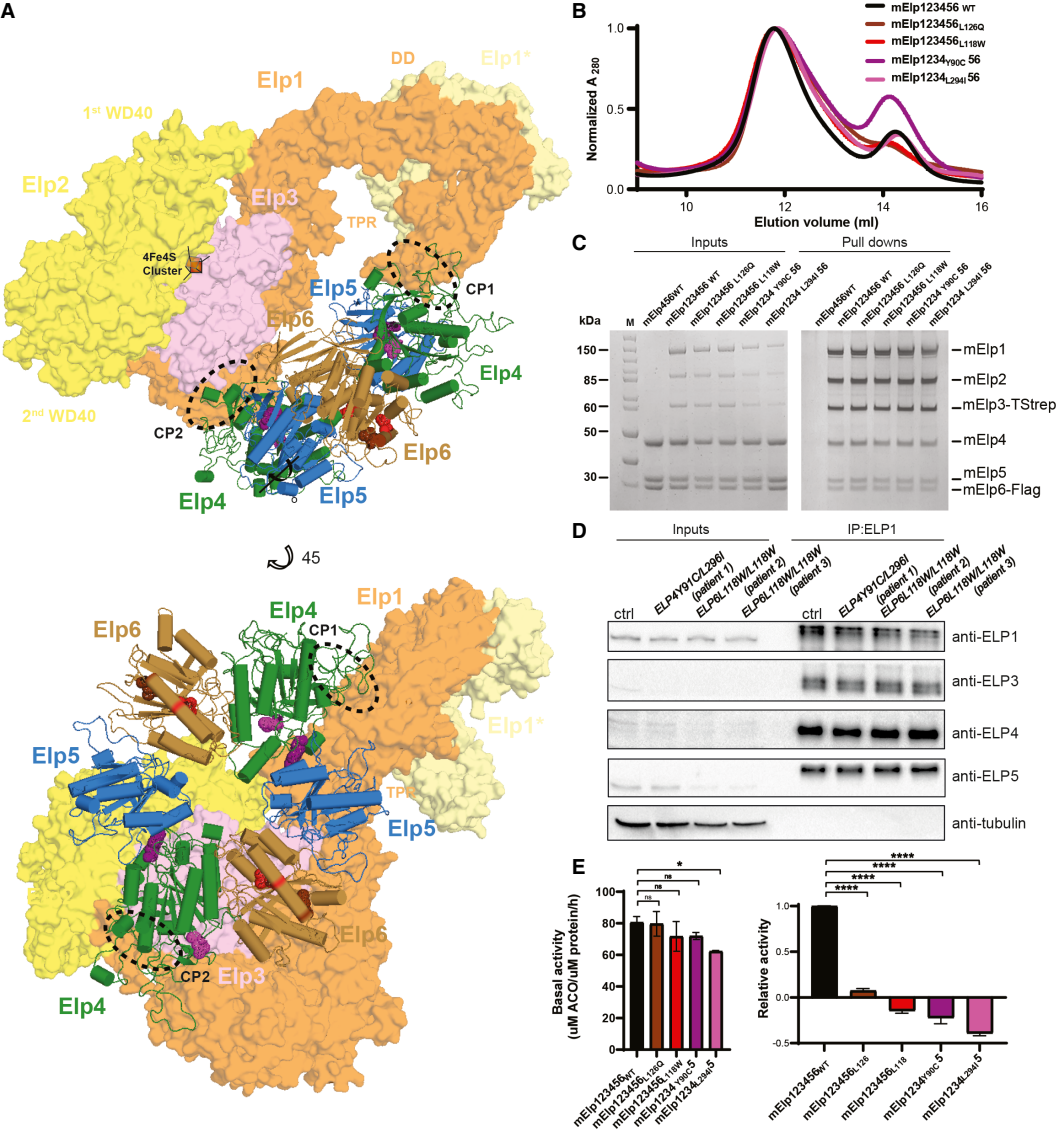
Patient‐derived *ELP4*/*6* mutations inhibit Elongator activity despite the proper complex assembly Model of the murine Elongator complex (Elp123456) prepared using cryo‐EM structure of the yeast Elp123456 (yElp123456; PDB:). The [4Fe4S] cluster of Elp3 subunit is indicated by the orange cube. Murine Elp123 (mElp123) domain organization scheme: WD40—beta‐transducin repeat domain; TPR—tetratricopeptide repeat domain; Elp1*—C‐teminal domain of second Elp1 subunit; DD—dimerization domain of two Elp1 subunits (top); CP1/CP2—predicted contact points between Elp123 and Elp456. Murine Elp123456 model with depicted *ELP4*/*6* mutations (bottom). Color code for molecules: mElp1—orange, mElp2—yellow, mElp3—pink, mElp4—green, mElp5—blue, mElp6—brown, *Elp4* mutations (L296/L294—magenta and Y91/Y90—violet), *Elp6* mutations (L118—red and L126—brown).Gel filtration profiles from Superose 6 Increase 10/300 GL column for all reconstituted Elp123456 variants.Pull‐down experiments of the assembled mElp123456 complexes carrying patients‐derived mutations. The mElp123_WT_ subcomplexes were immobilized on the Dynabeads (strep) via Elp3 protein and incubated with the Elp456 variants. The inputs and pull downs were loaded on the SDS–PAGE gels and visualized by Coomassie staining. The molecular mass marker is indicated on the left (M).Co‐immunoprecipitation analyses of Elongator complexes in patient‐derived fibroblast cell. Whole cell lysates (left) and eluates after immunoprecipitation (right) are shown for control fibroblasts (ctrl) and cultured patient fibroblasts *Elp4Y91C*/*L296I* (patient 1); *Elp6L118W*/*L118W* (patient 2), *Elp6L118W*/*L118W* (patient 3). Used antibodies for immobilization (ELP1) and detection (ELP1, ELP3, ELP4, ELP5, and α‐tubulin) are indicated. *n* = 4 independent experiments.Normalized acetyl‐CoA hydrolysis activity assay for mElp123456 variants incubated either without tRNA (left panel) or with human bulk tRNA (right panel). Statistical analysis: one‐way ANOVA (*α* = 0.05). Statistically significant differences are indicated (*P ≤ 0.05; ****P ≤ 0.0001; ns—not significant), *n* = 3 independent experiments. Data represent mean ± SD.

Elp456 is a component of the Elongator complex and was proposed to act as an ATP‐driven tRNA release factor (Glatt *et al*, 2012). Hence, the decreased stability of Elp456 subcomplexes may indirectly affect the integrity and function of other Elongator subunits and the holoElongator complex (Fig 3A). First, we investigated the impact of the individual mutations on the assembly with the Elp123 subcomplex. We successfully reconstituted the wild‐type complex (Elp123456_WT_) and four variants of the mElp456 subcomplexes *in vitro* via gel filtration co‐migration analyses (Fig 3B). The stoichiometric interaction of all six Elongator subunits was independently confirmed using pull‐down experiments (Fig 3C). Furthermore, we used co‐immuno‐precipitation analyses (Co‐IP) to confirm that the two subcomplexes still assemble in fibroblasts derived directly from the patients (Fig 3D). We also found that the expression of its subunits is not affected by the mutations (Appendix Fig S2A). Our results show that all identified single amino acid substitutions in Elp4 and Elp6 proteins do not negatively influence the expression and interaction of the Elp123 and Elp456 subcomplexes and do not impede the assembly of the holoElongator complex at body temperature (37°C).

The active site of Elp3 around its 4Fe4S cluster (Fig 3A) is spatially rather distant from Elp456 or the identified amino acid substitutions in Elp4 and Elp6. We nevertheless asked whether the *Elp4* and *Elp6* variants (Fig 3B) influence the tRNA‐induced acetyl‐CoA (ACO) hydrolytic activity of the Elp3. We purified sufficient quantities of the reconstituted complexes and tested them in previously established ACO hydrolysis assays (Lin *et al*, 2019; Kojic *et al*, 2021). Strikingly, all four tested mutants showed a slight decrease in tRNA‐independent basal activity and a complete inhibition of the tRNA‐induced ACO hydrolysis in comparison to Elp123456_WT_ (Fig 3E). These results demonstrate that the identified patient‐derived mutations do not prevent the assembly of the Elongator complex, but instead have a detrimental effect on the ability of mElp123 to induce the initial step of the cm^5^ modification reaction *in vitro*.

**Figure 4:**
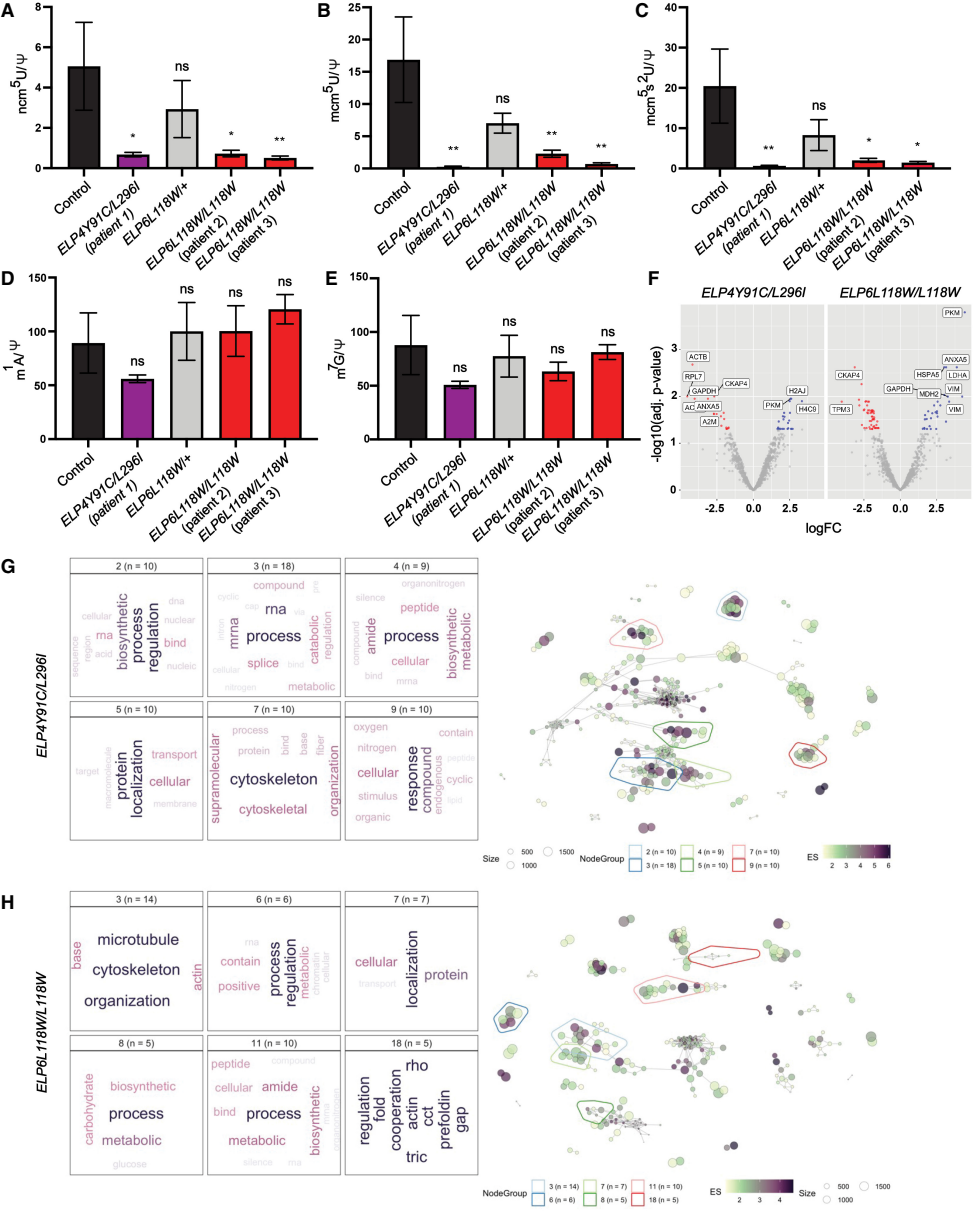
tRNA modification deficiency in fibroblasts derived from patients with *ELP4* and *ELP6* variants High‐performance liquid chromatography (HPLC) coupled to mass spectrometry (MS) used to quantify the Elongator‐dependent tRNA modification 5‐carbamoylmethyluridine (ncm^5^U) in human fibroblasts. Pseudouridine (Ψ) was used as an internal normalization standard. *n* = 3 technical repeats per genotype.HPLC‐MS quantification of Elongator‐dependent tRNA modification 5‐methoxy‐carbonylmethyluridine (mcm^5^U). Pseudouridine (Ψ) was used as an internal normalization standard. *n* = 3 technical repeats per genotype.HPLC‐MS quantification of Elongator‐dependent tRNA modification 5‐methoxycarbonylmethyl‐2‐thiouridine (mcm^5^s^2^U). Pseudouridine (Ψ) was used as an internal normalization standard. *n* = 3 technical repeats per genotype.HPLC‐MS quantification of Elongator‐independent tRNA modification 1‐methyladenosine (m^1^A). Pseudouridine (Ψ) was used as an internal normalization standard. *n* = 3 technical repeats per genotype.HPLC‐MS quantification of Elongator‐independent tRNA modification 7‐methylguanosine (m^7^G). Pseudouridine (Ψ) was used as an internal normalization standard. *n* = 3 technical repeats per genotype.Differential expression (DE) of peptides in fibroblasts obtained from the individuals with *ELP4* and *ELP6* variants relative to control (*n* = 3 technical repeats per genotype). Down‐ (red) and upregulated (blue) peptides are depicted on volcano plots.A network‐based clustering of enriched pathways in *ELP4Y91C*/*L296I* was constructed. Text mining was applied to extract the top terms representing each pathway cluster (left). Network interaction maps are shown (right) with the pathway clusters are marked.A network‐based clustering of enriched pathways in *ELP6L118W*/*L118W* was constructed. Text mining was applied to extract the top terms representing each pathway cluster (left). Network interaction maps are shown (right) and the pathway clusters are marked. Data information: Statistical analysis for data in (A–E): one‐way ANOVA (*α* = 0.05) with a Dunnett’s *post‐hoc* test. Statistically significant differences are indicated (*P ≤ 0.05; **P ≤ 0.01; ns, not significant). Data represent mean ± SEM.

Furthermore, we investigated the impact of the *ELP4* and *ELP6* variants on the activity and function of the complex *in vivo* by analyzing tRNA modification levels in patient‐derived fibroblasts. Both cm^5^U‐dependent modifications, ncm^5^U and mcm^5^U, together with further modified mcm^5^s^2^U, were found to be significantly reduced in the cells originating from *ELP4Y91C*/*L296I* and *ELP6L118W* patients (Fig 4A–C), while Elongator‐independent tRNA modifications were unaffected (Fig 4D and E). Hence, our data confirm that the patient‐derived mutations in *ELP4* and *ELP6* not only diminish Elongator activity *in vitro*, but also impair tRNA modification in human cells.

To further assess the consequences of the reduced U_34_ modification levels on the respective cellular proteomes, we analyzed total protein extracts from the patient fibroblasts using nano‐high performance liquid chromatography (HPLC) tandem mass spectrometry (MS). The analyses revealed altered protein expression levels in the patient cell lines (Fig 4F). As the decoding of only a specific subset of codons is affected by the U_34_ modifications, we have checked for any possible codon bias in the respective mRNA sequences of the mis‐regulated proteins. We have found a preference for certain codons in the respective pools of up‐ and down‐regulated proteins (Appendix Fig S2B). Though, no direct correlation between the modification‐dependent codons and expression levels was observed.

A network‐based clustering of cellular functions associated with the differentially expressed proteins showed an enrichment of pathways involved in translational regulation, RNA‐related processes, and cytoskeleton organization (Fig 4G and H). Given that cytoskeleton is being affected by the variants and the previous studies showing that LOF of Elongator subunits can lead to defects in cell migration (Creppe *et al*, 2009; Close *et al*, 2012), we have tested the patient‐derived fibroblasts in a wound healing assay. After inhibition of proliferation by mitomycin C, we were not able to detect any difference between control fibroblasts and the cells carrying the mutations in *ELP4* or *ELP6* (Appendix Fig S2C). Of note, the functional clusters that appear predominantly perturbed are similar for the *ELP4* and *ELP6* variants. Our data confirm that the respective patient‐derived single amino acid substitutions in the ELP456 subcomplex impact on the proteomic landscape of human cells. Even though for technical reasons our analysis had to focus on patient‐derived fibroblasts, the observed changes on the molecular level confirm the direct link between the variants and noticeable differences in the proteomic signatures of human cells.

### Modeling *Elp6L118W* in mice recapitulates phenotypic features of the patients

**Figure 5:**
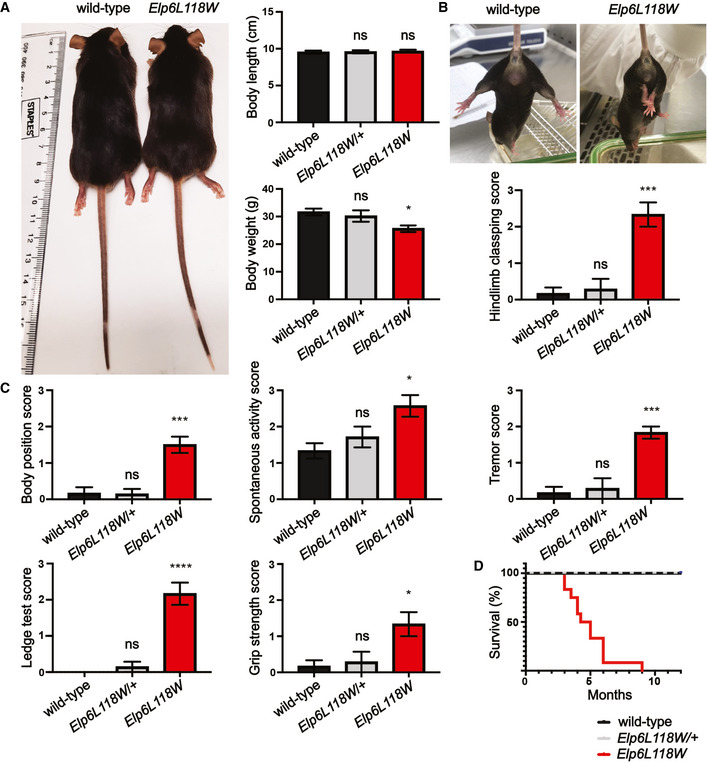
Physical presentation and motor defects in *Elp6L118W* mice Appearance and quantification of body size and weight of adult (2‐months‐old) heterozygous and homozygous *Elp6L118W* mice and littermate controls (*n* = 8 animals per genotype).Abnormal hindlimb clasping of *Elp6L118W* animals. *n* = 6 for wild‐type and *Elp6L118W* mice; *n* = 7 for *Elp6L118W*/+ animals.Significant effects of the *Elp6* mutation on scores for behavioral tests, including body position, spontaneous activity, tremor, ledge test, and grip strength tests. *n* = 6 for wild‐type and *Elp6L118W* mice; *n* = 7 for *Elp6L118W*/+ animals.Kaplan–Meier curve of mouse survival (*n* = 12 per genotype). Data information: Statistical analysis: one‐way ANOVA (*α* = 0.05) with a Dunnett’s *post‐hoc* test. Statistically significant differences are indicated (*P ≤ 0.05; ***P ≤ 0.001; ****P ≤ 0.0001; ns—not significant). Data represent mean ± SEM.

**Figure EV3:**
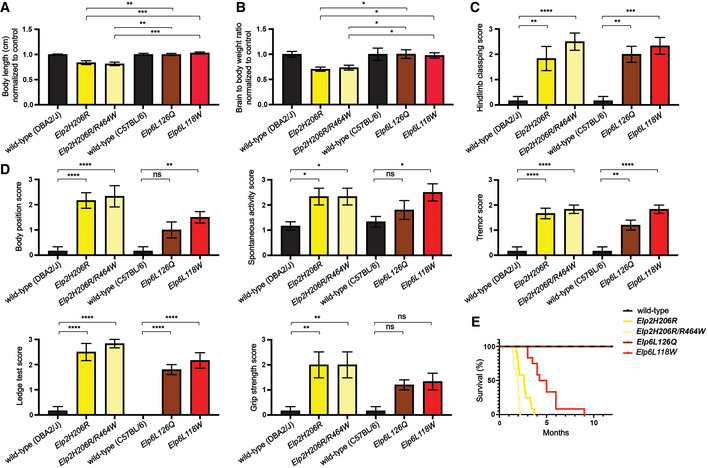
Phenotypic features of *Elp2* and *Elp6* mutant mice Body size of adult (2‐months‐old) *Elp2* and *Elp6* mutant mice and their littermate controls (*n* = 6 for wild‐type (DBA2/J and C57BL/6), *Elp2H206R*, *Elp2H206R*/*R464W*, and *Elp6L118W* animals; *n* = 5 for *Elp6L126Q* animals).Brain weight relative to body weight of adult *Elp2* and *Elp6* mutant mice and their littermate controls (*n* = 6 for wild‐type (DBA2/J and C57BL/6), *Elp2H206R*, *Elp2H206R*/*R464W*, and *Elp6L118W* animals; *n* = 5 for *Elp6L126Q* animals).Abnormal hindlimb clasping of *Elp2* and *Elp6* mutant animals (*n* = 6 for wild‐type (DBA2/J and C57BL/6), *Elp2H206R*, *Elp2H206R*/*R464W*, and *Elp6L118W* animals; *n* = 5 for *Elp6L126Q* animals).Significant effects of *Elp2* and *Elp6* mutations on scores for behavioral tests, including body position, spontaneous activity, tremor, ledge test, and grip strength tests animals (*n* = 6 for wild‐type (DBA2/J and C57BL/6), *Elp2H206R*, *Elp2H206R*/*R464W*, and *Elp6L118W* animals; *n* = 5 for *Elp6L126Q* animals).Kaplan–Meier curve of mouse survival (*n* = 12 per genotype). Data information: Statistical analysis: one‐way ANOVA (*α* = 0.05) with a Dunnett’s *post hoc* test. Statistically significant differences are indicated (*P ≤ 0.05; **P ≤ 0.01; ***P ≤ 0.001; ****P ≤ 0.0001; ns—not significant). Data represent mean ± SEM.

To address potential functional consequences that *ELP6L118W* has on brain development, we used CRISPR‐Cas9 gene editing to introduce the mutation in mice. In contrast to the *Elp2* mutant mice (Kojic *et al*, 2021) and similar to previously reported *Elp6* mutants (*Elp6L126Q*; *wobbly* mice) (Kojic *et al*, 2018), neither global developmental delay nor microcephaly was observed in *Elp6L118W* animals based on body size and brain weight measurements (Figs 5A and EV3A and B). The animals presented with reduced weight likely due to severe motor deficits resulting in reduced feeding ability (Cendelin, 2014). Hindlimb clasping is an indicator of lesions in the motor pathway in several mouse models of neurodegeneration (Lalonde & Strazielle, 2011; Cahill *et al*, 2019), including *Elp2* and *Elp6* mutant mice (Kojic *et al*, 2018, 2021), and was found to be prominent in the *Elp6L118W* animals as well (Fig 5B). A severe motor phenotype was consistently observed across different behavioral tests (Fig 5C). The phenotype consisted of loss of balance and gait coordination (ataxia‐like phenotype), marked tremor, and reduced muscle strength. The mice also showed increased spontaneous activity, frequently found in mouse models of ID and associated NDDs (Verma *et al*, 2019). As reported in the patients, the murine phenotype was confirmed to occur in a recessive manner, given that the phenotype of heterozygous animals corresponded to the one of their littermate controls. The severity of the motor phenotype in the mutant mice imposed a limitation in using cognitive functional tests to assess potential learning and memory deficits, and it was found to be ultimately lethal prior to 9 months of age (Fig 5D).

In agreement with our molecular studies, the *Elp6L118W* mutation appears to have a more severe consequence on the murine phenotype in comparison to *Elp6L126Q*, and it closely resembles the effects of the *Elp2* mutations (Fig EV3C and D). This is also reflected in a shorter lifespan of the *Elp6L118W* animals (Fig EV3E). In summary, the *Elp6L118W* mice display a motor phenotype consistent with the clinical features observed in the patients and mice with previously identified Elongator germline mutations.

### Hippocampal defects and neurodegeneration in *Elp6L118W* mice

To identify brain structures affected by the mutation driving the severe phenotype of the *Elp6L118W* animals, we performed detailed histological analyses at postnatal (P) day 60. The analyses confirmed that no microcephaly was evident, with no significant changes found in the cerebral cortex or hippocampus of the mutant mice (Appendix Fig S3A–C). Similar to our previous findings in the *Elp2* and *Elp6* mutants (Kojic *et al*, 2018, 2021), widespread Purkinje neuron (PN) degeneration was observed in the cerebella of *Elp6L118W* animals (Appendix Fig S3D).

**Figure EV4:**
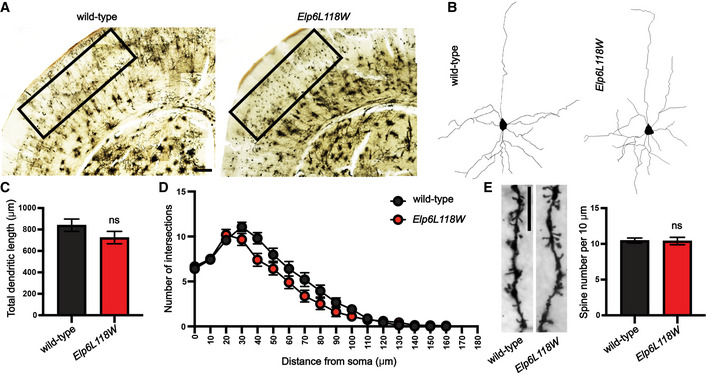
Normal morphology of cortical pyramidal neurons in *Elp6L118W* mice Representative images of Golgi‐Cox‐stained coronal brain sections of adult (2‐months‐old) wild‐type and mutant mice. Rectangles represent somatosensory region containing pyramidal neurons selected for neuron reconstruction and subsequent analyses. Scale bar: 100 μm.Representative pyramidal neuron basal dendritic tree reconstructions.Quantification of total dendritic length from dendritic tree reconstructions shown in (B). *n* = 30; 10 neurons per animal and 3 animals per genotype.Sholl analysis of the complexity of basal dendritic arbors. *n* = 30; 10 neurons per animal and 3 animals per genotype.Representative images of dendritic spines on basal dendrites of the cortical neurons. Quantification of spine density per 10 μm on secondary dendrites is shown (*n* = 15; 5 neurons per animal and 3 animals per genotype). Scale bar: 10 μm. Data information: Statistical analysis: unpaired two‐tailed *t*‐test (*α* = 0.05) with Welch’s correction. Holm‐Sidak correction was applied to adjust for multiple comparisons (D). ns—not significant. Data represent mean ± SEM (with error bars in D removed when smaller than the corresponding data point).

**Figure 6:**
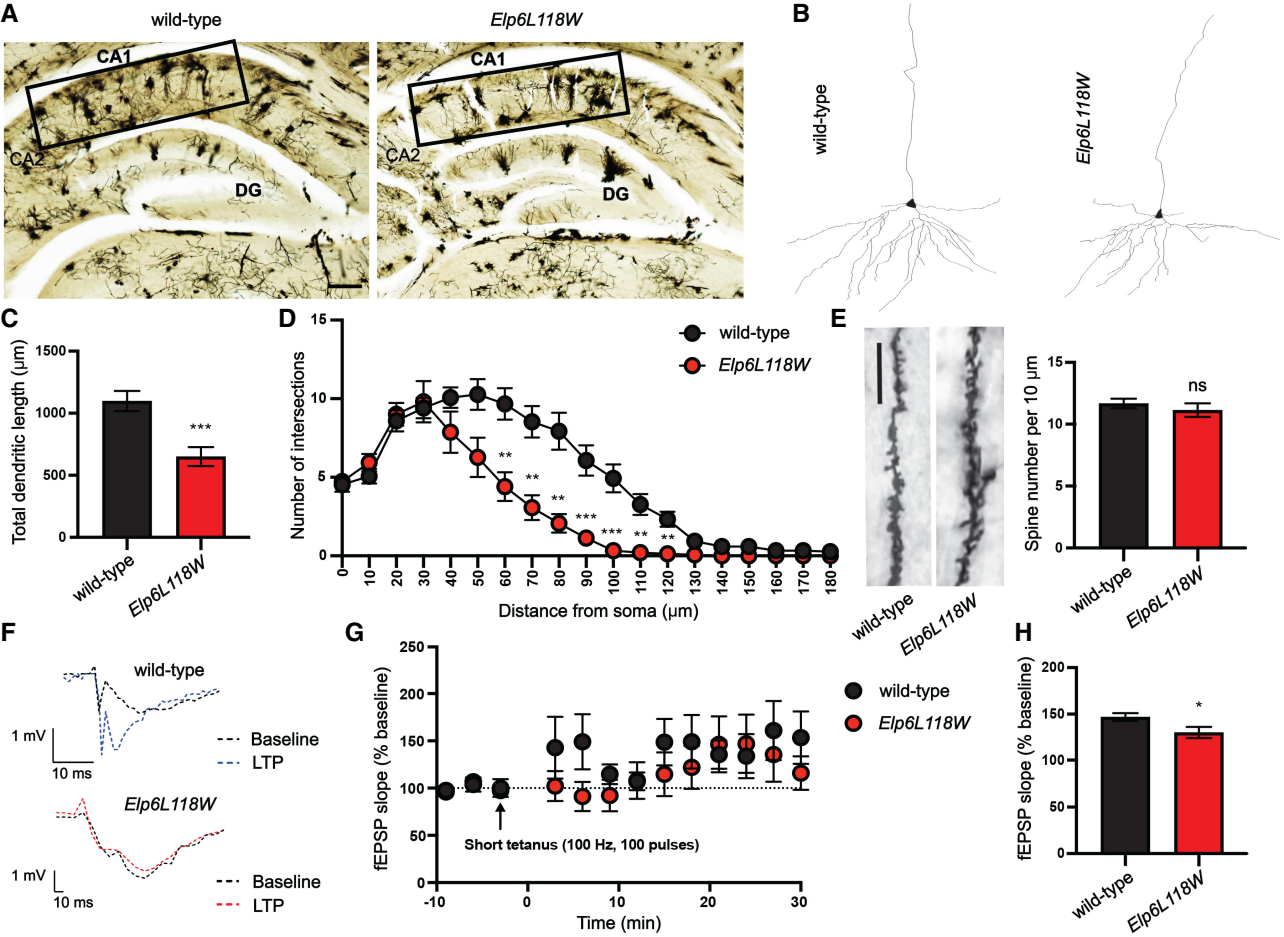
Abnormal morphology and decreased excitability of hippocampal neurons in *Elp6L118W* mice Representative images of Golgi‐Cox‐stained brain sections in the hippocampal area of adult (2‐months‐old) wild‐type and mutant mice. Rectangles represent CA1 region containing pyramidal neurons selected for neuron reconstruction and subsequent analyses. Scale bar: 100 μm.Representative pyramidal neuron basal dendritic tree reconstructions.Quantification of total dendritic length from dendritic tree reconstructions shown in B. *n* = 15; 5 neurons per animal and 3 animals per genotype.Sholl analysis of the complexity of basal dendritic arbors. *n* = 15; 5 neurons per animal and 3 animals per genotype.Representative images of dendritic spines on basal dendrites of the cortical neurons. Quantification of spine density per 10 μm on secondary dendrites is shown. *n* = 15; 5 neurons per animal and 3 animals per genotype. Scale bar: 10 μm.Representative field EPSP (fEPSP) of wild‐type (blue) and *Elp6L118W* (red) mice post high‐frequency stimulation (HFS) on hippocampal CA1 to CA3 regions.Time‐course of long‐term potentiation (LTP) induced by a short tetanus (3 x 100 pulses at 100 Hz; arrow) at hippocampal Schaffer collaterals of mutant and control animals. *n* = 4 animals per genotype.Pooled data of the effect of HFS on the fEPSP slope from (G). *n* = 4 animals per genotype. Data information: Statistical analysis: unpaired two‐tailed *t*‐test (*α* = 0.05) with Welch’s correction. Holm‐Sidak correction was applied to adjust for multiple comparisons (D). Statistically significant differences are indicated (*P ≤ 0.05; **P ≤ 0.01; ***P ≤ 0.001; ns—not significant). Data represent mean ± SEM (with error bars in D removed when smaller than the corresponding data point). CA1, cornu ammonis 1; CA2, cornu ammonis 2; DG, dentate gyrus; EPSP, excitatory postsynaptic potential.

Next, we screened for potential neurogenesis defects in the cortical and hippocampal structures by performing Golgi‐Cox staining to assess neuronal architecture. Pyramidal neurons of the II and III layers of the somatosensory cortex (Fig EV4A) and CA1 pyramidal neurons in the hippocampus were selected for analysis (Fig 6A) due to their high susceptibility to neurodegeneration and disease (Anand & Dhikav, 2012; Till *et al*, 2012). While no morphological changes were found in the cortical neurons (Fig EV4B–E), the analysis of traced hippocampal pyramidal neurons showed a significant reduction in both the length (Fig 6B and C) and branching (Fig 6D) of the dendrites in the *Elp6* mutant animals, with no alternations in the dendritic spine density (Fig 6E).

To further explore functional consequences of the morphological abnormalities found in the hippocampal neurons, we investigated synaptic transmission at the Schaffer collateral to CA1 pyramidal neuron synapse. Schaffer collaterals were electrically stimulated, and field excitatory postsynaptic potential (fEPSP), which reflects a summation of excitatory postsynaptic responses, was recorded after applying high‐frequency stimulation (HFS) to trigger long‐term potentiation (LTP). Remarkably, reduced fEPSP was recorded in *Elp6L118W* mice relative to littermate controls (Fig 6F–H), suggesting defects in basal synaptic transmission affecting their learning abilities and memory (Gruart *et al*, 2006; Whitlock *et al*, 2006).

Immunofluorescence‐based histological analysis was performed to analyze interneurons given their essential role in memory and cognition (Kirkcaldie, 2012), and previously identified interneuron defects in Elongator mouse models (Creppe *et al*, 2009; Tielens *et al*, 2016; Kojic *et al*, 2021). Interneurons were found not to be affected in the *Elp6* mutants (Appendix Fig S4A and B). In addition, we screened for potential myelination impairment in the mutant mice, as this was evident on MRI scans of the patients and have also been associated with *Elp2* (Kojic *et al*, 2021) and *Elp3* (Bento‐Abreu *et al*, 2018) pathogenic variants in both human and mice. No difference was observed in the myelination of the *Elp6* mutant and control mice (Appendix Fig S4C). Thus, no interneuron or myelin abnormalities can be attributed to the underlying pathology in the *Elp6L118W* mice.

### Compromised activity of the Elongator complex in *Elp6L118W* mice

PN degeneration in *Elp6L118W* mice was found to occur in a more progressive manner than in the *wobbly* mice, with the *Elp6L118W* cerebellum being almost fully depleted of PNs by P60 whereas the *wobbly* mice lose half of the PNs by this age (Kojic *et al*, 2018) (Appendix Fig S5A). We sought to explore the cause of PN degeneration and whether unfolded protein response (UPR) is a prominent feature of these neurons and the cause of their death as previously identified in the *Elp2* (Kojic *et al*, 2021) and *Elp6* (Kojic *et al*, 2018) mutant mice. Indeed, we found UPR and ER‐stress‐mediated apoptosis to be a mode of PN death in these animals (Appendix Fig S5B–D). As we did not find evident microcephaly and cortical defects in the *Elp6* mutants, we hypothesized that UPR was limited to the PNs. To test this, we analyzed the expression of one of the main transcriptional effectors of UPR, activating transcription factor 4 (Atf‐4), previously linked to neurogenesis defects in the murine Elp3‐depleted cortical neurons (Laguesse *et al*, 2015). Indeed, UPR was not evident in the cerebral cortex of the *Elp6L118W* animals (Appendix Fig S6A). Moreover, we found that ATF‐4 expression was not affected in the patient fibroblasts as well (Appendix Fig S6B).

**Figure EV5:**
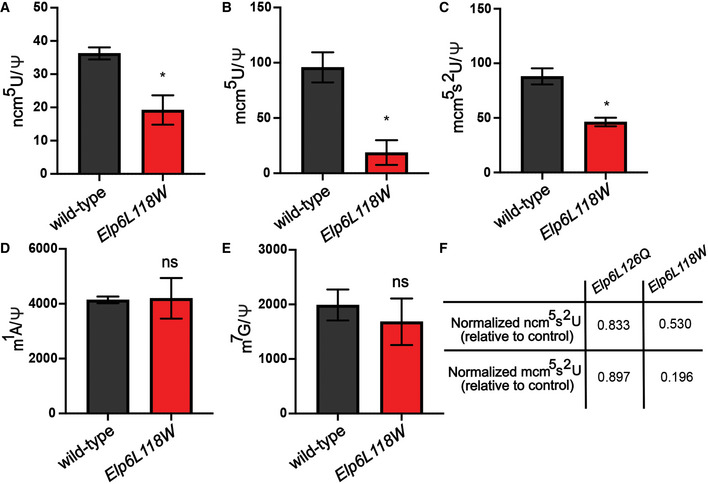
tRNA modification deficiency in *Elp6L118W* murine brain High‐performance liquid chromatography (HPLC) coupled to mass spectrometry (MS) used to quantify the Elongator‐dependent tRNA modification 5‐carbamoylmethyluridine (ncm^5^U) in the brain tissue of adult (2‐months‐old) *Elp6L118W* relative to wild‐type animals. Pseudouridine (Ψ) was used as an internal normalization standard. *n* = 3 animals per genotype.HPLC‐MS quantification of Elongator‐dependent tRNA modification 5‐methoxy‐carbonylmethyluridine (mcm^5^U). Pseudouridine (Ψ) was used as an internal normalization standard. *n* = 3 animals per genotype.HPLC‐MS quantification of Elongator‐dependent tRNA modification 5‐methoxycarbonylmethyl‐2‐thiouridine (mcm^5^s^2^U). Pseudouridine (Ψ) was used as an internal normalization standard. *n* = 3 animals per genotype.HPLC‐MS quantification of Elongator‐independent tRNA modification 1‐methyladenosine (m^1^A). Pseudouridine (Ψ) was used as an internal normalization standard. *n* = 3 animals per genotype.HPLC‐MS quantification of Elongator‐independent tRNA modification 7‐methylguanosine (m^7^G). Pseudouridine (Ψ) was used as an internal normalization standard. *n* = 3 animals per genotype.Table of comparison of ncm^5^U and mcm^5^U levels in the brain tissue of *Elp6L126Q* and *Elp6L118W* mice. *n* = 3 animals per genotype. Data information: Statistical analysis: unpaired two‐tailed *t*‐test (*α* = 0.05) with Welch’s correction. Statistically significant differences are indicated (*P ≤ 0.05; ns—not significant). Data represent mean ± SEM.

To assess the molecular consequences of the mutation behind the neuropathology in mice, we performed biochemical analyses of the murine brain tissue. Western blot analysis revealed that the mutation impaired the expression of the Elp6 protein, while other Elongator subunits seemed to be unaffected (Appendix Fig S6C), which is in agreement with our *in vitro* data. Consistent with our findings in the human cells, we detected reduced levels of Elongator‐dependent tRNA modifications in the *Elp6L118W* brains (Fig EV5A–E). The modification of tRNA is reduced to a higher extent in the *Elp6L118W* than *wobbly* mice (Fig EV5F), further confirming the more detrimental effect of the clinically relevant mutation on the function of the holoElongator complex.

## Discussion

Elongator functions as a fundamental translational regulator via modifying U_34_ in tRNAs and has been implicated in a wide range of neurodevelopmental activities (Kojic & Wainwright, 2016). Pathogenic variants in Elongator subunits have been associated with a spectrum of NDDs, but existing functional studies address almost exclusively variants in the catalytic ELP123 subcomplex. This study explores instances of clinical pathogenic variants in the accessory ELP456 subcomplex modeled *in vitro* and in mice, correlating clinical observation with the molecular impact of the variants on the function of the complex in maintaining proper tRNA modification levels.

In yeast, deletion of any of the six Elongator subunits leads to identical phenotypes and loss of Elongator‐dependent tRNA modifications (Huang *et al*, 2005), which can be rescued by the overexpression of specific modified tRNA species (Esberg *et al*, 2006). Studies in mammals predominantly focused on the role of the enzymatically active Elp3 subunit. Our work on clinically derived ELP4/6 variants finally permits a fair and direct comparison between Elp123 and Elp456 variants and their role in neurodevelopment and disease. Strikingly, our study shows that in contrast to unicellular yeast, the two subcomplexes in higher eukaryotes presumably fulfill cell‐type specific functions.

Although the patients with newly identified *ELP4* and *ELP6* pathogenic variants have common clinical features and brain MRI findings with recently reported patients with *ELP2* pathogenic variants (Kojic *et al*, 2021), modeling the variants in mice revealed that different neuropathology drives the disease. PN degeneration is consistently found across different Elongator mutations (Kojic *et al*, 2018, 2021). However, in contrast to *Elp2* mutant animals, no signs of microcephaly and no defects in myelination and development of cortical projection neurons and interneurons have been identified in *Elp6L118W* mice. Synapse dysfunction, branching, and dendritic length abnormalities, found in the hippocampal neurons of the *Elp6L118W* mice, have not been reported in any of the previously identified Elongator mutants. In summary, although mutations in the Elp123 are known to affect the neuronal activity across different brain regions, the Elp456 ring is almost completely dispensable for some of these neurons. Nonetheless, Elp456 appears to be crucially important for pyramidal and Purkinje neurons in the hippocampus and cerebellum, respectively.

Hence, mutations in two subcomplexes may differently affect the brain cells, with those in the hexameric ring being less detrimental for the brain development than the mutations in the catalytic subcomplex. Neurons are known to be vulnerable to the accumulation of misfolded proteins and perturbation in the cellular activities shown to be driven by Elongator mutations (Kojic & Wainwright, 2016). PNs and hippocampal neurons are particularly sensitive to different kinds of physiological stress due to their enhanced metabolic demand relative to other neurons (Jiang *et al*, 2004; Kagias *et al*, 2012; Shetty *et al*, 2012; Lucas *et al*, 2015). Thus, perturbations in the Elp456 ring seem to be sufficient to cause defects in these neurons, and any disruption in the catalytic Elp123 results in brain‐wide impairment.

Our complementary *in vitro* analyses aim to resolve the role of Elp456 in higher eukaryotes and provide first molecular explanations for the observed differences between Elp123 and Elp456. Our biochemical experiments using purified mouse Elp456 show that mutations in Elp456 affect the binding to certain tRNA molecules, whereas the same mutations seem to have a much smaller impact on the interaction with other modifiable tRNAs. We speculate that these differences might be linked to the varying impact of the mutations on specific neuronal cell types. If tRNAs affected by mutations in Elp456 are not produced in certain cell types, the effect of the mutations might simply be irrelevant. Hence, it would also be of high interest to correlate our observations with cell‐type specific tRNA iso‐decoder expression data *in vivo* once reliable data on neuronal subtypes becomes available in the future.

While clinically relevant mutations found in the Elp123 subcomplex reduced the ACO hydrolysis activity of Elp123 by ~ 40%, Elp456 mutations almost completely abolish this activity *in vitro*. This observation is consistent with an almost complete absence of Elongator‐dependent modifications in the tested patient‐derived fibroblast cells carrying ELP4/6 pathogenic variants. We speculate that disease relevant mutations in Elp123 need to permit a moderate activity level of Elongator, because residual tRNA modification levels are required for development and survival. Stronger mutations or disintegration of Elp123 would lead to lethality, as shown for deletions of Elongator genes in mice (Chen *et al*, 2009; Bento‐Abreu *et al*, 2018; Kojic *et al*, 2018, 2021). Disease‐associated mutations in Elp456 may still support the assembly of the complex at physiologically relevant temperatures, but lead to a greater reduction of activity, because its action is required only for a subfraction of neuronal cell types during development.

Consistent with studies of the catalytic subcomplex in model organisms (Laguesse *et al*, 2015; Nedialkova & Leidel, 2015; Kojic *et al*, 2021), *ELP4*/*6* pathogenic variants perturb the integrity of the human proteome. The proteomic analyses and functional association identified the perturbation of cytoskeleton organization pathways, which could directly account for morphological defects of the hippocampal neurons. Of note, fibroblasts are fast‐dividing cells and thus are more likely to tolerate alternations in translation and biosynthetic pathways. Hence, the patient‐derived fibroblasts also showed no observable differences in cell migration. In contrast, similar changes in neurons might lead to more severe consequences, which could explain why they are particularly vulnerable to the detected pathogenic variants. Yet, the selective vulnerability of hippocampal over cortical neurons to these alternations remains elusive. We currently believe that these changes most likely occur due to tRNA selectivity as well as cell‐type specific codon usage and the altered expression of specific key proteins. Foremost, the changes of the proteomic signatures confirm that patient‐derived cell lines change their proteomic landscape, which we can directly link to the reduced activity of the Elongator complex and the change of tRNA modification patterns.

In summary, our study characterizes clinically relevant mutations in the accessory Elongator subcomplex *in vitro* and *in vivo*. Our results further highlight the crucial role of the complex in the emerging neuroepitranscriptome (Shafik *et al*, 2021) that drives development and maintenance of the CNS. We provide a direct link between defective Elp456, compromised function of the complex, and a complex neurological phenotype and reveal unanticipated differences between the two subcomplexes in the brain. Further studies of different neuronal types and their specialized proteomes in Elongator mutants will be essential to decipher these findings also on the molecular level.

## Materials and Methods

### Clinical data collection

The study was approved by the Research Ethics Board at The Hospital for Sick Children (project license number 1000070283) and the Dokuz Eylul University Clinical Research Ethics Committee (project license number 490‐SBKAEK 2019/14‐05). Patients with the compound heterozygous variants in *ELP4* and the homozygous variant in *ELP6* were recruited from the clinical research programs in Canada and Turkey, respectively. All patients were clinically evaluated by pediatric neurologists and medical geneticists at their respective healthcare centers. Clinical data were collected from the electronic patient charts using standardized questionnaires and interviews with patients and their parents. Brain MRI images were reviewed by a neurologist and a neuroradiologist. Parents provided written informed consent for publication of clinical pictures. Written informed consent was obtained from all subjects and the experiments conformed to the principles set out in the WMA Declaration of Helsinki and the Department of Health and Human Services Belmont Report. Patients underwent skin biopsies as a part of their clinical diagnostic investigations and their previously banked cultured skin fibroblasts were obtained for functional analysis. Fibroblast analyses were approved by the University of Queensland Institutional Human Research Ethics Committee (project license number 2020000858).

### DNA constructs

Genes encoding Elp4, Elp5, and Elp6 from *Homo sapiens* (*hELP456*) and *Mus musculus* (*mELP456*) were cloned into pFastBac1 HTa plasmids with Flag‐TEV‐ProteinA tag sequence localized on the 3′ side of *ELP6* genes. *ELP123* and *ELP456* constructs were generated using Gibson assembly method. In detail, *ELP1* genes were cloned into pFastBac1, *ELP2* into pFastBac1 HTa with the 6 × His tag sequence, and *ELP3* into pFastBac1 with additional Twin‐Strep‐tag sequence on the 3’ side of the gene and assembled into pBig1a expression vector. *ELP4*, *ELP5*, and *ELP6* genes were assembled into pBig1b expression vector. All six genes were amplified in PCR with primers adding overhangs on 5′ and 3′ sides of each gene and then assembled within pBig1a or pBig1b plasmids using standard protocol and primers (Weissmann *et al*, 2016). Mutations in *mELP4* and *mELP6* were introduced by QuickChange mutagenesis. A list of primers used for cloning and mutagenesis is provided in Appendix Table S1. All genetic constructs were moved to insect cells using standard Bac‐to‐Bac protocols.

### Recombinant protein production and purification

For Elp456 protein expression, Hi‐5 cells were infected with MOI = 1 and grown for 3 days at 27°C on a shaking platform. The Elp123 subcomplex was expressed in the Sf9‐1 cells. Subsequently, insect cells were lysed in Lysis Buffer (for Elp456: 50 mM HEPES pH 7.5, 150 mM NaCl, 2 mM MgCl_2_, 2 mM DTT, 5% glycerol,  DNase I, protease inhibitors; for Elp123: 50 mM HEPES pH 7.5, 100 mM NaCl, 2 mM DTT, 5% glycerol, DNase I, protease inhibitors) by 3 rounds of freezing and thawing in liquid nitrogen and sonication, followed by two‐step centrifugation (4°C, 1 h, 80,000 × *g*). Elp456 supernatants were purified on IgG agarose beads (Merck) followed by overnight TEV cleavage in Cleavage Buffer (50 mM HEPES pH 7.5, 150 mM NaCl, 2 mM MgCl_2_, 2 mM DTT). On the next day, the protein sample was run on S200 Increase 10/300 GL column (GE Healthcare) in Final Buffer (20 mM HEPES pH 7.5, 100 mM NaCl, 2 mM MgCl_2_, 5 mM DTT). Elp123 variants were purified using StrepTrap HP 5 ml column (GE Healthcare) eluted in Strep Elution Buffer (50 mM HEPES, 100 mM NaCl, 1 mM DTT, 5% Glycerol, 5 mM d‐desthiobiotin, pH 8.0), followed by affinity chromatography on HiTrap Heparin HP 5 ml column (GE Healthcare) eluted in a gradient of Heparin Elution Buffer (50 mM HEPES, 1,000 mM KCl, 1mM DTT, pH 8.0). Lastly, eluates were run on Superose 6 Increase 10/300 GL column (GE Healthcare) in Final Buffer (20 mM HEPES pH 7.5, 100 mM NaCl, 5 mM DTT).

### Elp123456 reconstitution and *in vitro* pull‐down

The mElp123_WT_ subcomplexes were incubated with the Elp456 variants for 30 min at 25°C in 2:1 ratio, followed by purification step on the Superose 6 Increase 10/300 GL column (GE Healthcare) in Final Buffer (20 mM HEPES pH 7.5, 100 mM NaCl, 5 mM DTT).

For pull‐down experiments, the mElp123_WT_ subcomplexes were immobilized on the Dynabeads MyOne Streptavidin C1 (ThermoFischer) via Twin‐Strep‐tagged Elp3 protein, incubated with the Elp456 variants for 30 min at 4°C, washed 3 times in the Wash Buffer (20 mM HEPES pH 7.5, 100 mM NaCl, 1 mM DTT, 0.05% Tween 20), and eluted from the beads in Elution Buffer (20 mM HEPES, 100 mM NaCl, 1 mM DTT, 5 mM d‐desthiobiotin, pH 8.0). The inputs and pull downs were loaded on the SDS–PAGE gels and visualized by Coomassie staining.

### Electron microscopy

Purified Elp456 subcomplexes were applied to glow discharged carbon‐coated cryo‐EM grids (Quantifoil R 1.2/1.3 copper, 200 mesh). A total of 2.5 μl of sample (0.58 mg/ml) was plunge‐frozen using FEI Vitrobot Mark IV set to 100% humidity and 4°C (blotting parameters: wait time: 15 s; blot force: 5 s; blot time, 8 s). Micrographs were acquired at 300 kV using FEI Titan Krios equipped with a Gatan Quantum energy filter and a K2 Summit direct detector. The detector was operated in super‐resolution mode at ×105,000 magnification, resulting in a pixel size of 0.86 Å on the object scale. A total of 4,121 micrographs were collected for the mElp456 reconstruction, 11,472 for the hElp456 reconstruction. Datasets were collected at under‐focus varying between 1 and 2.5 μm a total of 40 frames accumulating to doses of 41 e−/Å2.

### Image processing

Frame alignment and dose weighting were performed with MotionCor2 (using Fourier space cropping by a factor of 2, resulting in a pixel size of 1.72), and contrast transfer function was determined with CTFIND4. Total number of 336,000 (mElp456) and 209,000 (hElp456) particles were template‐based picking in Cryosparc v3.2.0. Classification, refinement, post‐processing, and local resolution estimation were performed with RELION3.1 and Cryosparc v3.2.0. Particles were subjected to reference‐free classification, from which 288,000 particles (86%) for mElp456 and 176,000 particles (84%) for hElp456 were selected for subsequent processing. Masked 3D classification was performed with the selected unbinned particles. Major classes containing more than 80% of particles were selected with recognizable Elp456 ring‐like shape and refined. After post‐processing, we obtained the mElp456 map with a resolution of 4.03 Å and 252,000 particles. For the hElp456 subcomplex, the classification and refinement yielded the map at a resolution of 4.32 Å. These maps were refined with C2 symmetry, applying a mask that covered the whole molecule. Cryo‐EM maps were prepared using ChimeraX (Goddard *et al*, 2018; Pettersen *et al*, 2021).

### Structure modeling

Elp4, Elp5, and Elp6 protein atomic models were predicted by homology to previously solved structure with SWISS‐MODEL server (Bertoni *et al*, 2017). The mElp123456 structural model was created with SWISS‐MODEL using yeast Elp123456 cryo‐EM structure as a template (PDB). All models were made in PyMOLv2.1 (Schrödinger, 2018).

### Thermal shift assay

Protein samples were concentrated to 2 g/l in 20 mM HEPES, 150 mM NaCl, 5 mM DTT, pH 7.5, and mixed with 25 × SYPRO Orange (ThermoFisher) on a 96‐well qPCR plate. CFX96 Real‐Time System C1000 Touch Thermal Cycler (Bio‐Rad) was used to gradually heat samples from 4 to 98°C with at heating rate of 0.2°C/10 s. The fluorescence intensity was measured at probe‐specific excitation (470 nm) and emission (570 nm) wavelengths. The graphs were prepared in Prism v8.0.2 (GraphPad) software.

### Microscale thermophoresis for tRNA binding assay

The internally Cy5‐labled murine (*Mm*—*Mus musculus*) tRNA^Arg^_UCU_ (0.2 μM) or *Mm* tRNA^Ala^_UGC_ (0.3 μM) were incubated with 16 serial dilutions of mElp456 variants (starting from 10 μM) in MST Buffer (20 mM HEPES, 100 mM NaCl, 5 mM DTT, pH 7.5, 0.0125% Tween 20). Measurements were performed at 60% excitation power in Premium Coated capillaries on the Monolith NT.115 (Nanotemper Technologies) at 37°C. Obtained data were analyzed and dissociation constant values were calculated using MO. Affinity software (Nanotemper Technologies) from at least three independent experiments. The graphs were prepared in Prism v8.0.2 (GraphPad) software.

### Acetyl‐CoA hydrolysis assay

Each of the Elp123456 variants in concentration of 0.3 μM was mixed with 2 μM human bulk tRNA in presence of 100 μM acetyl‐CoA in 1x acetyl‐CoA assay buffer and incubated in a thermocycler for 3 h at 37°C. Next, the samples were passed through a 3 kDa cutoff concentrator (EMD Millipore). Flow‐through was collected and pipetted into a 96‐well plate. Acetyl‐CoA quantity in each sample was determined with Acetyl‐CoA Assay Kit (MAK039, Merck) according to the manufacturer’s instructions. Fluorescence intensity was measured on a plate reader (TECAN) at probe‐specific excitation (535 nm) and emission (587 nm) wavelengths. Hydrolysis rates were calculated from at least three independent experiments. The graphs were prepared in Prism v8.0.2 (GraphPad) software.

### Cell culture

Fibroblasts from *ELP4Y91C*/*L296I* and *ELP6L118W* patients and mother (*ELP6L118W*/+) were obtained through punch biopsy, while control fibroblasts (TIG‐102) were purchased from JCRB Cell Bank. Dulbecco’s Modified Eagle’s Medium (11995065, ThermoFisher) with 10% FBS (10099141, ThermoFisher) and added penicillin/streptomycin (15070063, ThermoFisher) was used to culture the cells at 37°C with 5% CO_2_. The fibroblasts were harvested, diluted, and plated to obtain single cell‐derived clones. The clones were expanded in culture for 2 to 4 additional passages and dissociated by trypsinization (25200072, ThermoFisher) and harvested for western blot, proteomic, and tRNA modification analyses (approximately 3 × 10^6^ cells per sample).

### Wound healing scratch assay

A total of 35,0000 cells per well were seeded in a 12‐well plate with special inserts (Ibidi). One hour before analysis, cells were treated with 40 μg/ml mitomycin C to inhibit cell proliferation. Next, medium was removed and replaced with fresh one. After removal of the insert, image acquisition was performed under standard culture condition (37°C; 5% CO_2_) using a Leica DMI6000B microscope (10× magnification). Pictures were collected every hour for a period of 24 h. Data analyses and quantification of the normalized wound area were done using Fiji ImageJ.

### Co‐immunoprecipitation (Co‐IP)

Confluent cells were collected from a 100 mm Petri dish, washed with ice‐cold PBS, and lysed with 100 μl lysis buffer (0.5% TRITON X‐100, 50 mM Tris/HCl pH 7.5, 1 mM EDTA, 150 mM NaCl, PMSF), sonicated and centrifuged for 10 min at 4°C (14,000 *g*). Overall protein concentration in the samples was determined by BCA Assay kit (ThermoFisher Scientific). For each IP experiment, 500 μg total protein was used. A lysate pre‐clearing was performed using 20 μl protein A magnetic beads (Cell Signaling), incubated and rotated for 20 min at RT. Subsequently, the supernatant was collected from the beads and ELP1 antibody (Cell Signaling; #5071) was added at 1:50 dilution in a final volume of 200 μl and incubated at 4°C, overnight. Pre‐washed protein A magnetic beads (20 μl) were mixed with lysate‐ELP1 antibody, incubated by rotation for 20 min at RT. Next, beads were washed 3 times with the lysis buffer, resuspended in 15 μl 1x SB buffer, and boiled at 95°C for 5 min. Subsequently, samples were subjected to western blot analysis.

### Western blot analysis (Co‐IP samples and ATF‐4 in human fibroblasts)

For SDS–PAGE electrophoresis, 15 μg total protein (input samples) and 25% of IP eluates were loaded on the gel. After the semi‐dry transfer into 0.2 μm PVDF membrane, membranes were blocked for 1 h in 5% milk or 5% BSA. Primary antibodies used: anti‐ELP1 (1:1,000 dilution; ThermoFisher Scientific; PA5‐20286), anti‐ELP3 (1:500 dilution; Cell Signaling; # 5728S), anti‐ELP4 (1:500 dilution; Biotechne; NBP2‐16322), anti‐ELP5 (1:500 dilution; ThermoFisher Scientific; PA5‐54745), anti‐ATF4 (1:1,000; Abcom; ab184909); anti‐α‐tubulin (1:1,000; Sigma Aldrich; CP06). Membranes were incubated with primary antibodies at 4°C, overnight, followed by incubation with horseradish peroxidaseq‐coupled secondary antibodies (anti‐mouse or anti‐rabbit at 1:2,000 dilution for 1 h, RT). For detection, chemiluminescent substrate was used and membranes were developed on ChemiDoc XRS system (Bio‐Rad).

### Animals and genotyping

All experimental procedures conducted on mice were approved by the University of Queensland Molecular Biosciences Ethics Committee (project license numbers IMB/125/19 and IMB/416/19). Mice were housed under standard conditions, a 12‐h light/dark cycle with food and water available *ad libitum*, at ambient temperature (22 ± 2°C) with relative humidity (30–60%). *Elp6L118W* mice were generated using CRISPR/Cas9 on C57BL/6 genetic background.

Genomic DNA for genotyping purposes was obtained from toe clips using QuickExtract DNA extraction solution (Epicenter) as per manufacturer’s instructions. A custom TaqMan SNP genotyping assay (Applied Biosystems) was used to genotype the animals in a qPCR reaction based on allelic discrimination. Genotyping primers are included in Appendix Table S1. Cycling conditions used were 95°C for 10 min, followed by 40 cycles of denaturation at 92°C for 15 s and annealing and extension at 60°C for 1 min. The amplified product of 626 bp was further annealed with VIC‐labeled wild‐type or FAM‐labeled *Elp6L118W* sequence (Appendix Table S1).

### Behavioral testing

Animals were tested between P60 and P70 according to the modified SHIRPA screen (v1.04: 2002.3) (Rogers *et al*, 1997) and previously published simple composite phenotype scoring system for evaluating mouse models of spinocerebellar ataxia (Guyenet *et al*, 2010). All tests were repeated three times and scored as described below on a scale 0–3 (0–2 for tremor).

*Body position*: A mouse was placed in a cylindrical viewing jar (15 cm diameter) and observed for 2 min. Following scores were assigned.

A score of 0: The mouse is frequently rearing on hind legs.
A score of 1: The animal attempts to rear but is unable to do so as it loses balance.
A score of 2: Sitting or standing, no vertical activity (rearing) present.
A score of 3: Completely flat/laying on side.

*Spontaneous activity*: Mouse activity was recorded after spending 1 min in the viewing jar (allowing some time for the animal to accommodate). Following scores were assigned.

A score of 0: none, resting
A score of 1: casual scratch, groom, slow movement
A score of 2: vigorous, rapid/dart movement
A score of 3: extremely vigorous, rapid/dart movement

*Tremor*: Following scores were assigned to the animals in the viewing jar while recording their spontaneous activity.

A score of 0: no tremor
A score of 1: mild tremor
A score of 2: severe tremor

*Ledge test*: An animal was held by its tail on the cage ledge and observed as it walked along the ledge. Following scores were assigned.

A score of 0: The animal walks along the ledge without losing its balance. No tremor apparent and the walk is highly coordinated.
A score of 1: The mouse loses its footing while walking along the ledge, but otherwise appears coordinated.
A score of 2: The mouse shows signs of tremor, loses its footing while walking along the ledge, and it appears like it struggles to walk.
A score of 3: Severe tremor is present, and the mouse falls off the ledge (or nearly so) or shakes and refuses to move at all despite encouragement.

*Hindlimb clasping*: An animal was lifted by the base of its tail and held clear from surroundings to observe the position of the hindlimbs for 30 s. Following scores were assigned.

A score of 0: The hindlimbs are consistently splayed outward, away from the abdomen.
A score of 1: One hindlimb is retracted toward the abdomen for more than 50% of the time suspended.
A score of 2: Both hindlimbs are partially retracted toward the abdomen for more than 50% of the time suspended.
A score of 3: Hindlimbs of the mouse are entirely retracted and touching the abdomen for more than 50% of the time suspended.

*Grip strength*: A mouse was lifted by the tail and allowed to grip the wire of the animal cage with front paws. The animal is then gently pulled horizontally backward until it let go the wire. Following scores were assigned.

A score of 0: active grip, effective
A score of 1: moderate grip, effective
A score of 2: slight grip, semi‐effective
A score of 3: none

All behavioral analyses were performed by researchers blinded to the genotype of the animals.

The graphs were prepared in Prism v9.0.1 (GraphPad) software.

### Tissue collection

Experimental animals were anaesthetized using Ketamine (100 mg/kg; intraperitoneal injection) and Xylazine (10 mg/kg; intraperitoneal injection) prior to transcardial perfusion with phosphate‐buffered saline (PBS), followed by 4% paraformaldehyde (PFA) solution. Dissected brain tissue was drop‐fixed in 4% PFA at 4°C for 12 h under constant agitation. Next day, the tissue was washed three times in PBS and stored in 70% ethanol. For histological studies, brains were processed in an automated tissue processor over 15 h as per the user’s guide, subsequently embedded in paraffin and sectioned at 7 μm in the sagittal plain using microtome (Leica RM2235). Sections were transferred to glass slides and dried overnight at 37°C.

### H&E staining, imaging, and analyses

Sections were deparaffinized with xylene and ethanol solutions of decreasing concentration and stained in Hematoxylin (Sigma Aldrich) for 3.5 min. The excess of Hematoxylin stain was removed by short immersion of slides in 1% HCl followed by another short immersion in 0.1% Li_2_CO_3_ solution. Slides further underwent co‐staining with eosin‐Y solution (Sigma Aldrich) for 30 s. The stained sections were dehydrated using 70, 90, and 100% ethanol for 30 s each, followed by xylene for 10 min, and finally mounted with Entellan medium (ProSciTech). Images were captured using Olympus VS120 brightfield slide scanner. Cortical layer thickness and hippocampal length and width measurements were acquired using ImageJ software (Schneider *et al*, 2012). The area of measurements is indicated by dotted lines on the respective images. The graphs were prepared in Prism v9.0.1 (GraphPad) software.

### Immunofluorescence staining, imaging, and analyses

Brain sections were deparaffinized and hydrated in a series of xylene and ethanol solutions of decreasing concentration prior to heat‐induced antigen retrieval using a citrate buffer‐based antigen unmasking solution (Abacus) for 10 min at 100°C. To prevent unspecific binding of antibodies, M.O.M. blocking reagent (Vector) was used for primary antibodies raised in mouse, and 4% horse serum (Life Technologies) for the antibodies raised in other species. Following primary antibodies were used: CC3 (1:50; ab2302), CHOP (1:100; ab11419), MBP (1:100; ab40390), Parvalbumin (1:200; ab11427), Pcp2 (1:100; sc‐49072), and Ub (1:100; ab7780), followed by incubation with an AF488 or AF594‐labeled donkey anti‐mouse, anti‐rabbit, or anti‐goat antibody (1:250; Invitrogen) and counterstained with DAPI (Sigma Aldrich). Confocal images were taken using Olympus FV3000 and Zeiss LSM710 upright microscopes as Z‐stacks and presented as a sum of the Z‐projections. The images were further exported for processing and analysis in ImageJ (Schneider *et al*, 2012). Size of the sections on which cell number and mean pixel intensity quantifications were performed is specified in the figures. The graphs were prepared in Prism v9.0.1 (GraphPad) software.

### Golgi‐Cox staining and neuron morphology analyses

Animals were transcardially perfused at P60 using PBS followed by 0.04% PFA. Brains were dissected and stained using FD Rapid GolgiStain Kit (FD Neuro Technologies, Columbia, USA) per manufacturer’s instructions. The brains were sectioned in the coronal plain at 150 μm thickness in 30% sucrose using a vibratome (Leica VT1000 S). Sections were imaged using Olympus BX63 upright microscope. Cortical pyramidal neurons from the II and III layers of the sensorimotor cortex and hippocampal pyramidal neurons from the CA1 region were selected for morphological analysis. Basal dendritic trees were reconstructed for Scholl analysis using Simple Neurite Tracer (Longair *et al*, 2011) in ImageJ software (Schneider *et al*, 2012). Clearly defined dendritic spines were quantified along 10 μm length of each neuron’s secondary shaft. The graphs were prepared in Prism v9.0.1 (GraphPad) software.

### Protein extraction and western blot analysis (murine samples and human fibroblasts)

Brain tissue was homogenized, and fibroblasts lysed in radioimmunoprecipitation assay (RIPA) buffer (150 mM NaCl, 1% Nonidet P‐40, 0.5% sodium deoxycholate, 0.1% SDS, 25 mM Tris pH 8), with protease inhibitors (Cell Signalling). Protein concentration was determined by BCA assay (Pierce). Total of 25 μg of protein lysate per sample was resolved on SDS–PAGE using NuPAGE 4–12% gels (NP0322PK), mini gel tank, and blot system (Life Technologies). Proteins were transferred onto polyvinylidene difluoride membrane at 25 V for 90 min. The membrane was incubated for 1 h in 5% skimmed milk in TBS‐T (10 mM Tris pH 8, 150 mM NaCl, 0.1% Tween 20) under constant agitation, washed twice in TBS‐T, and incubated overnight at 4°C with antibodies against βIII‐tubulin (1:10,000; ab78078), β‐actin (1:10,000; ab8224), Atf‐4 (1:1,000; ab1371), Elp1 (1:1,000; #5071), Elp2 (1:500; ab154643), Elp4 (1:500; NBP2‐16322), and Elp6 (1:500; orb53335). Next day, the membrane was washed in TBS‐T three times for 15 min and incubated under constant agitation for 1 h at room temperature with relevant horseradish peroxidase‐conjugated secondary antibodies (1:2,500; Abcam). Peroxidase activity was detected using SuperSignal West Pico chemiluminescent (Thermo Fisher Scientific) by ChemiDoc MP imaging system (Bio‐Rad Laboratories). Densitometric analysis of western blot images was performed using ImageJ software (Schneider *et al*, 2012). The graphs were prepared in Prism v9.0.1 (GraphPad) software.

### High‐performance liquid chromatography (HPLC)/mass spectrometry (MS) protein analysis

The analysis of total protein lysates from human fibroblasts was performed as previously described (Kojic *et al*, 2021). First, in‐gel destaining and trypsin digestion of proteins was performed. In short, 9 samples (*ELP4* variants, *ELP6* variants and controls; 3 technical repeats per genotype) were excised from each lane of the Coomassie blue‐stained SDS–PAGE gel and placed into separate Eppendorf tubes (10 gel pieces per each lane/sample were obtained). Destaining of these samples was performed by adding 500 µl of 100 mM ammonium bicarbonate/acetonitrile (1:1; vol/vol) buffer and incubating the samples for 30 min. The buffer was replaced with 200 µl of acetonitrile in two separate batches. Upon removal of acetonitrile, the gel pieces were covered with 200 µl of sequence‐grade trypsin of 20 ng/µl concentration in 50 mM ammonium bicarbonate pH 8 buffer (Promega Corporation) and incubated at 37°C overnight. Next day, 200 µl of 5% formic acid/acetonitrile (3:1; vol/vol) was added to the samples. The supernatant with the trypsin solution was transferred to a clean tube for each sample and dried in a vacuum centrifuge. Prior to high‐performance liquid chromatography (HPLC) and mass spectrometry (MS) analysis, 12 µl of 1% (vol/vol) TFA in water was added to each tube, followed by centrifugation for 1 min at 12,000 *g*.

The tryptic peptide extracts were analyzed by nano‐HPLC and MS on an Eksigent, Ekspert nano LC400 HPLC (SCIEX, Canada) coupled to a Triple TOF 6600 mass spectrometer (SCIEX, Canada) with a micro DuoIonSpray microflow ion source (SCIEX, Canada). 5 µl of each sample were injected onto a 5 mm × 300 µm, C18 3 µm trap column (SGE, Australia) for 6 min at 10 µl/min. This was followed by washing the trapped tryptic peptide extracts onto analytical 300 µm × 150 mm Zorbax 300SB C18 3.5 µm column (Agilent Technologies, USA) at 45°C and a 3 µl/min flow rate. For peptide elution, a linear gradient of 2–25% solvent B, 0.1% formic acid in acetonitrile, over 60 min at 3 µl/min was used, followed by 25–35% solvent B in 13 min, and then 35–80% solvent B in 2 min, then finally 80–90% solvent B for 5 min. Following each elution, the gradient was returned to 2% of 0.1% formic acid in acetonitrile for equilibration prior to injecting the next sample. The ionspray voltage was set to 5,500 V, declustering potential (DP) 80 V, curtain gas flow 25, nebuliser gas 1 (GS1) 15, heater gas (GS2) 30, interface heater at 150°C. 150 ms full‐scan TOF‐mass spectrometry (TOF‐MS) data was acquired by the spectrometer, followed by up to 50 ms full‐scan product ion data with a rolling collision energy and in an Information Dependent Acquisition (IDA) mode. The TOF‐MS data were acquired over the mass range 350–1,800 and for product ion ms/ms 100–1,500. Ions in the TOF‐MS scan that were found to exceed a 150 counts threshold and +2 to +5 charge state were set to trigger the acquisition of product ions.

Data were acquired under the same HPLC and MS experimental conditions, with the following exceptions. Data were acquired using a SWATH, product ion ms/ms all approach. The SWATH experiment was set to acquire 100 product ion spectra from m/z 350 to 1,500 per scan cycle with a product ion window was set to 6 Da and collision energy from 16 to 60 V with an energy spread of 5 V. The TOF‐MS scan acquisition time was set to 50 ms and each product ion scan to 25 ms. The data were acquired and processed using Analyst TF 1.7 software (ABSCIEX, Canada). Protein identification and library compilation were carried out using Protein Pilot 5.0.2 for database searching. Peptide sequence identification and protein grouping were conducted by merging all individual IDA acquisition file from the gel fractions using the Paragon algorithm from Protein Pilot 5.0.2 against the *Homo sapiens* Uniprot database complemented by the contaminant sequences from thegpm.org/crap/ with the following parameters: Cys alkylation was set to iodoacetamide, Trypsin was chosen as digestion enzyme, Special Factors: Gel‐based‐ID was enabled to account for potential, ID Focus: Biological modifications, Thorough ID, ProtScore (Conf) > 10%, and False discovery analysis. The processing settings were set as follows for the peak area extraction in all SWATH acquisition using the local generated ion library: Number of peptides per Protein: 6, Number of Transitions per Peptides: 6, Peptide Confidence Threshold: 99%, < 1% FDR. Modified peptides were excluded for the protein quantitation and XIC extraction window: 15 min with a mass width of 50 ppm and retention times were aligned using a set of high intense Trypsin autodigest peptides. The ion, peptide, and protein area were exported as a text file and the integrated area values for each protein that was present in different gel bands for each gel lane were summed before statistical evaluations, correlation of variation, and fold change computation. Normalization evaluations were done using the Normalyzer tool.

### Proteomics data analysis

A total number of 2,730 peptides corresponding to 660 proteins were identified by DIA/SWATH‐MS. Peptide‐level intensities were log2 transformed and normalized using the cyclic loess method. Peptides detected in less than four samples were removed and then missing values were imputed using msImpute R package (preprint: Hediyeh‐zadeh *et al*, 2020). Differential analysis of peptides was performed with limma package using linear models with empirical bayes moderation, where *P*‐values were adjusted for multiple hypothesis testing using Benjamini‐Hochberg method (Ritchie *et al*, 2015). Peptides with adjusted *P*‐values < 0.05 were considered significant. Pathway enrichment was performed on gene sets obtained from MSigDB (Liberzon *et al*, 2011) in R using fgsea package (preprint: Korotkevich *et al*, 2021) by ranking peptides using their log‐fold change. The significantly enriched pathways (adjusted *P*‐value < 0.05) were subsequently clustered and visualized using vissE R package (Bhuva, 2021).

### tRNA modification analysis

Total RNA was extracted from approximately 100 mg of brain tissue from P60 mice and homogenized with ceramic beads (Sapphire Bioscience) in a TRIzol reagent (Life Technologies) using tissue homogenizer (Bertin Technologies). Total RNA and tRNA extraction, tRNA hydrolysis to ribonucleosides, and tRNA modification analysis were performed as previously described (Kojic *et al*, 2018). HPLC coupled to MS was performed using a Luna Omega 1.6 μm, Polar‐C18 100 Å column (150 mm × 2.1 mm, Phenomenex, Australia). Mass spectrometry parameters were determined for targeted ribonucleosides using multiple injections of 0.1–1 ng of purified ncm^5^U, mcm^5^U and mcm^5^s^2^U nucleosides (a generous gift from Sebastian Leidel, University of Bern, Switzerland) and commercially obtained m^7^G (Santa Cruz Biotechnology). The obtained retention times for these nucleosides were superimposed with the previously published results (Su *et al*, 2014) and the same used to obtain retention times for m^1^A and pseudouridine (Ψ). MultiQuant‐v2.1.1 (ABSciex) software was used for peak assignment and quantification. Pseudouridine served as an internal normalization standard. The graphs were prepared in Prism v9.0.1 (GraphPad) software.

### Hippocampal slice preparation for electrophysiology

Following anesthesia by sodium pentobarbital (60–80 mg/kg; intraperitoneal injection), whole brains were dissected out of the skull and submerged in a Mg^2+^‐high and sucrose‐high artificial cerebrospinal fluid (aCSF; 87 mM NaCl, 2.5 mM KCl, 1.3 mM NaH_2_PO_4_, 7 mM MgCl_2_, 25 mM NaHCO_3_, 75 mM sucrose, 5 mM ascorbate and 0.5 mM CaCl_2_) made fresh on the day and frozen at −80°C for 30 min. The caudal end of the brain was fixed on a metal platform by cyanoacrylate glue with the ventral side supported by an agar block and submerged into ice‐cold aCSF (as above) in a chamber. Coronal hippocampal sections of 300 μm thickness were made using a vibratome (Leica VT1200 S), approximately from bregma −1.58 mm to −2.70 mm. These slices were incubated in the same aCSF at 37°C for 40 min and then transferred into a new holding chamber with a Ca^2+^‐ high and Mg^2+^‐low recording aCSF (130 mM NaCl, 26 mM NaHCO_3_, 3 mM KCl, 1 mM MgCl_2_, 2 mM CaCl_2_, 1.25 mM NaH_2_PO_4_ and 10 mM D‐glucose) at room temperature (20–22°C) for 30 min. All solutions were constantly bubbled with carbogen (95% O_2_ + 5% CO_2_) to maintain a pH of 7.4.

### Electrophysiological recordings and induction protocols

Individual hippocampal slices were transferred into a recording chamber (Kerr Scientific Instruments, NZ), held down with a platinum wire/nylon thread U‐shaped mesh (10 mm × 8 mm), and continuously superfused with recording aCSF (1–3 ml/min) at 22°C throughout the experiment. Neurons from CA3 to CA1 region were visualized with a Nikon SMZ445 stereomicroscope. Extracellular field recordings in the CA1 stratum radiatum were obtained with a monopolar recording electrode and evoked by a bipolar stimulating electrode separated by approximately 3 mm. The fEPSP was evoked by brief electrical stimuli (0.1 ms, 0.1–2 mA) at 0.1 Hz for generating an Input‐Output (I/O) curve and for baseline recordings, whereas 3 bursts of 100 Hz high‐frequency stimulation for 1 s were used for LTP induction. Apart from LTP induction, fEPSPs was recorded at 10‐second intervals throughout experiments.

An I/O curve was first generated to determine the stimulus strength used for each experiment, which was 50–60% of the stimulus strength resulting in the maximum fEPSP response. Following a 10‐min baseline recording period where the fEPSP amplitude and initial slope were considered stable, LTP was induced by three separate bursts of high‐frequency electrical stimulation (100 Hz with 100 stimuli for 1 s) at the Schaffer collaterals within 3 min. Post‐LTP induction was subsequently recorded for 30 min at 0.1 Hz with an interval of 10 s. The stimulus was delivered via a stimulus isolator (FE180, ADInstruments). Data were AC filtered and amplified (1–100 ×, Kerr Scientific Instruments S2 amplifier), digitally low‐pass filtered (2 kHz cutoff), sampled at 10 kHz (Power lab, ADInstruments), and recorded and processed using LabChart 7 software (ADInstruments). LabChart Reader (ADInstruments) was used for initial data analysis. The graphs were prepared in Prism v9.0.1 (GraphPad) software.

### Statistical analysis

Statistical analysis was performed using Prism v8.0.2 and v9.0.1 (GraphPad) software. Number of replicates, corresponding statistical tests, and statistically significant differences are indicated in figure legends. Statistically significant differences are indicated (*P ≤ 0.05; **P ≤ 0.01; ***P ≤ 0.001; ****P ≤ 0.0001; ns, not significant). Data represent mean ± SD or SEM. Differences between groups were considered significant for *P* ≤ 0.05.

## Author contributions

**Monika Gaik:** Conceptualization; Data curation; Formal analysis; Validation; Investigation; Visualization; Writing—original draft; Writing—review & editing. **Marija Kojic:** Conceptualization; Data curation; Formal analysis; Validation; Investigation; Visualization; Writing—original draft; Writing—review & editing. **Megan R Stegeman:** Data curation; Investigation; Methodology. **Tülay Öncü‐Öner:** Data curation; Formal analysis; Validation; Investigation; Visualization; Methodology; Writing—review & editing. **Anna Kościelniak:** Data curation; Investigation; Methodology. **Alun Jones:** Data curation; Investigation; Visualization; Methodology. **Ahmed Mohamed:** Data curation; Validation; Investigation; Visualization. **Pak Yan Stefanie Chau:** Data curation; Investigation; Methodology. **Sazia Sharmin:** Data curation; Investigation; Methodology. **Andrzej Chramiec‐Głąbik:** Data curation; Funding acquisition; Investigation. **Paulina Indyka:** Data curation; Investigation; Methodology. **Michal Rawski:** Data curation; Investigation; Methodology. **Anna Biela:** Data curation; Investigation; Methodology. **Dominika Dobosz:** Data curation; Investigation; Methodology. **Amanda Millar:** Data curation; Investigation; Methodology. **Vann Chau:** Data curation; Investigation; Methodology. **Aycan Ünalp:** Resources. **Michael Piper:** Data curation; Investigation; Methodology. **Mark C Bellingham:** Data curation; Investigation; Visualization; Methodology. **Evan E Eichler:** Resources; Data curation; Supervision; Funding acquisition; Investigation. **Deborah A Nickerson:** Resources; Data curation; Funding acquisition; Investigation. **Handan Güleryüz:** Resources; Visualization. **Nour El Hana Abbassi:** Data curation; Investigation; Methodology. **Konrad Jazgar:** Data curation; Investigation; Methodology. **Melissa J Davis:** Data curation; Investigation; Methodology. **Saadet Mercimek‐Andrews:** Data curation; Investigation; Methodology. **Sultan Cingöz:** Conceptualization; Resources; Data curation; Formal analysis; Supervision; Funding acquisition; Validation; Investigation; Methodology; Project administration; Writing—review & editing. **Brandon J Wainwright:** Conceptualization; Data curation; Supervision; Validation; Investigation; Methodology; Writing—original draft; Project administration; Writing—review & editing. **Sebastian Glatt:** Conceptualization; Data curation; Formal analysis; Supervision; Funding acquisition; Validation; Investigation; Visualization; Methodology; Writing—original draft; Project administration; Writing—review & editing.

## Disclosure and competing interests statement

The authors declare that they have no conflict of interest.

## Supporting information



Appendix

Expanded View Figures PDF

Source Data for Expanded View

## Data Availability

The cryo‐EM densities for mouse (EMD‐14627) and human (EMD‐14626) Elp456 have been deposited in the EMData Bank (EMDB). The mass spectrometry proteomics data have been deposited to the ProteomeXchange Consortium via the PRIDE (Perez‐Riverol *et al*, 2022) partner repository with the dataset identifier PXD032925 (http://www.ebi.ac.uk/pride/archive/projects/PXD032925). The *ELP4* and *ELP6* variants identified in the patients have been deposited for public access within ClinVar (https://www.ncbi.nlm.nih.gov/clinvar/) with accession numbers SCV002038507.1, SCV002038506 .1 and SCV002498567. All other data generated in this study are available from corresponding authors on reasonable request.

## References

[emmm202115608-bib-0001] Addis L , Ahn JW , Dobson R , Dixit A , Ogilvie CM , Pinto D , Vaags AK , Coon H , Chaste P , Wilson S (2015) Microdeletions of ELP4 are associated with language impairment, autism spectrum disorder, and mental retardation. Hum Mutat 36: 842–850 26010655 10.1002/humu.22816

[emmm202115608-bib-0002] Alizadeh N , Omran S , Birgani M , Mohammadiasl J , Hajjari M (2018) Whole exome sequencing reveals a mutation in ELP2 gene in Iranian family suffering from autosomal recessive mental retardation. J Mol Genet Med 12: 2

[emmm202115608-bib-0003] Anand KS , Dhikav V (2012) Hippocampus in health and disease: an overview. Ann Indian Acad Neurol 15: 239 23349586 10.4103/0972-2327.104323 PMC3548359

[emmm202115608-bib-0004] Anderson SL , Coli R , Daly IW , Kichula EA , Rork MJ , Volpi SA , Ekstein J , Rubin BY (2001) Familial dysautonomia is caused by mutations of the IKAP gene. Am J Hum Genet 68: 753–758 11179021 10.1086/318808 PMC1274486

[emmm202115608-bib-0005] Bento‐Abreu A , Jager G , Swinnen B , Rué L , Hendrickx S , Jones A , Staats KA , Taes I , Eykens C , Nonneman A *et al* (2018) Elongator subunit 3 (ELP3) modifies ALS through tRNA modification. Hum Mol Genet 27: 1276–1289 29415125 10.1093/hmg/ddy043 PMC6159532

[emmm202115608-bib-0006] Bertoni M , Kiefer F , Biasini M , Bordoli L , Schwede T (2017) Modeling protein quaternary structure of homo‐and hetero‐oligomers beyond binary interactions by homology. Sci Rep 7: 1–15 28874689 10.1038/s41598-017-09654-8 PMC5585393

[emmm202115608-bib-0007] Bhuva DD (2021) vissE: visualising set enrichment analysis results, R package version 122.

[emmm202115608-bib-0008] Cahill LS , Zhang MA , Ramaglia V , Whetstone H , Sabbagh MP , Yi TJ , Woo L , Przybycien TS , Moshkova M , Zhao FL *et al* (2019) Aged hind‐limb clasping experimental autoimmune encephalomyelitis models aspects of the neurodegenerative process seen in multiple sclerosis. Proc Natl Acad Sci USA 116: 22710–22720 31641069 10.1073/pnas.1915141116 PMC6842635

[emmm202115608-bib-0009] Carvill GL , Jansen S , Lacroix A , Zemel M , Mehaffey M , De Vries P , Brunner HG , Scheffer IE , De Vries BBA , Vissers LELM *et al* (2021) Genetic convergence of developmental and epileptic encephalopathies and intellectual disability. Dev Med Child Neurol 63: 1441–1447 34247411 10.1111/dmcn.14989

[emmm202115608-bib-0010] Cendelin J (2014) From mice to men: lessons from mutant ataxic mice. Cerebellum Ataxias 1: 4 26331028 10.1186/2053-8871-1-4 PMC4549131

[emmm202115608-bib-0011] Chaverra M , George L , Mergy M , Waller H , Kujawa K , Murnion C , Sharples E , Thorne J , Podgajny N , Grindeland A *et al* (2017) The familial dysautonomia disease gene IKBKAP is required in the developing and adult mouse central nervous system. Dis Model Mech 10: 605–618 28167615 10.1242/dmm.028258 PMC5451171

[emmm202115608-bib-0012] Chen Y‐T , Hims MM , Shetty RS , Mull J , Liu L , Leyne M , Slaugenhaupt SA (2009) Loss of mouse Ikbkap, a subunit of Elongator, leads to transcriptional deficits and embryonic lethality that can be rescued by human IKBKAP. Mol Cell Biol 29: 736–744 19015235 10.1128/MCB.01313-08 PMC2630687

[emmm202115608-bib-0013] Chow J , Jensen M , Amini H , Hormozdiari F , Penn O , Shifman S , Girirajan S , Hormozdiari F (2019) Dissecting the genetic basis of comorbid epilepsy phenotypes in neurodevelopmental disorders. Genome Med 11: 65 31653223 10.1186/s13073-019-0678-y PMC6815046

[emmm202115608-bib-0014] Close P , Gillard M , Ladang A , Jiang Z , Papuga J , Hawkes N , Nguyen L , Chapelle J‐P , Bouillenne F , Svejstrup J *et al* (2012) DERP6 (ELP5) and C3ORF75 (ELP6) regulate tumorigenicity and migration of melanoma cells as subunits of Elongator. J Biol Chem 287: 32535–32545 22854966 10.1074/jbc.M112.402727 PMC3463322

[emmm202115608-bib-0015] Cohen JS , Srivastava S , Farwell KD , Lu HM , Zeng W , Lu H , Chao EC , Fatemi A (2015) ELP2 is a novel gene implicated in neurodevelopmental disabilities. Am J Med Genet A 167: 1391–1395 25847581 10.1002/ajmg.a.36935

[emmm202115608-bib-0016] Creppe C , Malinouskaya L , Volvert M‐L , Gillard M , Close P , Malaise O , Laguesse S , Cornez I , Rahmouni S , Ormenese S *et al* (2009) Elongator controls the migration and differentiation of cortical neurons through acetylation of α‐tubulin. Cell 136: 551–564 19185337 10.1016/j.cell.2008.11.043

[emmm202115608-bib-0018] Dauden MI , Jaciuk M , Weis F , Lin T‐Y , Kleindienst C , Abbassi NEH , Khatter H , Krutyhołowa R , Breunig KD , Kosinski J *et al* (2019) Molecular basis of tRNA recognition by the Elongator complex. Sci Adv 5: eaaw2326 31309145 10.1126/sciadv.aaw2326 PMC6620098

[emmm202115608-bib-0019] Dauden MI , Kosinski J , Kolaj‐Robin O , Desfosses A , Ori A , Faux C , Hoffmann NA , Onuma OF , Breunig KD , Beck M *et al* (2017) Architecture of the yeast Elongator complex. EMBO Rep 18: 264–279 27974378 10.15252/embr.201643353 PMC5286394

[emmm202115608-bib-0020] Duan Y , Leng X , Liu C , Qi X , Zhang L , Tan W , Zhang X , Wang Y (2021) The correlation of ELP4‐PAX6 with rolandic spike sources in idiopathic rolandic epilepsy syndromes. Front Neurol 12: 643964 33897599 10.3389/fneur.2021.643964 PMC8064626

[emmm202115608-bib-0021] Esberg A , Huang B , Johansson MJ , Byström AS (2006) Elevated levels of two tRNA species bypass the requirement for elongator complex in transcription and exocytosis. Mol Cell 24: 139–148 17018299 10.1016/j.molcel.2006.07.031

[emmm202115608-bib-0022] Glatt S , Létoquart J , Faux C , Taylor NM , Séraphin B , Müller CW (2012) The Elongator subcomplex Elp456 is a hexameric RecA‐like ATPase. Nat Struct Mol Biol 19: 314–320 22343726 10.1038/nsmb.2234

[emmm202115608-bib-0023] Goddard TD , Huang CC , Meng EC , Pettersen EF , Couch GS , Morris JH , Ferrin TE (2018) UCSF ChimeraX: meeting modern challenges in visualization and analysis. Protein Sci 27: 14–25 28710774 10.1002/pro.3235 PMC5734306

[emmm202115608-bib-0024] Goffena J , Lefcort F , Zhang Y , Lehrmann E , Chaverra M , Felig J , Walters J , Buksch R , Becker KG , George L (2018) Elongator and codon bias regulate protein levels in mammalian peripheral neurons. Nat Commun 9: 889 29497044 10.1038/s41467-018-03221-z PMC5832791

[emmm202115608-bib-0025] Gruart A , Muñoz MD , Delgado‐García JM (2006) Involvement of the CA3–CA1 synapse in the acquisition of associative learning in behaving mice. J Neurosci 26: 1077–1087 16436593 10.1523/JNEUROSCI.2834-05.2006 PMC6674570

[emmm202115608-bib-0026] Guyenet SJ , Furrer SA , Damian VM , Baughan TD , La Spada AR , Garden GA (2010) A simple composite phenotype scoring system for evaluating mouse models of Cerebellar Ataxia. J Vis Exp e1787 10.3791/1787 PMC3121238 20495529

[emmm202115608-bib-0027] Hawer H , Hammermeister A , Ravichandran KE , Glatt S , Schaffrath R , Klassen R (2018) Roles of Elongator dependent tRNA modification pathways in neurodegeneration and cancer. Genes 10: 19 10.3390/genes10010019 PMC6356722 30597914

[emmm202115608-bib-0028] Hediyeh‐zadeh S , Webb AI , Davis MJ (2020) MSImpute: imputation of label‐free mass spectrometry peptides by low‐rank approximation. bioRxiv 10.1101/2020.08.12.248963 [PREPRINT]

[emmm202115608-bib-0029] Hormozdiari F , Penn O , Borenstein E , Eichler EE (2015) The discovery of integrated gene networks for autism and related disorders. Genome Res 25: 142–154 25378250 10.1101/gr.178855.114 PMC4317170

[emmm202115608-bib-0030] Huang B , Johansson MJ , Byström AS (2005) An early step in wobble uridine tRNA modification requires the Elongator complex. RNA 11: 424–436 15769872 10.1261/rna.7247705 PMC1370732

[emmm202115608-bib-0031] Jiang X , Mu D , Manabat C , Koshy AA , Christen S , Täuber MG , Vexler ZS , Ferriero DM (2004) Differential vulnerability of immature murine neurons to oxygen‐glucose deprivation. Exp Neurol 190: 224–232 15473995 10.1016/j.expneurol.2004.07.010

[emmm202115608-bib-0032] Kagias K , Nehammer C , Pocock R (2012) Neuronal responses to physiological stress. Front Genet 3: 222 23112806 10.3389/fgene.2012.00222 PMC3481051

[emmm202115608-bib-0033] Karlsborn T , Tükenmez H , Chen C , Byström AS (2014) Familial dysautonomia (FD) patients have reduced levels of the modified wobble nucleoside mcm^5^s^2^U in tRNA. Biochem Biophys Res Comm 454: 441–445 25450681 10.1016/j.bbrc.2014.10.116

[emmm202115608-bib-0034] Kirkcaldie MT (2012) Neocortex. In The mouse nervous system, Watson C , Paxinos G , Puelles L (eds), pp 52–111. San Diego, CA: Elsevier

[emmm202115608-bib-0035] Kojic M , Gaik M , Kiska B , Salerno‐Kochan A , Hunt S , Tedoldi A , Mureev S , Jones A , Whittle B , Genovesi LA *et al* (2018) Elongator mutation in mice induces neurodegeneration and ataxia‐like behavior. Nat Commun 9: 3195 30097576 10.1038/s41467-018-05765-6 PMC6086839

[emmm202115608-bib-0036] Kojic M , Gawda T , Gaik M , Begg A , Salerno‐Kochan A , Kurniawan ND , Jones A , Drożdżyk K , Kościelniak A , Chramiec‐Głąbik A *et al* (2021) Elp2 mutations perturb the epitranscriptome and lead to a complex neurodevelopmental phenotype. Nat Commun 12: 2678 33976153 10.1038/s41467-021-22888-5 PMC8113450

[emmm202115608-bib-0037] Kojic M , Wainwright B (2016) The many faces of Elongator in neurodevelopment and disease. Front Mol Neurosci 9: 115 27847465 10.3389/fnmol.2016.00115 PMC5088202

[emmm202115608-bib-0039] Korotkevich G , Sukhov V , Budin N , Shpak B , Artyomov MN , Sergushichev A (2021) Fast gene set enrichment analysis. bioRxiv 10.1101/060012 [PREPRINT]

[emmm202115608-bib-0040] Kwee LC , Liu Y , Haynes C , Gibson JR , Stone A , Schichman SA , Kamel F , Nelson LM , Topol B , Van Den Eeden SK *et al* (2012) A high‐density genome‐wide association screen of sporadic ALS in US veterans. PLoS One 7: e32768 22470424 10.1371/journal.pone.0032768 PMC3314660

[emmm202115608-bib-0041] Laguesse S , Creppe C , Nedialkova D , Prévot P‐P , Borgs L , Huysseune S , Franco B , Duysens G , Krusy N , Lee G *et al* (2015) A dynamic unfolded protein response contributes to the control of cortical neurogenesis. Dev Cell 35: 553–567 26651292 10.1016/j.devcel.2015.11.005

[emmm202115608-bib-0042] Lalonde R , Strazielle C (2011) Brain regions and genes affecting limb‐clasping responses. Brain Res Rev 67: 252–259 21356243 10.1016/j.brainresrev.2011.02.005

[emmm202115608-bib-0043] Liberzon A , Subramanian A , Pinchback R , Thorvaldsdóttir H , Tamayo P , Mesirov JP (2011) Molecular signatures database (MSigDB) 3.0. Bioinformatics 27: 1739–1740 21546393 10.1093/bioinformatics/btr260 PMC3106198

[emmm202115608-bib-0044] Lin T‐Y , Abbassi NEH , Zakrzewski K , Chramiec‐Głąbik A , Jemioła‐Rzemińska M , Różycki J , Glatt S (2019) The Elongator subunit Elp3 is a non‐canonical tRNA acetyltransferase. Nat Commun 10: 1–12 30733442 10.1038/s41467-019-08579-2 PMC6367351

[emmm202115608-bib-0045] Longair MH , Baker DA , Armstrong JD (2011) Simple Neurite Tracer: open source software for reconstruction, visualization and analysis of neuronal processes. Bioinformatics 27: 2453–2454 21727141 10.1093/bioinformatics/btr390

[emmm202115608-bib-0046] Lucas EK , Reid CS , McMeekin LJ , Dougherty SE , Floyd CL , Cowell RM (2015) Cerebellar transcriptional alterations with Purkinje cell dysfunction and loss in mice lacking PGC‐1α. Front Cell Neurosci 8: 441 25610371 10.3389/fncel.2014.00441 PMC4285109

[emmm202115608-bib-0047] Najmabadi H , Hu H , Garshasbi M , Zemojtel T , Abedini SS , Chen W , Hosseini M , Behjati F , Haas S , Jamali P *et al* (2011) Deep sequencing reveals 50 novel genes for recessive cognitive disorders. Nature 478: 57–63 21937992 10.1038/nature10423

[emmm202115608-bib-0048] Nedialkova DD , Leidel SA (2015) Optimization of codon translation rates via tRNA modifications maintains proteome integrity. Cell 161: 1606–1618 26052047 10.1016/j.cell.2015.05.022 PMC4503807

[emmm202115608-bib-0049] Panjwani N , Wilson MD , Addis L , Crosbie J , Wirrell E , Auvin S , Caraballo RH , Kinali M , McCormick D , Oren C (2016) A microRNA‐328 binding site in PAX6 is associated with centrotemporal spikes of rolandic epilepsy. Ann Clin Transl Neurol 3: 512–522 27386500 10.1002/acn3.320 PMC4931716

[emmm202115608-bib-0050] Parenti I , Rabaneda LG , Schoen H , Novarino G (2020) Neurodevelopmental disorders: from genetics to functional pathways. Trends Neurosci 43: 608–621 32507511 10.1016/j.tins.2020.05.004

[emmm202115608-bib-0051] Peng J , Zhou Y , Wang K (2021) Multiplex gene and phenotype network to characterize shared genetic pathways of epilepsy and autism. Sci Rep 11: 952 33441621 10.1038/s41598-020-78654-y PMC7806931

[emmm202115608-bib-0052] Perez‐Riverol Y , Bai J , Bandla C , García‐Seisdedos D , Hewapathirana S , Kamatchinathan S , Kundu D , Prakash A , Frericks‐Zipper A , Eisenacher M *et al* (2022) The PRIDE database resources in 2022: a hub for mass spectrometry‐based proteomics evidences. Nucleic Acids Res 50: D543–D552 34723319 10.1093/nar/gkab1038 PMC8728295

[emmm202115608-bib-0053] Pettersen EF , Goddard TD , Huang CC , Meng EC , Couch GS , Croll TI , Morris JH , Ferrin TE (2021) UCSF ChimeraX: structure visualization for researchers, educators, and developers. Protein Sci 30: 70–82 32881101 10.1002/pro.3943 PMC7737788

[emmm202115608-bib-0054] Reinthaler EM , Lal D , Jurkowski W , Feucht M , Steinböck H , Gruber‐Sedlmayr U , Ronen GM , Geldner J , Haberlandt E , Neophytou B (2014) Analysis of ELP4, SRPX2, and interacting genes in typical and atypical rolandic epilepsy. Epilepsia 55: e89–e93 24995671 10.1111/epi.12712

[emmm202115608-bib-0055] Ritchie ME , Phipson B , Wu D , Hu Y , Law CW , Shi W , Smyth GK (2015) limma powers differential expression analyses for RNA‐sequencing and microarray studies. Nucleic Acids Res 43: e47 25605792 10.1093/nar/gkv007 PMC4402510

[emmm202115608-bib-0056] Rogers DC , Fisher E , Brown S , Peters J , Hunter A , Martin J (1997) Behavioral and functional analysis of mouse phenotype: SHIRPA, a proposed protocol for comprehensive phenotype assessment. Mamm Genome 8: 711–713 9321461 10.1007/s003359900551

[emmm202115608-bib-0057] Schäck MA , Jablonski KP , Gräf S , Klassen R , Schaffrath R , Kellner S , Hammann C (2020) Eukaryotic life without tQCUG: the role of Elongator‐dependent tRNA modifications in Dictyostelium discoideum. Nucleic Acids Res 48: 7899–7913 32609816 10.1093/nar/gkaa560 PMC7430636

[emmm202115608-bib-0058] Schneider CA , Rasband WS , Eliceiri KW (2012) NIH Image to ImageJ: 25 years of image analysis. Nat Methods 9: 671–675 22930834 10.1038/nmeth.2089 PMC5554542

[emmm202115608-bib-0059] Schrödinger L (2018) The PyMOL molecular graphics system, Version 1.8. 2015.

[emmm202115608-bib-0060] Shafik AM , Allen EG , Jin P (2021) The emerging neuroepitranscriptome. In Epitranscriptomics, Jurga S , Barciszewski J (eds), pp 1–22. Cham: Springer

[emmm202115608-bib-0061] Shetty PK , Galeffi F , Turner DA (2012) Cellular links between neuronal activity and energy homeostasis. Front Pharmacol 3: 43 22470340 10.3389/fphar.2012.00043 PMC3308331

[emmm202115608-bib-0062] Simpson CL , Lemmens R , Miskiewicz K , Broom WJ , Hansen VK , van Vught PWJ , Landers JE , Sapp P , Van Den Bosch L , Knight J *et al* (2009) Variants of the Elongator protein 3 (ELP3) gene are associated with motor neuron degeneration. Hum Mol Genet 18: 472–481 18996918 10.1093/hmg/ddn375 PMC2638803

[emmm202115608-bib-0063] Slaugenhaupt SA , Blumenfeld A , Gill SP , Leyne M , Mull J , Cuajungco MP , Liebert CB , Chadwick B , Idelson M , Reznik L (2001) Tissue‐specific expression of a splicing mutation in the IKBKAP gene causes familial dysautonomia. Am J Hum Genet 68: 598–605 11179008 10.1086/318810 PMC1274473

[emmm202115608-bib-0064] Strug LJ , Clarke T , Chiang T , Chien M , Baskurt Z , Li W , Dorfman R , Bali B , Wirrell E , Kugler SL *et al* (2009) Centrotemporal sharp wave EEG trait in rolandic epilepsy maps to Elongator Protein Complex 4 (ELP4). Eur J Hum Genet 17: 1171–1181 19172991 10.1038/ejhg.2008.267 PMC2729813

[emmm202115608-bib-0065] Su D , Chan CT , Gu C , Lim KS , Chionh YH , McBee ME , Russell BS , Babu IR , Begley TJ , Dedon PC (2014) Quantitative analysis of ribonucleoside modifications in tRNA by HPLC‐coupled mass spectrometry. Nat Protoc 9: 828–841 24625781 10.1038/nprot.2014.047 PMC4313537

[emmm202115608-bib-0066] Tielens S , Huysseune S , Godin JD , Chariot A , Malgrange B , Nguyen L (2016) Elongator controls cortical interneuron migration by regulating actomyosin dynamics. Cell Res 26: 1131–1148 27670698 10.1038/cr.2016.112 PMC5113307

[emmm202115608-bib-0067] Till SM , Wijetunge LS , Seidel VG , Harlow E , Wright AK , Bagni C , Contractor A , Gillingwater TH , Kind PC (2012) Altered maturation of the primary somatosensory cortex in a mouse model of fragile X syndrome. Hum Mol Genet 21: 2143–2156 22328088 10.1093/hmg/dds030

[emmm202115608-bib-0068] Toral‐Lopez J , Huerta LMG , Messina‐Baas O , Cuevas‐Covarrubias SA (2020) Submicroscopic 11p13 deletion including the Elongator acetyltransferase complex subunit 4 gene in a girl with language failure, intellectual disability and congenital malformations: a case report. World J Clin Cases 8: 5296–5303 33269262 10.12998/wjcc.v8.i21.5296 PMC7674752

[emmm202115608-bib-0069] Verma V , Paul A , Amrapali Vishwanath A , Vaidya B , Clement JP (2019) Understanding intellectual disability and autism spectrum disorders from common mouse models: synapses to behaviour. Open Biol 9: 180265 31185809 10.1098/rsob.180265 PMC6597757

[emmm202115608-bib-0070] Weissmann F , Petzold G , VanderLinden R , Huis in 't Veld PJ , Brown NG , Lampert F , Westermann S , Stark H , Schulman BA , Peters J‐M (2016) biGBac enables rapid gene assembly for the expression of large multisubunit protein complexes. Proc Natl Acad Sci USA 113: E2564–E2569 27114506 10.1073/pnas.1604935113 PMC4868461

[emmm202115608-bib-0071] Whitlock JR , Heynen AJ , Shuler MG , Bear MF (2006) Learning induces long‐term potentiation in the Hippocampus. Science 313: 1093–1097 16931756 10.1126/science.1128134

