# Supplementary material for: Functional divergence of the two Elongator subcomplexes during neurodevelopment
Source: EMBO Mol Med. 2022 Jun 13;14(7):e15608. doi: 10.15252/emmm.202115608 (PMC9260213; doi:10.15252/emmm.202115608)
Supplement: Supplementary file 1 — Appendix [file EMMM-14-e15608-s002.pdf]

Appendix

Functional divergence of the two Elongator subcomplexes during neurodevelopment

Monika Gaik[1,#], Marija Kojic[2,#], Megan R. Stegeman[2], Tülay Öncü Öner[3], Anna Kościelniak[1], Alun Jones[4], Ahmed Mohamed[5,6,7], Pak Yan Stefanie Chau[8], Sazia Sharmin[8], Andrzej Chramiec-Głąbik[1], Paulina Indyka[1,9], Michał Rawski[1], Anna Biela[1], Dominika Dobosz[1], Amanda Millar[2], Vann Chau[10], Aycan Ünalp[11], Michael Piper[8], Mark C. Bellingham[8], Evan E. Eichler[12,13], Deborah A. Nickerson[12], Handan Güleryüz[14], Nour El Hana Abbassi[1,15], Konrad Jazgar[1], Melissa J. Davis[2,5,7,16], Saadet Mercimek-Andrews[17,18], Sultan Cingöz[3,12,*], Brandon J. Wainwright[2,*] and Sebastian Glatt[1,*]

Table of content

Appendix Figure S1. Cryo-EM reconstruction of hElp456 and mElp456 complexes

Appendix Figure S2. Analyses of patient-derived fibroblasts

Appendix Figure S3. No gross morphological abnormalities in forebrain structures and extensive Purkinje neuron degeneration in *Elp6L118W* mice

Appendix Figure S4. No interneuron and myelin defects observed in the forebrain structures of the *Elp6L118W* mice

Appendix Figure S5. Protein misfolding and ER-stress-induced apoptosis of Purkinje neurons in *Elp6L118W* mice

Appendix Figure S6. Expression of Elongator subunits in the brain tissue of *Elp6L118W* and control mice

Appendix Table S1. Nucleotide sequences used in the study

Appendix Table S2. Cryo-EM data collection and processing

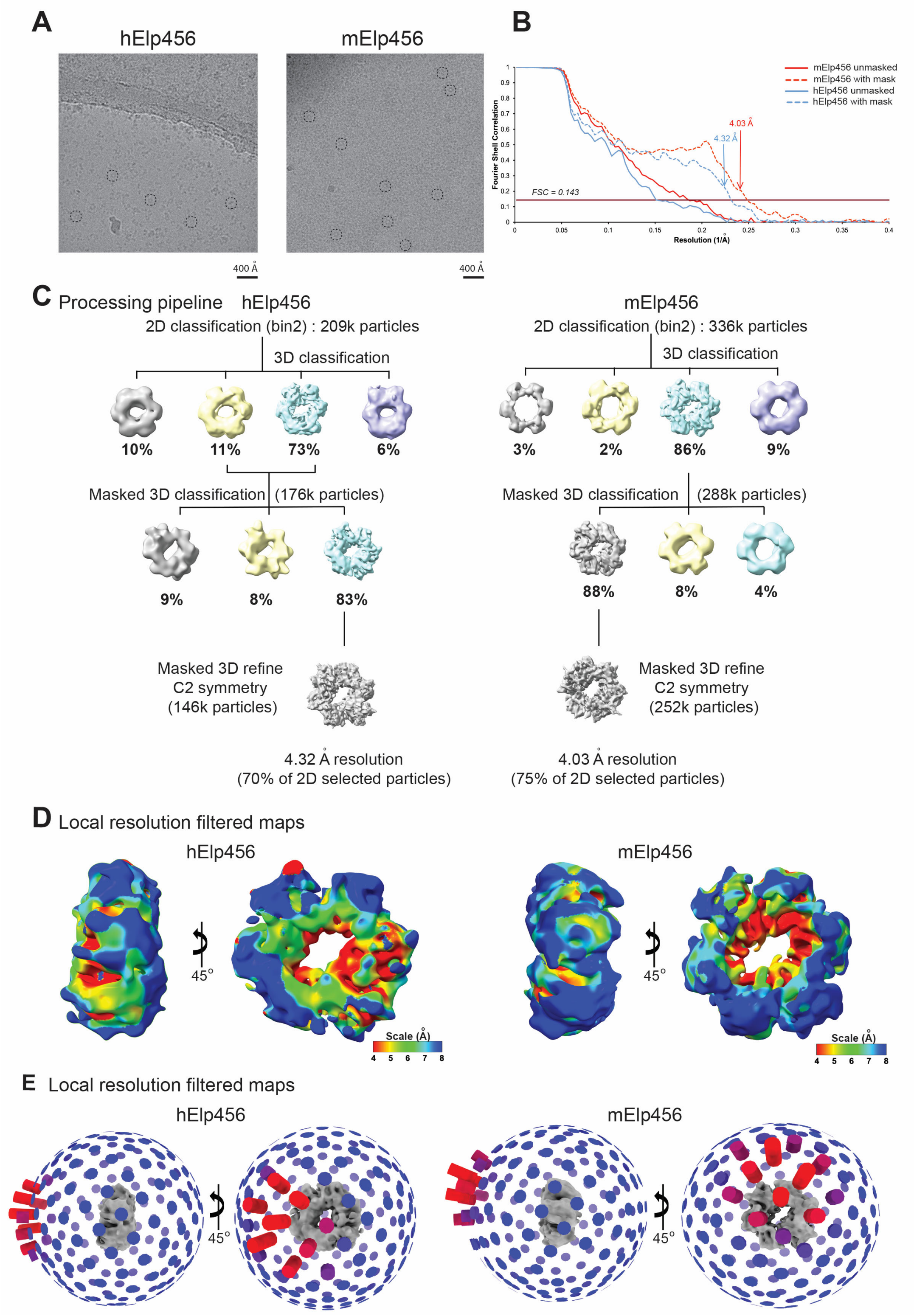

Appendix Figure S1. Cryo-EM reconstruction of hElp456 and mElp456 complexes

A. Representative cryo-EM fields of Elp456 complexes. Particles are highlighted. Scale bar: 400 Å.

B. Fourier Shell Correlation (FSC) curves for Elp456 reconstructions. The grey line indicates FSC = 0.143 and the individual resolution cutoffs are labeled.

C. The dataset processing pipelines.

D. The resolution range of Elp456 maps are displayed on the local resolution filtered maps.

E. Euler plots for the angular distribution of Elp456 in two different views for human and mouse maps.

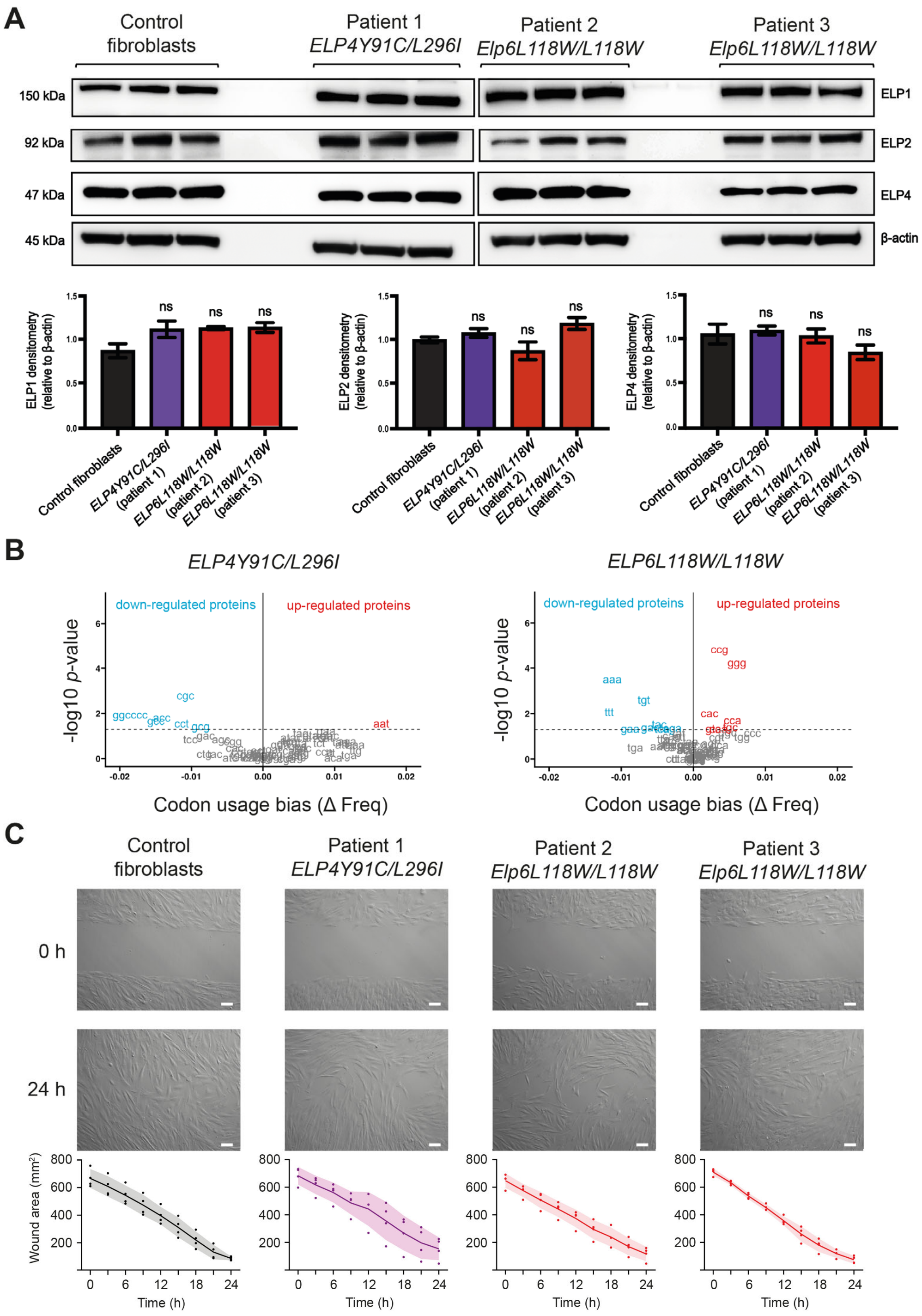

Appendix Figure S2. Analyses of patient-derived fibroblasts

A. Western blot analysis of ELP1, ELP2 and ELP4 expression with β-actin used as internal normalization standard in the human fibroblast lysates obtained from the patients with *ELP4* and *ELP6* variants and commercially available cells used as a control. Protein size is indicated on left. The protein expression is quantified and normalized with β-actin (bottom panel). $n = 3$ technical repeats. Statistical analysis: one-way ANOVA ($\alpha = 0.05$) with a Dunnett's post-hoc test. Statistically significant differences are indicated (ns - not significant). Data represent mean ± SEM.

B. Codon usage of mRNAs from mis-regulated proteins in the respective patient-derived fibroblasts. Statistical analysis: Wilcoxon test. Statistically significant differences are indicated in the image. Source data are provided as a Supplementary data file.

C. Wound healing assays using patient-derived fibroblasts and control fibroblasts. Representative images of the respective cell lines at the start (0 h) and end (24 h) of the analyses. Quantification of the unoccupied area using ImageJ; Error bars represent SD values and individual data points are shown; $n = 4$ technical repeats.

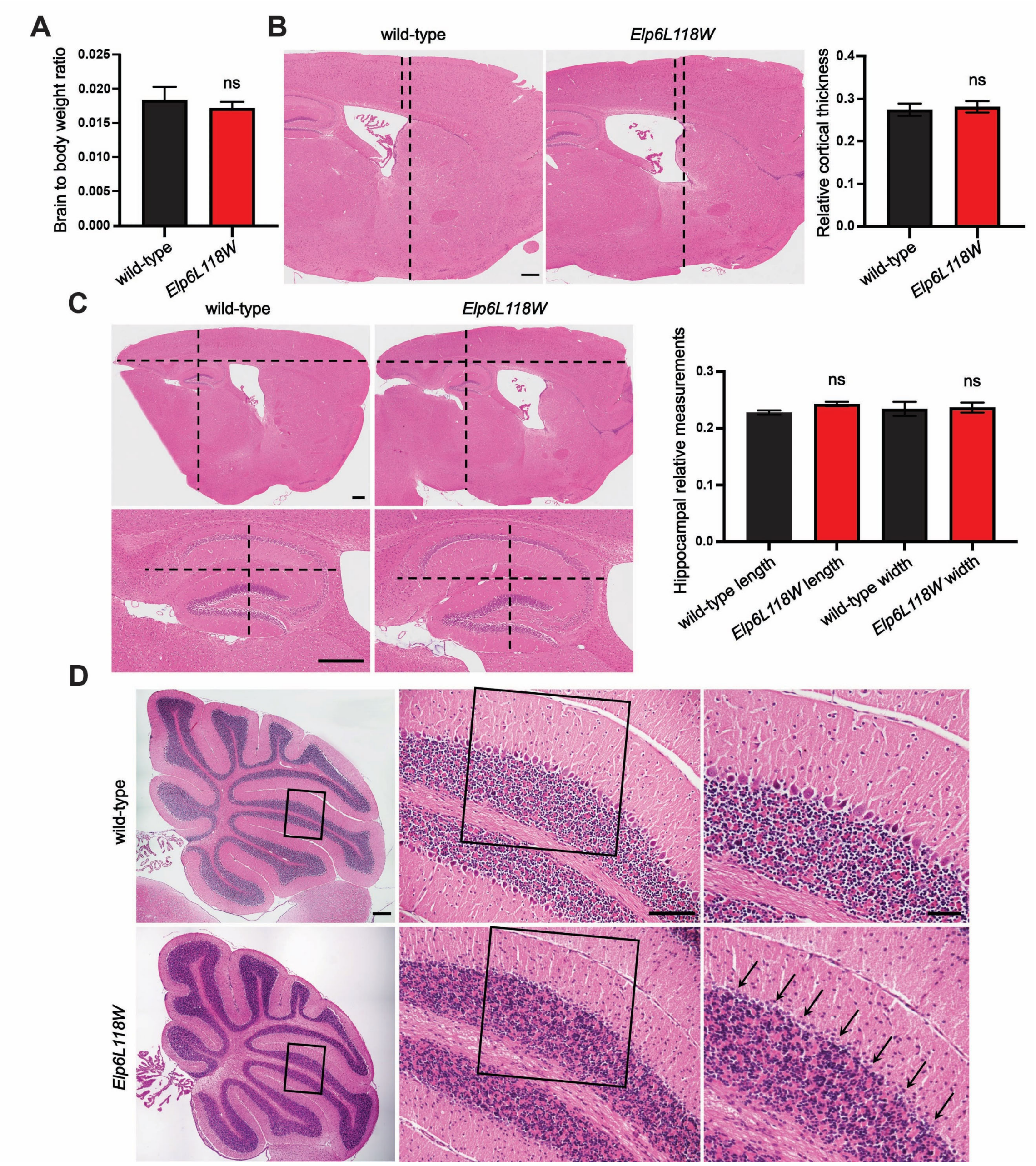

Appendix Figure S3. No gross morphological abnormalities in forebrain structures and extensive Purkinje neuron degeneration in *Elp6L118W* mice

A. Brain weight relative to body weight of wild-type and *Elp6L118W* mice at postnatal day (P) 60. $n = 6$ per genotype.

B. H&E-stained sagittal brain sections of P60 wild-type and mutant mice. Representative images and quantification of the cerebral cortex thickness relative to the whole-brain thickness. Areas of measurements are indicated by dashed lines. $n = 3$ sections per animal and 3 animals per genotype. Scale bar: 300 µm.

C. H&E-stained sagittal brain sections of P60 wild-type and mutant mice. Representative images and quantification of the hippocampal length and width relative to the whole brain length and width are shown. Areas of measurements are indicated by dashed lines. $n = 3$ sections per animal and 3 animals per genotype. Scale bar: 300 µm.

D. H&E-stained sagittal cerebellar sections of P60 wild-type and mutant mice. Rectangles represent magnified areas to emphasise depletion of Purkinje neurons from the cerebella of the *Elp6* mutant mice (indicated by arrows). $n = 3$ sections per animal and 3 animals per genotype. Scale bars: left panel 500 µm, middle panel 100 µm, right panel 50 µm.

Data information: Statistical analysis: unpaired two-tailed t-test ($\alpha = 0.05$) with Welch's correction. ns - not significant. Data represent mean ± SEM.

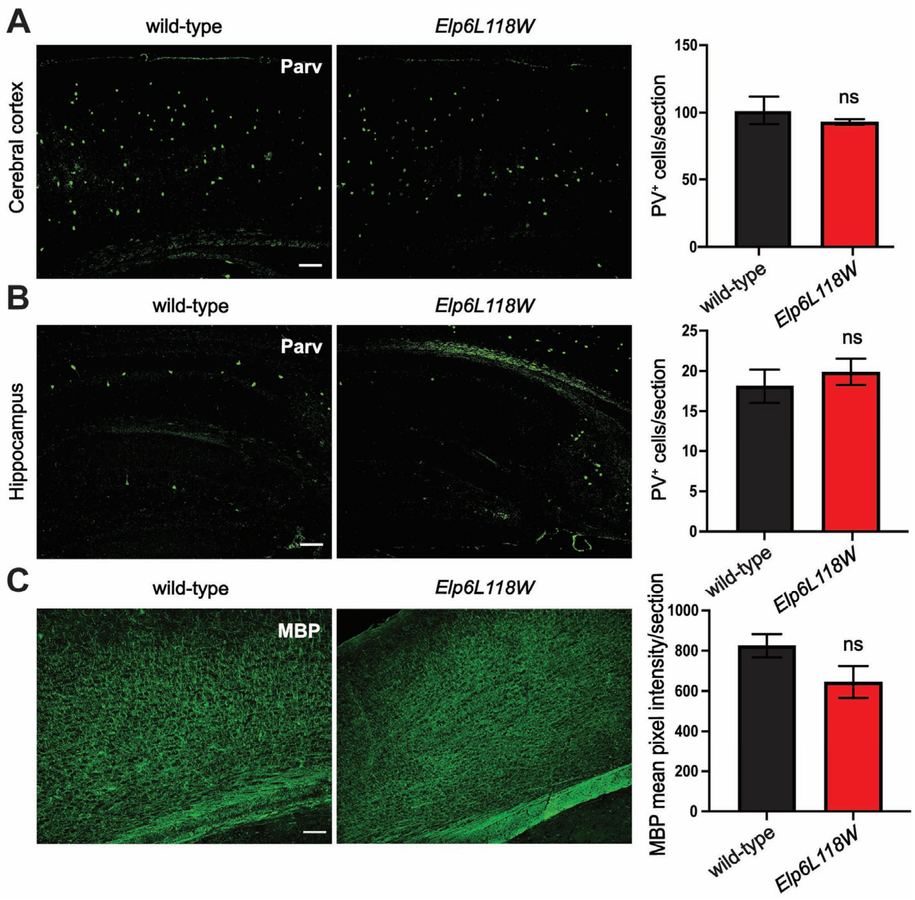

Appendix Figure S4. No interneuron and myelin defects observed in the forebrain structures of the *Elp6L118W* mice

A. Representative images and quantification of immunofluorescence-labelled sagittal brain sections of cortical parvalbumin (Parv)-expressing interneurons in adult (2-months-old) wild-type and *Elp6L118W* mice. n = 3 sections per animal and 3 animals per genotype. Scale bar: 300 µm.

B. Representative images and quantification of hippocampal parvalbumin (Parv)-expressing interneurons in adult *Elp6* mutant and control mice. n = 3 sections per animal and 3 animals per genotype. Scale bar: 300 µm.

C. Myelin basic protein (MBP)-immunostaining of sagittal cortical sections of adult wild-type and mutant mice. Representative images are shown. n = 3 sections per animal and 3 animals per genotype. Scale bar: 300 µm.

Data information: Statistical analysis: unpaired two-tailed t-test (α = 0.05) with Welch's correction. ns - not significant. Data represent mean ± SEM.

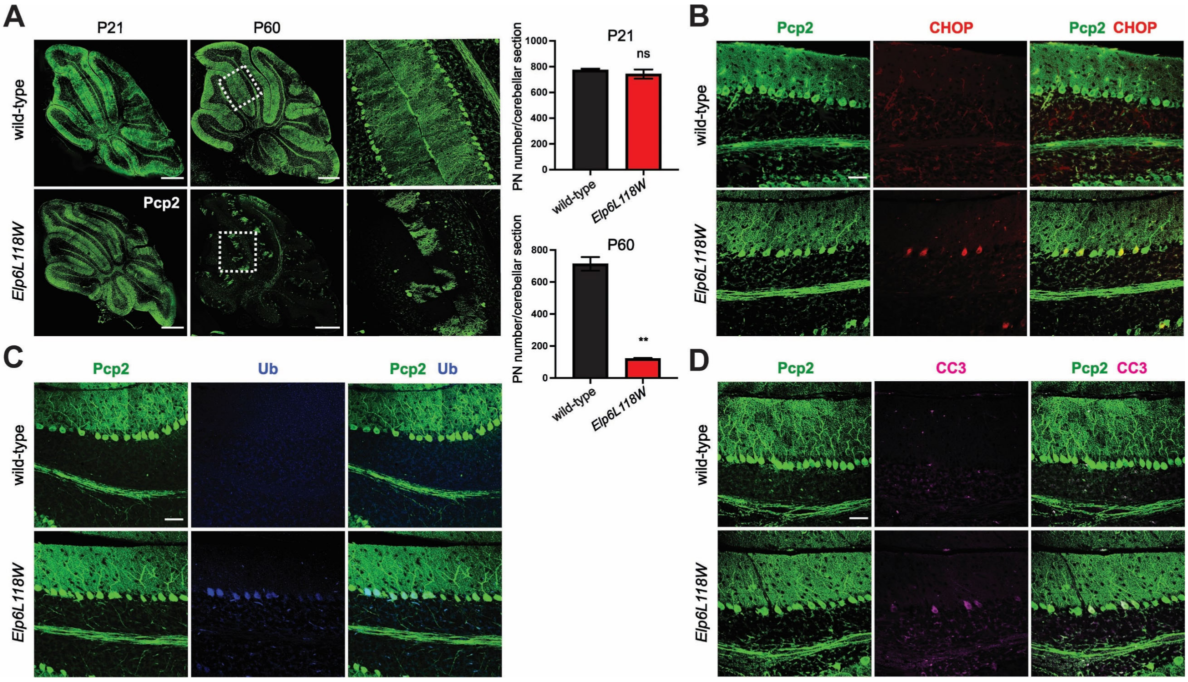

Appendix Figure S5. Protein misfolding and ER-stress-induced apoptosis of Purkinje neurons in *Elp6L118W* mice

A. Purkinje cell protein 2 (Pcp2) immunolabelling and Purkinje neuron (PN) quantification in *Elp6L118W* and wild-type mice cerebella (sagittal sections) at postnatal day (P) 21 and P60. Rectangles represent magnified areas. $n = 3$ sections per animal and 3 animals per genotype. Scale bars: left and middle panels 500 µm, right panel 100 µm.

B. Immunofluorescence staining of P21 cerebellar sections for expression of ER stress marker CHOP in PNs (Pcp2-labelled) of *Elp6* mutant and littermate control animals. $n = 3$ sections per animal and 3 animals per genotype. Scale bar: 100 µm.

C. Immunofluorescence staining of P21 cerebellar sections for expression of ubiquitin (Ub). $n = 3$ sections per animal and 3 animals per genotype. Scale bar: 100 µm.

D. Immunofluorescence staining of P21 cerebellar sections for expression of cleaved caspase 3 (CC3). $n = 3$ sections per animal and 3 animals per genotype. Scale bar: 100 µm.

Data information: Statistical analysis: unpaired two-tailed t-test ($\alpha = 0.05$) with Welch's correction. Statistically significant differences are indicated (**$p \leq 0.01$; ns - not significant). Data represent mean ± SEM.

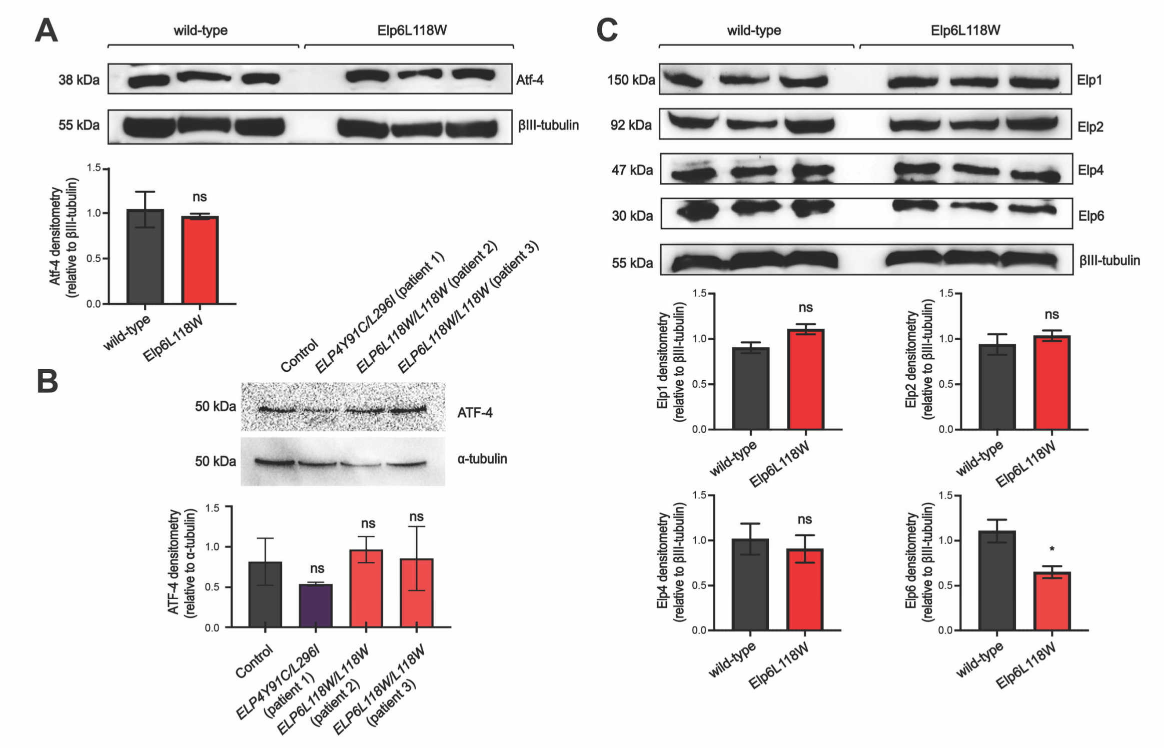

Appendix Figure S6. Expression of Elongator subunits in the brain tissue of *Elp6L118W* and control mice

A. Western blot analysis of mouse Atf-4 expression with βIII-tubulin being used as internal normalization standard in the brain lysates of 2-months-old wild-type and *Elp6* mutant animals. Protein size is indicated on left. Quantification of expression levels after normalization with βIII-tubulin is shown (bottom panel). $n = 3$ animals per genotype.

B. Western blot analysis of human ATF-4 expression with α-tubulin being used as internal normalization standard in the patient-derived fibroblasts. Protein size is indicated on left. Quantification of expression levels after normalization with α-tubulin is shown (bottom panel). $n = 3$ technical repeats.

C. Western blot analysis of Elp1, Elp2, Elp4 and Elp6 expression with βIII-tubulin being used as internal normalization standard in the brain lysates of 2-months-old wild-type and *Elp6* mutant animals. Protein size is indicated on left. The protein expression levels are quantified and normalized with βIII-tubulin (bottom panel). $n = 3$ animals per genotype.

Data information: Statistical analysis: unpaired two-tailed *t*-test ($\alpha = 0.05$) with Welch's correction. Statistically significant differences are indicated (*$p \leq 0.05$; ns - not significant). Data represent mean ± SEM.

Appendix Table S1. Nucleotide sequences used in the study

Name	Sequence 5′ - 3′
**Genotyping**	
**Mouse genotyping primer F**	TTCTCCAGCCTCCCTTGGTG
**Mouse genotyping primer R**	CTTCAGTTCGCAGCACACCG
**VIC-labelled wild-type sequence (mouse genotyping)**	GAAAGCTTTGGTGACTTTTCCCTATGTTTCTAGGGAGGCTGGTACAGGAAACCTGCAGTCACTGTATACGTTTATTCAGGACACCCTGAAGCCTGCGGACAGTGAGG
**FAM-labelled *Elp6L118W* sequence (mouse genotyping)**	GAAAGCTTTGGTGACTTTTCCCTATGTTTCTAGGGAGGCTGGTACAGGAAACCTGCAGTCATGGTATACGTTTATTCAGGACACCCTGAAGCCTGCGGACAGTGAGG
**Cloning & mutagenesis**	
**mElp4 F**	CGTACGCATATGGCTGCAGCTGACACTTG
**mElp4 R**	CTTGTAGCGGCCGCTTAGAAGTCCAAGTGCTTCTTTC
**mElp5 F**	CGTACGCATATGCTGGACTCGTTGTTGGC
**mElp5 R**	CTTGTAGCGGCCGCTTAAATATCTAGGTCATCATCTGG
**mElp6 F**	CGTACGCATATGTTTCCGGAACTGAACAATTTG
**mElp6 R**	CTTGTAGGATCCCAAAACGGCAGGAGACATTC
**mElp4_Y90C F**	CCCTTCTCCTGATCGAGGAGGATAAGTGTAACATTTATTCTCCGCTGCTGTTC
**mElp4_Y90C R**	GTACTTGAACAGCAGCGGAGAATAAATGTTACACTTATCCTCCTCGATCAGGAG
**mElp4_L294I F**	CTTCGCGGTCTGCTGCGGTCTTCAATCTCCGCTTGCATTATTACAATGCCG
**mElp4_L294I R**	GTGCCGGCATTGTAATAATGCAAGCGGAGATTGAAGACCGCAGCAGACCGCG
**mElp6_L118W F**	CTGGGAACCTCCAATCTTGGTACACATTTATTCAGGATAC
**mElp6_L118W R**	GTATCCTGAATAAATGTGTACCAAGATTGGAGGTTCCCAG
**mElp6_L126Q F**	CAATCTCTTTACACATTTATTCAGGATACGCAGAAACCCGCTGATTCTGAG
**mElp6_L126Q R**	CTCAGAATCAGCGGGTTTCTGCGTATCCTGAATAAATGTGTAAAGAGATTG
**hElp4_ F**	AACGCTCTATGGTCTAAAGATTTAAATCGACCTACTCCGGAATATTAATAGATC
**hElp4_ R**	AAACGTGCAATAGTATCCAGTTTATTTAAATGGTGTAGCGTCGTAAGCTAATACG
**hElp5_ F**	AAACTGGATACTATTGCACGTTTAAATCGACCTACTCCGGAATATTAATAGATC
**hElp5_ R**	AAACATCAGGCATCATTAGGTTTATTTAAATGGTGTAGCGTCGTAAGCTAATACG
**hElp6_ F**	AAACCTAATGATGCCTGATGTTTAAATCGACCTACTCCGGAATATTAATAGATC
**hElp6_ R**	AACCCCGATTGAGATATAGATTTATTTAAATGGTGTAGCGTCGTAAGCTAATACG

Abbreviations: F – forward, R – reverse.

Appendix Table S2. Cryo-EM data collection and processing

	**Human Elp456**	**Mouse Elp456**
	EMD-14626	EMD-14627
Magnification	105,000 x	105,000 x
Voltage (kV)	300	300
Electron exposure (e-/Å^2)	41	41
Defocus range (µm)	1-2.5	1-2.5
Pixel size (Å)	0.86	0.86
Symmetry imposed	C2	C2
Initial particle images (no.)	11472	4121
Initial particles (no.)	209492	336406
Map resolution (Å, FSC 0.143)	4.32	4.03
Map resolution range (Å)	3.7 -11	3.5 -10
Map sharpening *B* factor (Å^2)	204.3	174.4
