# Supplementary material for: Functional divergence of the two Elongator subcomplexes during neurodevelopment
Source: EMBO Mol Med. 2022 Jun 13;14(7):e15608. doi: 10.15252/emmm.202115608 (PMC9260213; doi:10.15252/emmm.202115608)
Supplement: Supplementary file 2 — Expanded View Figures PDF [file EMMM-14-e15608-s003.pdf]

Expanded View Figures

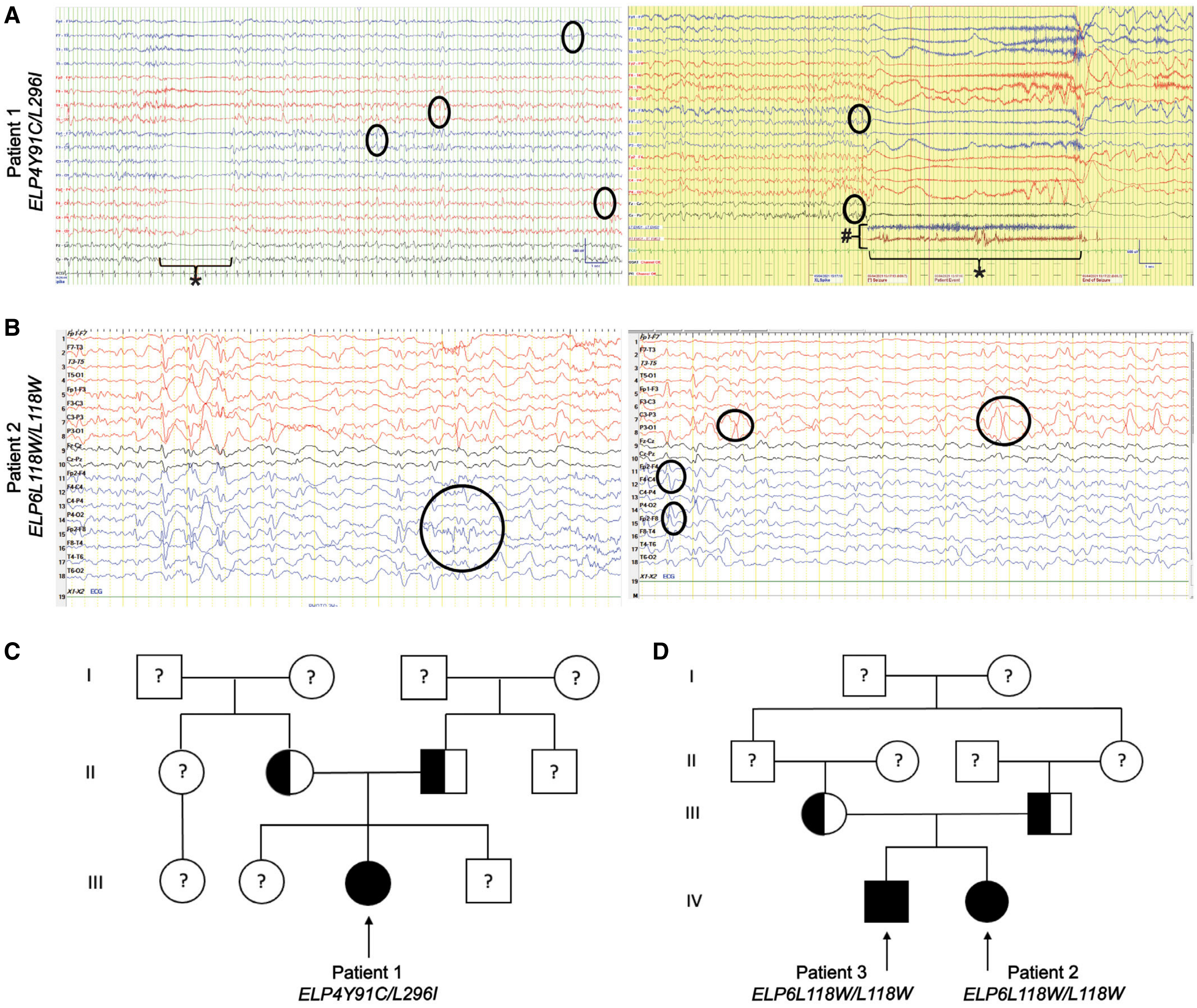

Figure EV1. Representative EEG recordings and pedigree trees of the patients with identified *ELP4* and *ELP6* variants.

A Bipolar EEG montage for Patient 1. Left panel: Frequent interictal epileptiform discharges (few examples circled) are seen in the bilateral hemispheres. These occur in non-evolving runs lasting up to bilateral posterior head regions for seconds. Occasional to intermittent, low-amplitude, attenuated cerebral activity of electrodecremental episodes (*) without clinical signs are captured. Right panel: A typical electroclinical seizure (*) consisting of arm and leg extension, is captured during the recording. One of the legs may be shaking, possibly due to vibration tonic posturing. Low to medium-amplitude polyspikes (examples circled) over the bilateral frontal central region followed by high-amplitude slow waves and low-amplitude fast waves with muscle artifact (#). The initial polyspikes varied in length and locations. The seizures vary from 6 to 47 s.

B EEG of Patient 2. Left panel: Bilateral centroparietal-parietooccipital multispike-wave activities and bilateral frontocentral-frontotemporal sharp-slow wave discharges occur asynchronously. Right panel: Centroparietal-parietooccipital (indicated on left) and frontocentral-frontotemporal (indicated on right) sharp and slow multifocal wave activities occur by forming phase encounters. Described changes are marked by circles.

C Pedigree map of the patient with *ELP4Y91C/L296I* variants. Squares indicate male and circles female family members. Solid symbols mark the affected patient (arrowhead; Patient 1), half-filled symbols the variant carriers and question marks unknown genotypes.

D Pedigree map of the patients with *Elp6L118W* variants. Squares indicate male and circles female family members. Solid symbols mark the affected patients (arrowheads; Patients 2 and 3), half-filled symbols the variant carriers and question marks unknown genotypes.

 © 2022 The Authors

Figure EV2. The conservation of human, murine, and yeast Elp456 complexes.

A Workflow scheme for Elp456 protein production from insect cell expression system.

B SDS–PAGE gel showing the purified human, murine and yeast Elp456 complexes with corresponding gel filtration profiles indicating the estimated elution volume of approximately 200 kDa on the Superose 6 Increase 10/300 GL column (left panel). The schematic overview of Elp4, Elp5, and Elp6 proteins from human, mouse, and yeast with the amino acid length indicated (right panel).

C The multisequence alignments of Elp4 (green), Elp5 (blue), and Elp6 (brown) proteins show the evolutionary conservation of amino acid sequences.

D Averaged melting curves from thermal shift assay for mElp456 variants with calculated melting temperatures (Tm) (mean ± SD) on the left and their respective first derivative (right panel), $n = 3$ independent measurements.

Source data are available online for this figure.

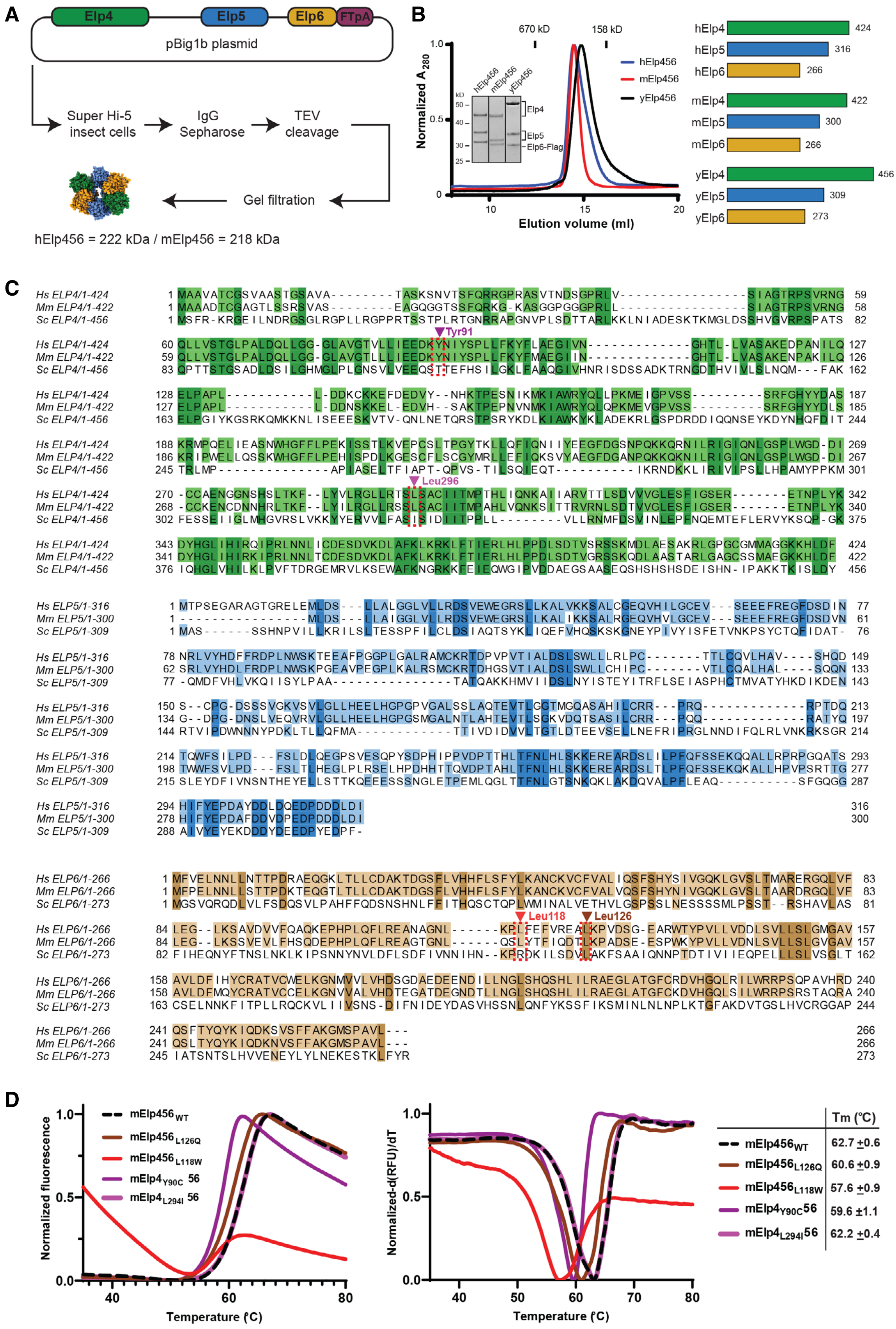

Figure EV2.

 © 2022 The Authors

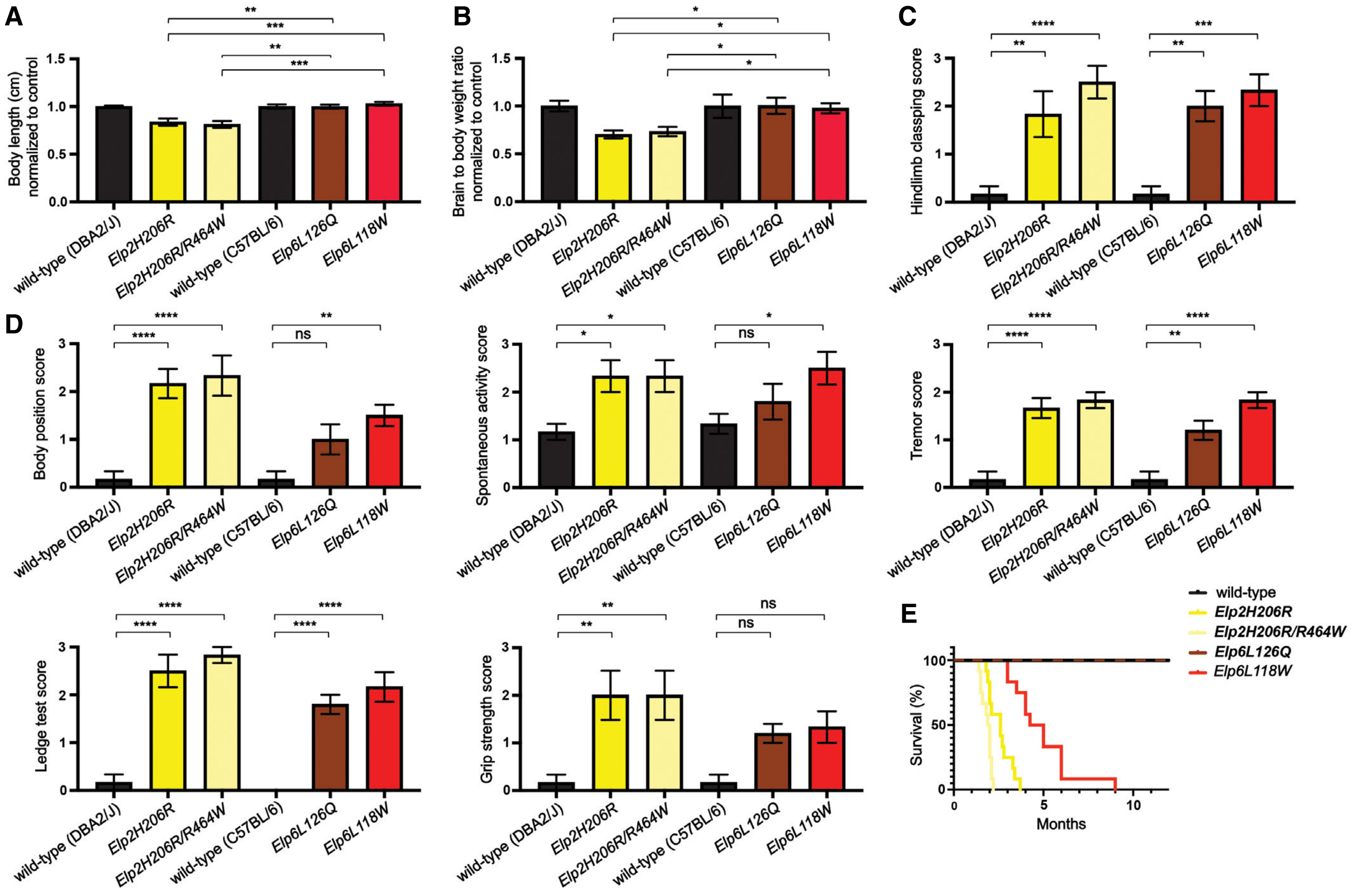

Figure EV3. Phenotypic features of *Elp2* and *Elp6* mutant mice.

A Body size of adult (2-months-old) *Elp2* and *Elp6* mutant mice and their littermate controls (n = 6 for wild-type (DBA2/J and C57BL/6), *Elp2H206R*, *Elp2H206R/R464W*, and *Elp6L118W* animals; n = 5 for *Elp6L126Q* animals).

B Brain weight relative to body weight of adult *Elp2* and *Elp6* mutant mice and their littermate controls (n = 6 for wild-type (DBA2/J and C57BL/6), *Elp2H206R*, *Elp2H206R/R464W*, and *Elp6L118W* animals; n = 5 for *Elp6L126Q* animals).

C Abnormal hindlimb clasping of *Elp2* and *Elp6* mutant animals (n = 6 for wild-type (DBA2/J and C57BL/6), *Elp2H206R*, *Elp2H206R/R464W*, and *Elp6L118W* animals; n = 5 for *Elp6L126Q* animals).

D Significant effects of *Elp2* and *Elp6* mutations on scores for behavioral tests, including body position, spontaneous activity, tremor, ledge test, and grip strength tests animals (n = 6 for wild-type (DBA2/J and C57BL/6), *Elp2H206R*, *Elp2H206R/R464W*, and *Elp6L118W* animals; n = 5 for *Elp6L126Q* animals).

E Kaplan–Meier curve of mouse survival (n = 12 per genotype).

Data information: Statistical analysis: one-way ANOVA (α = 0.05) with a Dunnett's *post hoc* test. Statistically significant differences are indicated (*$P \le 0.05$; **$P \le 0.01$; ***$P \le 0.001$; ****$P \le 0.0001$; ns—not significant). Data represent mean ± SEM.

© 2022 The Authors

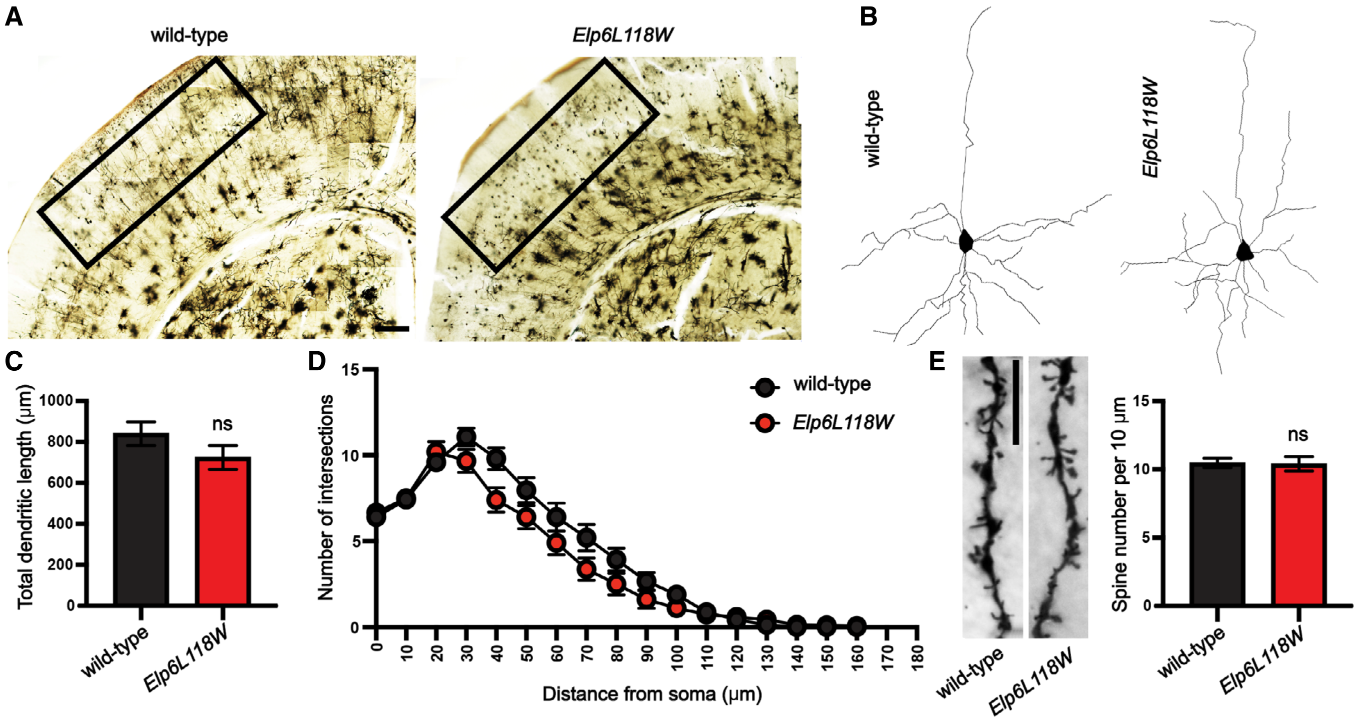

Figure EV4. Normal morphology of cortical pyramidal neurons in *Elp6L118W* mice.

A Representative images of Golgi-Cox-stained coronal brain sections of adult (2-months-old) wild-type and mutant mice. Rectangles represent somatosensory region containing pyramidal neurons selected for neuron reconstruction and subsequent analyses. Scale bar: 100 µm.

B Representative pyramidal neuron basal dendritic tree reconstructions.

C Quantification of total dendritic length from dendritic tree reconstructions shown in (B). $n = 30$; 10 neurons per animal and 3 animals per genotype.

D Sholl analysis of the complexity of basal dendritic arbors. $n = 30$; 10 neurons per animal and 3 animals per genotype.

E Representative images of dendritic spines on basal dendrites of the cortical neurons. Quantification of spine density per 10 µm on secondary dendrites is shown ($n = 15$; 5 neurons per animal and 3 animals per genotype). Scale bar: 10 µm.

Data information: Statistical analysis: unpaired two-tailed *t*-test ($\alpha = 0.05$) with Welch's correction. Holm-Sidak correction was applied to adjust for multiple comparisons (D). ns—not significant. Data represent mean ± SEM (with error bars in D removed when smaller than the corresponding data point).

 © 2022 The Authors

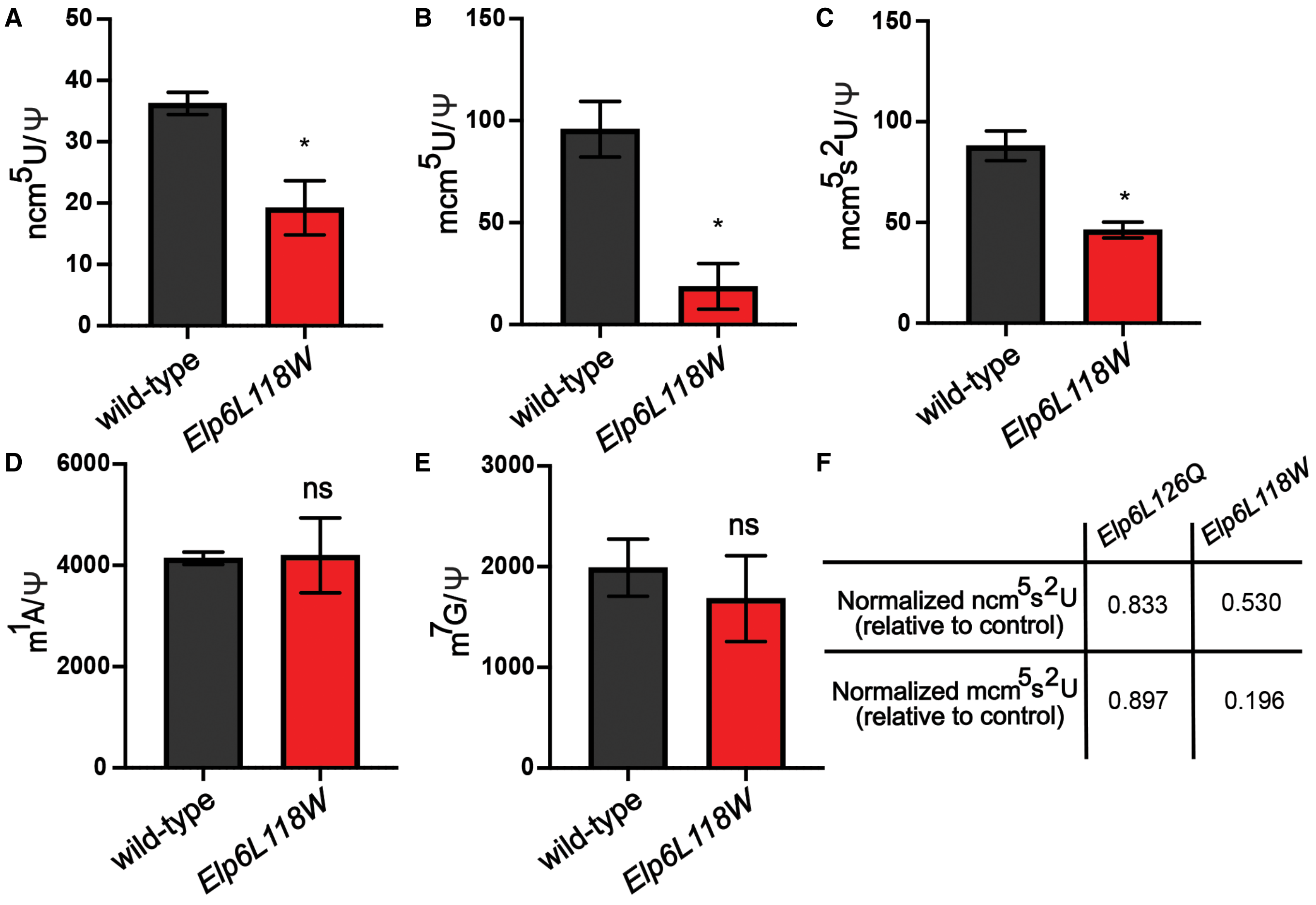

	Elp6L126Q	Elp6L118W
Normalized ncm^5s^2U (relative to control)	0.833	0.530
Normalized mcm^5s^2U (relative to control)	0.897	0.196

Figure EV5. tRNA modification deficiency in *Elp6L118W* murine brain.

A High-performance liquid chromatography (HPLC) coupled to mass spectrometry (MS) used to quantify the Elongator-dependent tRNA modification 5-carbamoylmethyluridine (ncm^5U) in the brain tissue of adult (2-months-old) *Elp6L118W* relative to wild-type animals. Pseudouridine (Ψ) was used as an internal normalization standard. $n = 3$ animals per genotype.

B HPLC-MS quantification of Elongator-dependent tRNA modification 5-methoxy-carbonylmethyluridine (mcm^5U). Pseudouridine (Ψ) was used as an internal normalization standard. $n = 3$ animals per genotype.

C HPLC-MS quantification of Elongator-dependent tRNA modification 5-methoxycarbonylmethyl-2-thiouridine (mcm^5s^2U). Pseudouridine (Ψ) was used as an internal normalization standard. $n = 3$ animals per genotype.

D HPLC-MS quantification of Elongator-independent tRNA modification 1-methyladenosine (m^1A). Pseudouridine (Ψ) was used as an internal normalization standard. $n = 3$ animals per genotype.

E HPLC-MS quantification of Elongator-independent tRNA modification 7-methylguanosine (m^7G). Pseudouridine (Ψ) was used as an internal normalization standard. $n = 3$ animals per genotype.

F Table of comparison of ncm^5U and mcm^5U levels in the brain tissue of *Elp6L126Q* and *Elp6L118W* mice. $n = 3$ animals per genotype.

Data information: Statistical analysis: unpaired two-tailed *t*-test ($\alpha = 0.05$) with Welch's correction. Statistically significant differences are indicated ($^*P \leq 0.05$; ns—not significant). Data represent mean ± SEM.
