# Supplementary figures and images for: Functional divergence of the two Elongator subcomplexes during neurodevelopment
Source: EMBO Mol Med. 2022 Jun 13;14(7):e15608. doi: 10.15252/emmm.202115608 (PMC9260213; doi:10.15252/emmm.202115608)

Source data for Expanded Figure EV2B

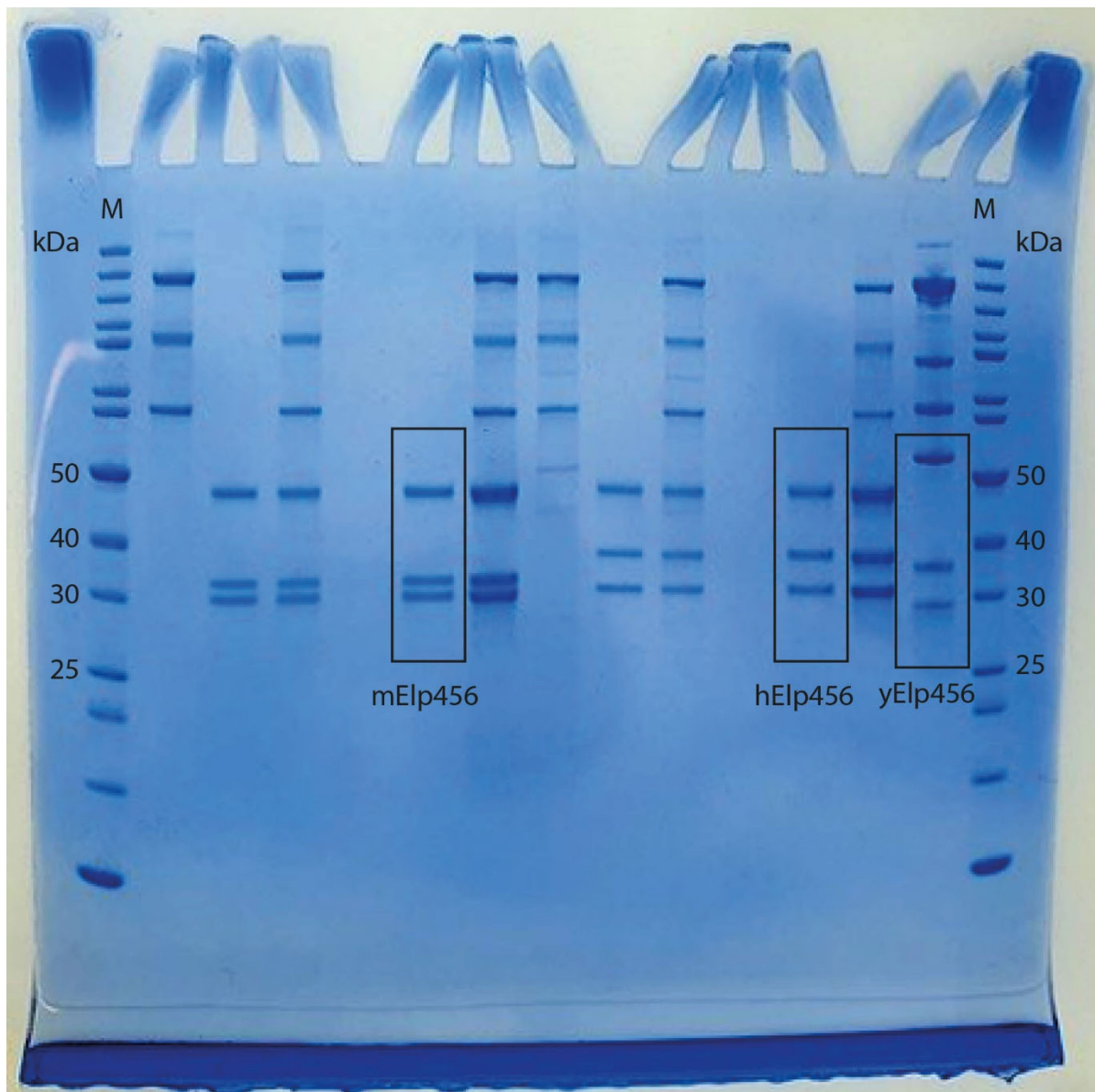

Coomassie stain

Supplement: Supplementary file 3 — Source Data for Expanded View [file EMMM-14-e15608-s001.zip › EMM-2021-15608-V3-Figure_EV2_Source_data-sd.pdf]
