# Supplementary material for: Functional divergence of the two Elongator subcomplexes during neurodevelopment
Source: EMBO Mol Med. 2022 Jun 13;14(7):e15608. doi: 10.15252/emmm.202115608 (PMC9260213; doi:10.15252/emmm.202115608)
Supplement: Supplementary file 4 [file EMMM-14-e15608-s004.pdf]

EMBO Molecular Medicine

Functional divergence of the two Elongator subcomplexes during neurodevelopment

Monika Gaik, Marija Kojic, Megan Stegeman, Tülay Öner, Anna Kościelniak, Alun Jones, Ahmed Mohamed, Pak Yan Chau, Sazia Sharmin, Andrzej Chramiec-Głąbik, Paulina Indyka, Michal Rawski, Anna Biela, Dominika Dobosz, Amanda Millar, Vann Chau, Aycan Ünalp, Michael Piper, Mark Bellingham, Evan Eichler, Deborah Nickerson, Handan Güleryüz, Nour Abbassi, Konrad Jazgar, Melissa Davis, Saadet Mercimek-Andrews, Sultan Cingöz, Brandon Wainwright, and Sebastian Glatt
DOI: 10.15252/emmm.202115608

Corresponding author(s): Sebastian Glatt (sebastian.glatt@uj.edu.pl) , Brandon Wainwright (b.wainwright@imb.uq.edu.au), Sultan Cingöz (sultan.cingoz@deu.edu.tr)

Review Timeline:

Submission Date:	17th Dec 21
Editorial Decision:	20th Jan 22
Revision Received:	29th Apr 22
Editorial Decision:	11th May 22
Revision Received:	20th May 22
Accepted:	23rd May 22

Editor: Jingyi Hou

Transaction Report:

(Note: With the exception of the correction of typographical or spelling errors that could be a source of ambiguity, letters and reports are not edited. Depending on transfer agreements, referee reports obtained elsewhere may or may not be included in this compilation. Referee reports are anonymous unless the Referee chooses to sign their reports.)

1st Editorial Decision 20th Jan 2022

Thank you for submitting your work to EMBO Molecular Medicine. We have now heard back from the three referees who evaluated your manuscript. As you will see from the reports below, the referees acknowledge the potential interest and relevance of the study. However, they also raise a series of concerns about your work, which should be convincingly addressed in a major revision of the present manuscript.

Without reiterating all the points raised in the reviews below, some of the more substantial issues are the following:

- Most of their concerns refer to the need to perform additional experiments and analyses to further improve the molecular mechanisms, which need to be carefully addressed.
- In light of the comments of Referees #2 and #3, please revise the conclusions and discussion to better reflect the actual findings of the study, and overstatements should be avoided.
- Referee #3's concern regarding the electrophysiological study needs to be satisfactorily addressed.

We would welcome the submission of a revised version within three months for further consideration. Please note that EMBO Molecular Medicine strongly supports a single round of revision. As acceptance or rejection of the manuscript will depend on another round of review, your responses should be as complete as possible.

***** Reviewer's comments *****

Referee #1 (Comments on Novelty/Model System for Author):

This is a very novel study but more details on molecular mechanisms would be an added value.

Referee #1 (Remarks for Author):

In the paper entitled "Distinct roles of the two Elongator subcomplexes during neurodevelopment », the authors describe the identification of new variants of both ELP4 and ELP6, two subunits of the Elongator complex involved in tRNA modifications. The patient-derived mutations affect the enzymatic activity of Elongator. Some neuropathology distinct from the ones reported with Elp1-2-3 mutations are described, which opens the possibility that the Elp123 and Elp456 subcomplexes show specific roles on tRNA species and distinct roles in different cell types. As a result, the authors suggest unique roles for each subcomplexes in neurodevelopment. This interesting study provides new insights into the role of Elongator in neurodevelopment and suggests for the first time some specific functions for Elongator subcomplexes in neurodevelopment. A variety of experimental approaches were conducted, which makes this study novel. Some issues can nevertheless be raised and should be experimentally addressed to further improve this study.

1. The authors describe some pull-down experiments of the assembled mElp123456 complexes harboring the reported mutations but these experiments should be complemented with co-immunoprecipitations carried out in mammalian cells to further address whether or not these mutations have any consequence on the assembly of Elongator subunits. More specifically, Elp5 appears to connect Elp3 to Elp4 (Close et al., The Journal of Biological Chemistry, 2012). Does mutated Elp4 still properly bind Elp3 and Elp5? Additional co-IPs would be an added value.
2. The authors mention that the patient-derived Elp4 and Elp6 variants lead to decreased stability of the isolated Elp456 subcomplex. How can we integrate this finding with the fact that the assembly of Elongator does not appear to be modified? This is unclear to me. Would it mean that some variants show some intrinsic instability? The authors should express these mutants in mammalian cells and address the stability/half-life of the resulting proteins in parallel to co-immunoprecipitations described here before. Alternatively, they could look for Elp4 and Elp6 protein levels in mice harboring the corresponding mutations induced by CRISPR-Cas9 and compare these protein levels with wild type proteins. Figure S5 is supposed to describe these western blots but this information is actually lacking in the manuscript. Doing endogenous co-immunoprecipitations of Elongator subunits using protein extracts from this mutated versus wild type mouse model would be very insightful.
3. Cell migration is critically regulated by Elongator. As the authors suggest that Elongator subcomplexes have specific functions in neurodevelopment and because Elp3 promotes cell migration of projection neurons (Creppe et al. Cell, 2009), it would be interesting to see whether fibroblasts from patients harboring Elp4 or Elp6 mutations show some cell migration defects or not.
4. Still with the idea that Elongator subcomplexes have specific functions in neurodevelopment, we would like to learn more on

molecular mechanisms. More specifically, E14.5 forebrains of Elp3 KO mice show signs of unfolded protein response (UPR) (Laguesse et al., Developmental Cell, 2015). Consistently, cortical extracts from E14.5 KO mice show elevated levels of Atf4 (Laguesse et al., Developmental Cell). This phenomenon contributes to microcephaly observed in mice lacking Elp3 in cortical neurons. As the mouse harboring the Elp6L126Q or Elp6L118W mutation does not show microcephaly, one may expect not to see any Aft4 stabilization in cortical extracts from this experimental model. This issue should be experimentally addressed to learn more on molecular mechanisms. This issue appears critical to me as Atf4 stabilization is a common feature shared by multiple experimental models lacking Elp3. In this context, the authors should address whether Atf4 is stabilized in pyramidal and Purkinje neurons from the Elp6L126Q or the Elp6L118W mouse, where some defects are reported.
5. If Atf4 stabilization is not observed in cortical or purkinje neurons from mice harboring the Elp6L126Q or the Elp6L118W mutation (despite the reported UPR signature...), transcriptional analyses combined with GSEA should be carried out to reveal other molecular defects, including cell death.
6. There is some confusion in Figure S5 as some western blots are described in the legend but are not illustrated in the corresponding figure. Figure S5 only shows level of several tRNA modifications in WT versus mutated mice.
7. Some interesting speculation is made in the discussion. Indeed, the authors suggest that some specific tRNAs targeted by the Elp456 subcomplex may not be produced in all cell types. Some attempts should be described to assess this issue as it may indeed explain the specific defects seen in mice harboring the Elp6L126Q or the Elp6L118W mutation.

Referee #2 (Remarks for Author):

The paper by Gaik and colleagues describes the identification of variant mutations in the ELP4 and ELP6 genes in patients with developmental delay, ID and motor dysfunction. Because ELP4 and ELP6 are part of the same ELP subcomplex, they set out to determine the structure of the human and murine ELP456 subcomplexes. This was further used to study the impact of the identified mutations on both the stability of the subcomplex and the activity of the whole ELP complex. Next, the authors generated a mouse model that carries the ELP6L118W mutation found in patients, which recapitulates the clinical phenotype and highlights neuronal defects in specific areas of the mouse brain. These neurological defects were distinct from those observed in animals carrying mutations in the ELP123 subcomplex. The authors conclude that mutations in ELP4 and ELP6 lead to decreased stability of the ELP456 subcomplex, to reduction of the whole ELP activity and to specific neurological defects that are distinct from those observed upon LoF of the ELP123 subcomplex. This led them conclude that the two ELP subcomplexes (123 and 456) have distinct roles in different cell types and in different steps of neurodevelopment.
The paper is overall very interesting and well presented. The identification of novel variants in ELP proteins responsible for neurodevelopmental defects is important and have high clinical implications. This is nicely combined with an in depth /comprehensive structural and functional analysis the ELP456 subcomplex (WT and mutants). The clinical relevance of the ELP6L118W mutation is further highlighted by the generation of the unique corresponding mouse model, which show specific and relevant phenotypes.
Upon addressing the major concerns listed below, the reviewer finds this paper original, relevant and solid; therefore suitable for publication.

Major concerns:
- One major statement of the paper is that the two ELP subcomplexes have distinct roles in different cell types and during neurodevelopment. The authors attribute this to differences in specific binding affinity to different tRNA species. At this stage of the investigation, the authors do not have data supporting this statement. They found that the identified mutations in ELP4 and ELP6 globally affect ELP-dependent tRNA modification, but have different impact on the binding affinity to specific tRNA species.
1/ One should not generally conclude on distinct roles of the ELP subcomplexes based on single mutations in ELP456, which could specifically affect the binding affinity of some but not all tRNA species. Using a complete LoF model of one of the ELP456 subunit (i.e. affecting the ATPase activity) may highlight a more profound, less specific phenotype, which may better resemble the one observed upon LoF of the ELP123 subcomplex. One cannot exclude at this point that the specificity of the observed phenotype is due to the partial (or specific) LoF imposed by the specific mutations (rather than a complete LoF of the proteins). The conclusions and the discussion should be adapted and more balanced on this point. The title should be amended.
2/ It is not clear whether the identified mutations affect the ATPase activity of the ELP456 subcomplex. If not, this may explain the specificity in the phenotype observed in mutant mice (as compared to other ELP LoF models) because it's likely caused by a differential binding affinity to specific tRNAs. In this case, claiming that the two subcomplexes have distinct roles is an overstatement (cfr 1/).
3/ If the two subcomplexes are required for the ELP-dependent tRNA modifications, how do the authors explain the specific requirement of one or the other subcomplex in specific neuronal subtypes? Would the specificity in the role of the ELP456 subcomplex be correlated with differential expression profile of the corresponding subunits in the affected areas? If antibodies are available, the authors should assess the expression of the subunits.

- The authors performed quantitative proteomics using ELP4* and ELP6* patient-derived fibroblasts. From the differential analysis (some changes are observed between control and mutants), they claimed that "imbalanced protein homeostasis" was revealed, which reflect change in "the translational programs of cells". Performing a differential proteomics analysis (alone) is poorly indicative of the role of a protein in translation control (or protein homeostasis). The data (as they are) do not provide

indications that translational programs are changed. Therefore, this statement should be balanced or the authors should further characterize the changes in translational programs (for ex: comparing changes in RNAseq vs proteomics; or -better- performing experiments addressing translation directly).
In the proteomics analysis, do the authors detect any sign of stress response (i.e. ISR, UPR or so; as seen in other models or in appendix figure S3)? or any preferential codon content in mRNAs encoding proteins that are UP or DOWN regulated? Investigating these two options would strengthen the idea that translation programs would be different in WT or mutant lines. The proteomics data certainly deserve a more detailed description and/or a couple of validations.

- Is the different binding affinity of the mutants towards specific tRNA species correlated with different consequences on the U34 modification at these specific tRNAs (for ex: in the dedicated patient-derived fibroblasts)?

- The expression of all the ELP subunits in the patient-derived fibroblasts should be provided.

Minor concern: there is a mistake with Appendix figure S5 (i.e. is similar to EV5)

Referee #3 (Remarks for Author):

In the manuscript by Gaik et al., the authors have 1) characterized the structure and biochemical properties of mammalian ELP123456 complex, 2) demonstrated the physiological importance of ELP4 and ELP6 by identifying pathological mutations in these genes and 3) analyzed neurodevelopmental disorders caused by the equivalent mutations in mice. In addition, to the best of my knowledge, This manuscript shows that mammalian ELP4 is required for ncm5U and mcm5s2U modifications in tRNA and makes important contributions to understand mammalian tRNA modification enzyme and pathophysiology. However, considering the various findings on yeast Elps complex, the present paper does not show conceptually novel molecular mechanisms. In addition, the electrophysiological study is poor designed and has serious problem. The specific comments are as follows;

Major points
Fig. 6G
1. Authors recorded fEPSP for only 30 min after tetanus stimulation. Authors should recorded fEPSP for more than1h to demonstrate LTP.
2. In wild-type mice, fEPSP at 10 min after tetanus stimulation return to baseline. The Reviewer cannot understand the phenomena.

Fig. 6F
1. The amplitude of fEPSP on CA1 evoked by Schaffer collateral stimulation is about several mV. In this manuscript, however, the amplitude is about 20 mV. Why?
2. The time course of an fEPSP in the mutant mice is very slow. The authors should explain the phenomena.

Minor points:
1. In the Abstract, the authors wrote 'Clinically relevant variants have been reported in the catalytic Elp123 subcomplex, while no mutations in the accessory subcomplex Elp456 have been described'. However, there is a previous study that showed that human ELP4 gene deletions were associated with intellectual disability and/or autism spectrum disorder (Addis et al., Human Mutations. 2015. PubMed ID: 26010655), as the authors had cited this paper (Reference 19). The description in the Abstract is misleading, and the authors should change the wording.

2. In the Discussion section, the authors wrote about 'distinct' roles of the Elp123 complex and Elp456 complex, by comparing the brain phenotypes of pathogenic mutant mice; They proposed that the Elp123 complex affects the whole brain and the Elp456 complex affects specific neurons. However, as the authors are comparing pathogenic mutant mice and not knockout mice, I think that the authors are becoming too speculative. For example, different Elp456 mutations might be just as detrimental as Elp123 mutations. In the Discussion section, the authors used words such as 'seem to' or 'appear to', in order not to exclude such possibilities. However, the manuscript title 'Distinct roles of the two Elongator subcomplexes during neurodevelopment' may be too strong. Of course, my understandings may not be deep enough; if so, please argue back.

Point-by-point response

Manuscript number: EMM-2021-15608 Decision Letter

Corresponding author(s): Sultan Cingöz, Brandon J. Wainwright, and Sebastian Glatt

Referee #1 (Comments on Novelty/Model System for Author):

This is a very novel study but more details on molecular mechanisms would be an added value.

Referee #1 (Remarks for Author):

In the paper entitled "Distinct roles of the two Elongator subcomplexes during neurodevelopment», the authors describe the identification of new variants of both ELP4 and ELP6, two subunits of the Elongator complex involved in tRNA modifications. The patient-derived mutations affect the enzymatic activity of Elongator. Some neuropathology distinct from the ones reported with Elp1-2-3 mutations are described, which opens the possibility that the Elp123 and Elp456 subcomplexes show specific roles on tRNA species and distinct roles in different cell types. As a result, the authors suggest unique roles for each subcomplexes in neurodevelopment. This interesting study provides new insights into the role of Elongator in neurodevelopment and suggests for the first time some specific functions for Elongator subcomplexes in neurodevelopment. A variety of experimental approaches were conducted, which makes this study novel. Some issues can nevertheless be raised and should be experimentally addressed to further improve this study.

1. The authors describe some pull-down experiments of the assembled mElp123456 complexes harboring the reported mutations but these experiments should be complemented with co-immunoprecipitations carried out in mammalian cells to further address whether or not these mutations have any consequence on the assembly of Elongator subunits. More specifically, Elp5 appears to connect Elp3 to Elp4 (Close et al., The Journal of Biological Chemistry, 2012). Does mutated Elp4 still properly bind Elp3 and Elp5? Additional co-IPs would be an added value.

Response: We based our conclusions from the pull-down experiments on the available structural model of the highly conserved Elongator complex obtained by a combination of X-ray crystallography, electron microscopy and crosslinking mass spectrometry (Glatt et al. NSMB 2012; Glatt et al. NSMB 2016; Dauden et al. EMBO Rep. 2017; Lin et al. Nat Comm 2019; Dauden et al. Science Adv. 2019). In this model, the Elp456 ring contacts the Elp123 subcomplex in two particular regions, namely the C-terminus of Elp1 and Elp3 – both interaction interfaces are created by the two respective Elp4 molecules within the hexameric Elp456 ring. We present the homology-based model of murine Elongator in Figure 3A and now highlight the two contact points between Elp123 and Elp456 to improve clarity.

Of note, the results of the excellent study by Close and colleagues using shRNA-based analyses are consistent with our structural model of Elongator – even if the model precludes the existence of a direct protein-protein interface between Elp3 and Elp5. The observed interaction between Elp3 and Elp5 is mediated by the other subunits in the complex, which can be detected by Co-IPs. We would like to highlight that our current model of the complex and its specific separation into two subcomplexes, namely Elp123 and Elp456, is in agreement with all existing literature on the assembly and function of the Elongator complex. As the complex works as one single entity and shows complicated interaction networks between individual subunits, one would need to co-overexpress all six subunits in parallel in human cells to test the effects of the respective mutations. At the current stage we are able to co-express all six subunits using the BigBac Baculovirus expression systems in

insect cells, which is the basis of our presented work. Until a suitable co-expression system is available for human cells, our work relies on the mouse and human Elongator complex produced in insect cells.

Nonetheless, we fully agree that the question and suggested experiments are interesting and complementary. Hence, we performed Co-IP analyses in control fibroblasts and patient-derived cell lines, to check whether the assembly of the human Elongator is affected by the mutations in *ELP4* and *ELP6*. In detail, we have used antibodies against ELP1 for immobilization and used antibodies against ELP3, ELP4 and ELP5 to detect the respective interactions by co-precipitation. In our hands, the anti-ELP6 antibody that worked for mouse Elp6, did not work for detecting human ELP6 in lysates or after Co-IP. Our results show that indeed the complex is able to form in the different patient cells that carry the respective mutations. Hence, the mutations do not influence the integrity of the complex in cells, confirming the results from our *in vitro* reconstitution analysis using purified subcomplexes.

We have included the results of the analyses in Figure 3D of the revised manuscript. In addition, we now mention the obtained results on page 8 of the manuscript, which reads as follows – *"Furthermore, we used co-immuno-precipitation analyses (Co-IP) to confirm that the two subcomplexes still assemble in fibroblasts derived directly from the patients (Fig 3D)."* We have also amended the materials and methods section and added the respective descriptions of the experiments.

2. The authors mention that the patient-derived Elp4 and Elp6 variants lead to decreased stability of the isolated Elp456 subcomplex. How can we integrate this finding with the fact that the assembly of Elongator does not appear to be modified? This is unclear to me. Would it mean that some variants show some intrinsic instability? The authors should express these mutants in mammalian cells and address the stability/half-life of the resulting proteins in parallel to co-immunoprecipitations described here before. Alternatively, they could look for Elp4 and Elp6 protein levels in mice harboring the corresponding mutations induced by CRISPR-Cas9 and compare these protein levels with wild type proteins. Figure S5 is supposed to describe these western blots but this information is actually lacking in the manuscript. Doing endogenous co-immunoprecipitations of Elongator subunits using protein extracts from this mutated versus wild type mouse model would be very insightful.

Response: The thermostability analyses shows that mutated mElp456s unfold at a lower temperature than wildtype mElp456 (Figure EV2D). Though, the hexameric ring carrying the strongest affected variant (Elp6 L118W) still remains intact up to ~50 °C. Hence, the finding that the assembly of the full complex still forms at lower temperatures (e.g. body temperature) is fully consistent with our findings. Nonetheless, the measured effect shows that the mutation does influence the stability of the assembly, most likely reducing rigidity and inducing additional flexibility and dynamics. The additional flexibility will lead to weaker (but not diminished) inter-subunit interactions at lower temperatures and facilitate the disassembly and unfolding of the hexameric ring at higher temperatures. We have now added an additional explanation to the discussion section of the revised manuscript, which reads as follows – *"Disease-associated mutations in Elp456 may still support the assembly of the complex at physiologically relevant temperatures, but lead to a greater reduction of activity, because its action is required only for a subfraction of neuronal cell types during development."*

We do agree with the reviewer that it is important to check the influence of the respective mutation on the expression/stability of the other complex components. In this respect, we would like to apologize to the reviewers and the editor – we had to realize that during the final preparation steps of the manuscript a second copy of Figure EV5 was mistakenly incorporated in the position of Appendix Figure S5. The original figure shows that in the brain tissue of the *Elp6L118W* mice, the level of Elp6 is significantly reduced. In contrast, the levels of Elp1, Elp2 and Elp4 remain unchanged. Even

if the levels of Elp4 are unchanged, the reduced levels of Elp6 will indeed lead to reduced levels of functional Elp456. These data are now included in Appendix Fig S6C,D (which had to be renumbered after the introduction of the newly incorporated Appendix Figure S2).

We believe the overexpression of individual subunits of a large macromolecular complex in mammalian cells is very likely leading to misinterpretations (see previous comment). In short, the overexpression could lead to effects that are related to the production of mutated proteins that cannot be assembled into their native complex, as the other subunits are not overexpressed at the same time. Hence, we have determined the expression levels of Elongator subunits in the patient-derived fibroblast cell lines (Appendix Fig S2A; text related to the figure on page 8 in the manuscript) and performed Co-IP experiments in these cell lines (see above). Please note, that the anti-Elp6 antibody worked only for the detection of Elp6 from mouse and we were not able to detect a reliable signal for human Elp6. We do believe that our incorporated data addresses the raised issue directly.

3. Cell migration is critically regulated by Elongator. As the authors suggest that Elongator subcomplexes have specific functions in neurodevelopment and because Elp3 promotes cell migration of projection neurons (Creppe et al. Cell, 2009), it would be interesting to see whether fibroblasts from patients harboring Elp4 or Elp6 mutations show some cell migration defects or not.

Response: The suggested experiments are indeed a perfect complementation to our study, and we have performed wound healing assays using control fibroblasts and patient-derived cell lines carrying *ELP4* and *ELP6* mutations. We have used mitomycin C as a proliferation inhibitor to eliminate the contribution of cell division to wound closure. Our results show that over a period of 24 hours, we were not able to detect any differences in cell migration between control and patient fibroblasts. Though, we would like to highlight that there might be a fundamental difference in analyzing the missense mutations identified in the patients and a complete LOF of Elongator subunits that was done in the previous studies (Creppe et al. Cell, 2009; Close et al. J Biol Chem, 2012). In addition, fibroblasts might not display the same defects that are more pronounced to neuronal cells.

We have now included a new Appendix Figure S2, which describes the results from these assays (Appendix Figure S2C) and combines them with additional analyses of the same cell lines (e.g. see answer to comment 4 of reviewer 2). We have also amended the materials and methods section and added the respective description of the experiments. The newly incorporated study is mentioned on pages 9/10 of the revised manuscript and reads as follows – "*Given that cytoskeleton is being affected by the variants and the previous studies showing that LOF of Elongator subunits can lead to defects in cell migration[36,37], we have tested the patient-derived fibroblasts in a wound healing assay. After inhibition of proliferation by mitomycin C, we were not able to detect any difference between control fibroblasts and the cells carrying the mutations in ELP4 or ELP6 (Appendix Fig S2C)*."

4. Still with the idea that Elongator subcomplexes have specific functions in neurodevelopment, we would like to learn more on molecular mechanisms. More specifically, E14.5 forebrains of Elp3 KO mice show signs of unfolded protein response (UPR) (Laguesse et al., Developmental Cell, 2015). Consistently, cortical extracts from E14.5 KO mice show elevated levels of Atf4 (Laguesse et al., Developmental Cell). This phenomenon contributes to microcephaly observed in mice lacking Elp3 in cortical neurons. As the mouse harboring the Elp6L126Q or Elp6L118W mutation does not show microcephaly, one may expect not to see any Aft4 stabilization in cortical extracts from this experimental model. This issue should be experimentally addressed to learn more on molecular mechanisms. This issue appears critical to me as Atf4 stabilization is a common feature shared by multiple experimental models

lacking Elp3. In this context, the authors should address whether Atf4 is stabilized in pyramidal and Purkinje neurons from the Elp6L126Q or the Elp6L118W mouse, where some defects are reported.

Response: We would like to highlight that our mouse models differ from conditional knock-out studies and our previous analyses of *Elp2* mutations already revealed mechanistic differences (Kojic et al. Nature Communications, 2021). In detail, we did not find upregulation of any of the UPR markers in the E14.5 forebrains of the *Elp2* mutant mice. Given that we did not identify any cortical defect (including microcephaly) in the *Elp6L118W* mice, we would not expect to see evidence of UPR, such as Atf-4 upregulation/stabilization.

To address the reviewer's comment, we used an anti-Atf-4 antibody to quantify protein level of Atf-4 in the murine brain cortex and patient-derived fibroblasts. We did not find any difference in Atf-4 expression, which we now include in the Appendix Figure S6A,B. We would like to highlight that the protein levels of ATF-4 in human fibroblasts is rather low in comparison to other tested cell lines (e.g. HEK293). Although, the signal is rather weak, we have not seen any signs of significant stabilization. We added an additional sentence describing the newly obtained results on page 13 of the manuscript, which reads as follows – "*As we did not find evident microcephaly and cortical defects in the Elp6 mutants, we hypothesized that UPR was limited to the PNs. To test this, we analyzed the expression of one of the main transcriptional effectors of UPR, activating transcription factor 4 (Atf-4), previously linked to neurogenesis defects in the murine Elp3-depleted cortical neurons[31]. Indeed, UPR was not evident in the cerebral cortex of the Elp6L118W animals (Appendix Fig S6A). Moreover, we found that ATF-4 expression was not affected in the patient fibroblasts as well (Appendix Fig S6B).*"

5. If Atf4 stabilization is not observed in cortical or purkinje neurons from mice harboring the Elp6L126Q or the Elp6L118W mutation (despite the reported UPR signature...), transcriptional analyses combined with GSEA should be carried out to reveal other molecular defects, including cell death.

Response: Purkinje neurons cannot be isolated due to their long dendrites and axons that get easily thorn during isolation process. Hence, we are unfortunately not able to conduct RNAseq analyses of Purkinje neurons. We have attempted to do this in past with GFP-labelled PNs, but the cells were dying quickly during the preparation procedure and the obtained RNA quality was very poor. Hence, the transcriptional signatures of dying neurons would be overshadowed by stress signals and not very informative. As we did not observe a phenotype or UPR in the cortex, we do not see the necessity of performing RNAseq of cortical tissue.

6. There is some confusion in Figure S5 as some western blots are described in the legend but are not illustrated in the corresponding figure. Figure S5 only shows level of several tRNA modifications in WT versus mutated mice.

Response: As mentioned above, we are truly sorry for the confusion. We have now incorporated the correct Figure (now Appendix Figure S6C), which shows the protein expression analyses for the various Elongator subunits in the murine brain.

7. Some interesting speculation is made in the discussion. Indeed, the authors suggest that some specific tRNAs targeted by the Elp456 subcomplex may not be produced in all cell types. Some attempts should be described to assess this issue as it may indeed explain the specific defects seen in mice harboring the Elp6L126Q or the Elp6L118W mutation.

Response: We fully agree with the reviewer that the expression profiling of individual tRNAs in specific neuronal subtypes would be very interesting. Though, the suggested analyses are impeded by several technical difficulties. First, the clean separation of neuronal cell types is technically very challenging and the obtainable quantities to prepare an analyzable tRNA pool are insufficient for quantitative analyses of individual tRNA species. Second, the isolation of individual tRNA species not only requires large amounts of starting material but is also complicated by the presence of a large number of similar iso-decoders in humans. At the current stage, our *in vitro* analyses are indicative, and we will work on establishing methods in animal/human material to answer this question. We are afraid that overcoming the technical limitations goes far beyond the scope of this manuscript and the provided timeframe for the revision.

Referee #2 (Remarks for Author):

The paper by Gaik and colleagues describes the identification of variant mutations in the ELP4 and ELP6 genes in patients with developmental delay, ID and motor dysfunction. Because ELP4 and ELP6 are part of the same ELP subcomplex, they set out to determine the structure of the human and murine ELP456 subcomplexes. This was further used to study the impact of the identified mutations on both the stability of the subcomplex and the activity of the whole ELP complex. Next, the authors generated a mouse model that carries the ELP6L118W mutation found in patients, which recapitulates the clinical phenotype and highlights neuronal defects in specific areas of the mouse brain. These neurological defects were distinct from those observed in animals carrying mutations in the ELP123 subcomplex. The authors conclude that mutations in ELP4 and ELP6 lead to decreased stability of the ELP456 subcomplex, to reduction of the whole ELP activity and to specific neurological defects that are distinct from those observed upon LoF of the ELP123 subcomplex. This led them conclude that the two ELP subcomplexes (123 and 456) have distinct roles in different cell types and in different steps of neurodevelopment.

The paper is overall very interesting and well presented. The identification of novel variants in ELP proteins responsible for neurodevelopmental defects is important and have high clinical implications. This is nicely combined with an in depth /comprehensive structural and functional analysis the ELP456 subcomplex (WT and mutants). The clinical relevance of the ELP6L118W mutation is further highlighted by the generation of the unique corresponding mouse model, which show specific and relevant phenotypes.

Upon addressing the major concerns listed below, the reviewer finds this paper original, relevant and solid; therefore suitable for publication.

Major concerns

One major statement of the paper is that the two ELP subcomplexes have distinct roles in different cell types and during neurodevelopment. The authors attribute this to differences in specific binding affinity to different tRNA species. At this stage of the investigation, the authors do not have data supporting this statement. They found that the identified mutations in ELP4 and ELP6 globally affect ELP-dependent tRNA modification, but have different impact on the binding affinity to specific tRNA species.

1) One should not generally conclude on distinct roles of the ELP subcomplexes based on single mutations in ELP456, which could specifically affect the binding affinity of some but not all

tRNA species. Using a complete LoF model of one of the ELP456 subunit (i.e. affecting the ATPase activity) may highlight a more profound, less specific phenotype, which may better resemble the one observed upon LoF of the ELP123 subcomplex. One cannot exclude at this point that the specificity of the observed phenotype is due to the partial (or specific) LoF imposed by the specific mutations (rather than a complete LoF of the proteins). The conclusions and the discussion should be adapted and more balanced on this point. The title should be amended.

Response: We fully agree with the reviewer that we cannot exclude the specific effect of an individual mutation. We have revised the manuscript in order to make it clear that our findings show the differences between the mutations in the two subcomplexes in respect to their consequences on the neurodevelopment but cannot exclude the possibilities highlighted by the reviewer. We have also revised the title of the manuscript accordingly – we now suggest "*Functional divergence of the two Elongator subcomplexes during neurodevelopment*". Though, we would like to highlight that we see very similar and consistent phenotypic for 2 separate mutations in Elp2 (Kojic et al., 2021) and 2 separate mutations in Elp6 (Kojic et al., 2018 and this study). The differences between the respective Elp2 and Elp6 mutation are highlighted Figure EV3. Single amino acid substitutions very rarely cause a complete LoF, especially when they are not located in central regions of the protein. To our knowledge all attempts to generate complete LoF models for any of the 6 Elongator subunits led to embryonic lethality, which is the reason for numerous conditional knock-out studies. These studies are very important contributions, but do not directly allow to recapitulate the clinical setting and provide direct insights into the molecular reasons for the observed diseases. Therefore, our patient-derived amino acid substitutions offer very valuable *in vitro* and *in vivo* models for studying their effect on tRNA-modifications and epitranscriptomic processes.

2) It is not clear whether the identified mutations affect the ATPase activity of the ELP456 subcomplex. If not, this may explain the specificity in the phenotype observed in mutant mice (as compared to other ELP LoF models) because it's likely caused by a differential binding affinity to specific tRNAs. In this case, claiming that the two subcomplexes have distinct roles is an overstatement (cfr 1/).

Response: We have attempted to measure the ATPase activity of mElp456 and hELP456 in the presence and absence of different tRNA species. In contrast to Elp456 from yeast, we could not detect a basal or tRNA-induced activity. Using a fluorophore-conjugated ATP molecules we have observed signal quenching suggesting ATP binding to the wild-type Elp456, despite no ATP hydrolysis detected. Hence, we were not able to compare the effect of the mutations on the activity. Of note, we were able to reconstitute the fully assembled Elongator complex without the addition of ATP, indicating that the ATPase activity of Elp456 is not required for the interaction between Elp123 and Elp456. We have also created and tested the ATPase-deficient mElp456 mutant for tRNA binding affinity, which was weaker than the wt Elp456, indicating that proper tRNA binding is required for subsequent modification steps (data not shown). We would like to highlight that to our knowledge all ELP LOF models are embryonically lethal, and that conditional knock out model are not able to provide tissue-specific analyses beyond the targeted cell types themselves (see comment above). We would also like to highlight that both, Elp123 and Elp456, can bind tRNA separately. We can assume that mutations in Elp456 do not affect the binding of different tRNAs to Elp123 and we don't see an adequate experimental setup to test this with excluding the possibility of Elp456 contributing to the outcomes.

3) If the two subcomplexes are required for the ELP-dependent tRNA modifications, how do the authors explain the specific requirement of one or the other subcomplex in specific neuronal subtypes? Would the specificity in the role of the ELP456 subcomplex be correlated with

differential expression profile of the corresponding subunits in the affected areas? If antibodies are available, the authors should assess the expression of the subunits.

Response: We have assessed the expression of Elp1, Elp2, Elp4 and Elp6 using western blot, but unfortunately, the antibodies do not work in immunofluorescence. Hence, we are not able to assess the expression of different subunits in different neuronal subtypes. Nevertheless, various brain atlases available with ISH and proteomics analysis of different neurons show that all Elongator subunits are widely expressed in different neurons. We speculate that the mutations in the catalytic subcomplex affect all Elongator-dependent tRNA species and thus, impact various neuronal subtypes, whilst Elp456 mutations affect binding of only a subset of Elongator-dependent tRNAs. Here, our *in vitro* analyses of the the Elp4/6 variants showed that the binding of $tRNA^{Ala}$ is more affected than the $tRNA^{Arg}$. Hence, only a subset of neuronal subtypes would be affected by the mutations in the accessory subcomplex if we assume that not all Elongator-dependent tRNAs are expressed in different neurons. At the current stage, our *in vitro* analyses can indeed only be indicative, but to our knowledge these results also represent the first observation that a tRNA specificity exists for the Elongator subcomplexes.

4) The authors performed quantitative proteomics using ELP4* and ELP6* patient-derived fibroblasts. From the differential analysis (some changes are observed between control and mutants), they claimed that "imbalanced protein homeostasis" was revealed, which reflect change in "the translational programs of cells". Performing a differential proteomics analysis (alone) is poorly indicative of the role of a protein in translation control (or protein homeostasis). The data (as they are) do not provide indications that translational programs are changed. Therefore, this statement should be balanced or the authors should further characterize the changes in translational programs (for ex: comparing changes in RNAseq vs proteomics; or -better- performing experiments addressing translation directly).
   In the proteomics analysis, do the authors detect any sign of stress response (i.e. ISR, UPR or so; as seen in other models or in appendix figure S3)? or any preferential codon content in mRNAs encoding proteins that are UP or DOWN regulated?
   Investigating these two options would strengthen the idea that translation programs would be different in WT or mutant lines. The proteomics data certainly deserve a more detailed description and/or a couple of validations.

Response: We tried our best to revise the statements accordingly and we connected the proteomics analyses with the newly incorporated cell migration and codon bias analyses. We have also revisited our proteomics datasets with a focus on the suggested pathways, but we could not detect up/downregulation of ISR and UPR pathways in ELP4/6 fibroblasts. Moreover, we have performed additional analysis of ATF-4 expression in the patient fibroblasts using western blot and observed no stabilization relative to the control cells (data included in Appendix Fig S6A,B). In addition, we have analyzed the codon content of the mis-regulated proteins to check for a specific codon bias in the identified proteins. We do find a specific enrichment of codons in the mis-regulated fraction of proteins, but these codons include both types of codons – one that are decoded by Elongator modified tRNAs and by tRNAs that are not modified. We have now included these analyses in the newly created Appendix Figure 2 and added an additional paragraph about this data in the results section on page 9 – *"The analyses revealed altered protein expression levels in the patient cell lines (Fig 4F). As the decoding of only a specific subset of codons is affected by the U34 modifications, we have checked for any possible codon bias in the respective mRNA sequences of the mis-regulated proteins. We have found a preference for certain codons in the respective pools of up- and down-regulated proteins*

(Appendix Figure S2B). Though, no direct correlation between the modification-dependent codons and expression levels was observed."

5) Is the different binding affinity of the mutants towards specific tRNA species correlated with different consequences on the U34 modification at these specific tRNAs (for ex: in the dedicated patient-derived fibroblasts)?

Response: Indeed, our study highlights the importance of these types of analyses in the future, which will be necessary to understand the underlying molecular mechanism. We also understand that our presented work directly asks for the suggested experiments, which we have intensively discussed among ourselves. We tried to express this issue in the discussion section of the original version of manuscript – *"Hence, it would also be of high interest to correlate our observations with cell type specific tRNA iso-decoder expression data in vivo once reliable data on neuronal subtypes becomes available in the future."* Though, at the current stage several technical issues need to be overcome to perform such analyses. The main challenge is related to specific isolation of sufficient quantities of individual tRNAs from human cells and the available detection methods for U34 modifications. The recent appearance of sequencing-based methods (e.g. mim-tRNAseq) or Nanopore based techniques will allow us to quantify the cell type specific expression levels of individual tRNA species and to simultaneously determine modification patterns of individual tRNA species. Foremost, these analyses would be ideally performed in different neuronal subtypes to understand the observed phenotypical differences. As described above, the isolation of certain neuronal subtypes (e.g. Purkinje neurons) remains challenging by itself and the obtainable quantities are insufficient to address the issue at this point of time. We fully agree with the reviewer that the issue would be of highest interest and our presented results have ensured us to follow up on these experiments in the future. For now, the suggested analyses are beyond the scope of this revision.

6) The expression of all the ELP subunits in the patient-derived fibroblasts should be provided.

Response: To directly address this comment, we used commercially available antibodies to analyze the expression levels of different Elongator subunits in the patient-derived fibroblasts. The additional analyses have been incorporated in the newly created Appendix Figure 2 (Appendix Fig S2A).

Minor concern

There is a mistake with Appendix figure S5 (i.e. is similar to EV5)

Response: As mentioned above, we are truly sorry for the confusion. We have now incorporated the correct Figure (now Appendix Figure S6C), which shows the protein expression analyses for the various Elongator subunits in the murine brain.

Referee #3 (Remarks for Author):

In the manuscript by Gaik et al., the authors have 1) characterized the structure and biochemical properties of mammalian ELP123456 complex, 2) demonstrated the physiological importance of ELP4 and ELP6 by identifying pathological mutations in these genes and 3) analyzed neurodevelopmental disorders caused by the equivalent mutations in mice. In addition, to the best of my knowledge, This manuscript shows that mammalian ELP4 is required for ncm5U and mcm5s2U modifications in tRNA and makes important contributions to understand mammalian tRNA modification enzyme and pathophysiology. However, considering the various findings on yeast Elps

complex, the present paper does not show conceptually novel molecular mechanisms. In addition, the electrophysiological study is poor designed and has serious problem. The specific comments are as follows;

Major points

Fig. 6G

1. Authors recorded fEPSP for only 30 min after tetanus stimulation. Authors should recorded fEPSP for more than1h to demonstrate LTP.

Response: LTP can undergo many phases in its development, some of which can take longer than 30 minutes post induction to be fully expressed. However, it is widely accepted that significant increases in synaptic transmission that persist > 30 minutes post induction constitute evidence of the early phase of LTP.

2. In wild-type mice, fEPSP at 10 min after tetanus stimulation return to baseline. The Reviewer cannot understand the phenomena.

Response: The continuous potentiated fEPSP with a gradual decrease that is seen in most typical LTP experiments in the current literature is due to blockage of synaptic inhibition during the course of LTP induction in order to obtain a maximum LTP effect (Bortolotto ZA, Anderson WW, Isaac JTR, and Collingridge GL. Synaptic plasticity in the hippocampal slice preparation. In: Current Protocols in Neuroscience, 2001.). However, we did not use any exogenous synaptic inhibitors in our LTP experiments, so LTP is generally smaller and its time course is more variable. The key hallmark of LTP is a significant increase in fEPSP size 30 minutes after induction, which we have demonstrated in the WT mice.

Fig. 6F

1. The amplitude of fEPSP on CA1 evoked by Schaffer collateral stimulation is about several mV. In this manuscript, however, the amplitude is about 20 mV. Why?

Response: We apologize that there is a mistake on the scaling. The amplifier setting used in this experiment is set at 10x, hence the scale bar should be labelled as 1 mV and the amplitude is about 2 mV instead. We have corrected the respective axis and we are very thankful for pointing us towards this mislabeling.

2. The time course of an fEPSP in the mutant mice is very slow. The authors should explain the phenomena.

Response: By the time course of an fEPSP, we assume that the reviewer is referring to the slope of fEPSP. Decreased fEPSP slope in mutant mice indicates a weaker level of synaptic transmission at CA3-CA1 synapses in the hippocampal slice. Stimulation of the Schaffer collaterals will directly excite CA1 pyramidal cells via a glutamatergic input. However, these CA1 pyramidal neurons receive a delayed inhibitory postsynaptic potential (IPSP) as Schaffer collaterals also excite GABAergic interneurons which in turn synapse on the pyramidal cell, so the EPSP is followed by an IPSP with approximately 2 ms delay. This IPSP is normally responsible for keeping the duration of the fEPSP in WT mice short, as shown in Figure 6F. Weaker excitation by Schaffer collaterals will reduce the delayed IPSP in CA1 pyramidal neurons, prolonging the decay of the fEPSP. Also, with the significant reduction in both hippocampal pyramidal neuronal length and branching found in mutant mice, this should result in fewer excitatory synapses than in WT mice, hence, a lesser amount of excitatory synaptic transmission onto CA1 pyramidal neurons and interneurons. Although Elp6 mutant mice did not present any

interneuron and myelination deficits, reduced synaptic inhibition of the CA1 pyramidal cell could have occurred as a result of an overall decreased synaptic excitation by the Schaffer collateral. Consequently, reduction in both excitatory and inhibitory synaptic transmission onto pyramidal cells would account for a weaker synaptic response towards electrical stimulation and slower excitatory neurotransmission.

Minor points

1. In the Abstract, the authors wrote 'Clinically relevant variants have been reported in the catalytic Elp123 subcomplex, while no mutations in the accessory subcomplex Elp456 have been described'. However, there is a previous study that showed that human ELP4 gene deletions were associated with intellectual disability and/or autism spectrum disorder (Addis et al., Human Mutations. 2015. PubMed ID: 26010655), as the authors had cited this paper (Reference 19). The description in the Abstract is misleading, and the authors should change the wording.

Response: We would like to highlight that the cited study describes genomic alterations at Elp4 locus, but the mentioned study does not analyze the Elp4 protein levels or the consequence for the other Elongator subunits. The patient described in our study is heterozygous for 2 single amino acid substitutions, which lead to the production of the mutated variants of the Elp4 protein – see expression analyses in the patient fibroblasts (see Appendix Figure S2A). We have revised the abstract to accentuate this difference. We have added a new paragraph in the introduction on page 4 to describe previous studies on Elp4 in greater detail – *"In addition, the ELP4-PAX6 locus has been linked to Rolandic epilepsy26,27, which was questioned by others21. As the genomic alterations seem to affect the non-coding and intronic regions, the ELP4 protein remains most likely unaffected and the misregulation of PAX6 seems more likely to cause the condition27.*

2. In the Discussion section, the authors wrote about 'distinct' roles of the Elp123 complex and Elp456 complex, by comparing the brain phenotypes of pathogenic mutant mice; They proposed that the Elp123 complex affects the whole brain and the Elp456 complex affects specific neurons. However, as the authors are comparing pathogenic mutant mice and not knockout mice, I think that the authors are becoming too speculative. For example, different Elp456 mutations might be just as detrimental as Elp123 mutations. In the Discussion section, the authors used words such as 'seem to' or 'appear to', in order not to exclude such possibilities. However, the manuscript title 'Distinct roles of the two Elongator subcomplexes during neurodevelopment' may be too strong. Of course, my understandings may not be deep enough; if so, please argue back.

Response: We believe that our work does for the first time suggest different roles for the two Elongator subcomplexes during neurodevelopment. We don't pretend that we have understood the whole molecular mechanisms that underly these different roles. Nonetheless, our work marks a fundamental shift in understanding the complex in the central nervous system of higher eukaryotes. All previously existing models and conclusions were deducted from yeast, where all six subunits are equally important and lead to identical stress phenotypes. Our study clearly shows that in mice, mutations in Elp123 and Elp456 lead to different phenotypes. Hence, we have revised the title of manuscript, accordingly – we now suggest "Functional divergence of the two Elongator subcomplexes during neurodevelopment".

Thank you for the submission of your revised manuscript to EMBO Molecular Medicine. We have now received the enclosed report from the three referees who were asked to re-assess it. As you will see, the referees are now supportive, and I am pleased to inform you that we will be able to accept your manuscript pending the following amendments:

1. Please address the remaining comment from Referee #2.

On a more editorial level:

***** Reviewer's comments *****

Referee #1 (Remarks for Author):

The authors properly addressed all my comments. Therefore, this revised version is suitable for publication.

Referee #2 (Remarks for Author):

I would like to thank the authors for the extensive revision of their paper. I found the paper suitable for publication as it is.

I would like to share one last comment: in Appendix figure S2B, authors analyzed codon bias (RSCU) in mRNA encoding proteins UP or down regulated in the mutants. I personally find that rather than the bias, the actual frequency of individual codons (independently of their synonymous) better reflects their importance and should be analyzed and plotted.

Referee #3 (Remarks for Author):

Authors answered the all concerns by Reviewers.

Point-by-point response

Manuscript number: EMM-2021-15608-V2

Corresponding author(s): Sultan Cingöz, Brandon J. Wainwright, and Sebastian Glatt

Referee #1 (Remarks for Author):

The authors properly addressed all my comments. Therefore, this revised version is suitable for publication.

Referee #2 (Remarks for Author):

I would like to thank the authors for the extensive revision of their paper. I found the paper suitable for publication as it is.

I would like to share one last comment: in Appendix figure S2B, authors analyzed codon bias (RSCU) in mRNA encoding proteins UP or down regulated in the mutants. I personally find that rather than the bias, the actual frequency of individual codons (independently of their synonymous) better reflects their importance and should be analyzed and plotted.

Response: We agree with the reviewer and have re-analyzed the codon bias of our proteomics dataset. We now plot the codon usage (Δ Freq) in Appendix Figure S2B and updated the respective figure in the Appendix section.

Referee #3 (Remarks for Author):

Authors answered the all concerns by Reviewers.

In addition to the requested changes, we have added unmasked FSC curves for both cryo-EM structures to Appendix Figure 1B. During the EMDB deposition process, we learned that the resolution limits of the EMDB entries are exclusively calculated without a mask. In our case this leads to substantial differences in the reported and calculated resolution ranges. We have uploaded and associated the used masks and FSC curves obtained from CryoSPARC to the EMDB entries as well. Therefore, we would prefer to report the updated masked (4.32 Å for human Elp456 and 4.03 Å for mouse Elp456) and unmasked resolution limits for both reconstructions, namely human and mouse Elp456 in our manuscript. The entry IDs, the actual maps and experimental results are of course not affected by these changes. We have submitted the final validation reports of the accepted entries with the submission. The entries are fully validated and ready for release upon acceptance of our publication.

Accepted **23rd May 2022**

We are pleased to inform you that your manuscript is accepted for publication and is now being sent to our publisher to be included in the next available issue of EMBO Molecular Medicine.

EMBO Press Author Checklist

Corresponding Author Name: Sultan Cingöz, Brandon J. Wainwright and Sebastian Glatt	
Journal Submitted to: EMBO Molecular Medicine	
Manuscript Number: EMM-2021-15608-V2	

USEFUL LINKS FOR COMPLETING THIS FORM
The EMBO Journal - Author Guidelines
EMBO Reports - Author Guidelines
Molecular Systems Biology - Author Guidelines
EMBO Molecular Medicine - Author Guidelines

Reporting Checklist for Life Science Articles (updated January 2022)
This checklist is adapted from Materials Design Analysis Reporting (MDAR) Checklist for Authors. MDAR establishes a minimum set of requirements in transparent reporting in the life sciences (see Statement of Task: 10.31222/osf.io/9sm4x). Please follow the journal's guidelines in preparing your manuscript.
Please note that a copy of this checklist will be published alongside your article.

Abridged guidelines for figures
1. Data
The data shown in figures should satisfy the following conditions:
- the data were obtained and processed according to the field's best practice and are presented to reflect the results of the experiments in an accurate and unbiased manner.
- ideally, figure panels should include only measurements that are directly comparable to each other and obtained with the same assay.
- plots include clearly labeled error bars for independent experiments and sample sizes. Unless justified, error bars should not be shown for technical replicates.
- if n<5, the individual data points from each experiment should be plotted. Any statistical test employed should be justified.
- Source Data should be included to report the data underlying figures according to the guidelines set out in the authorship guidelines on Data Presentation.

2. Captions
Each figure caption should contain the following information, for each panel where they are relevant:
- a specification of the experimental system investigated (eg cell line, species name).
- the assay(s) and method(s) used to carry out the reported observations and measurements.
- an explicit mention of the biological and chemical entity(ies) that are being measured.
- an explicit mention of the biological and chemical entity(ies) that are altered/varied/perturbed in a controlled manner.
- the exact sample size (n) for each experimental group/condition, given as a number, not a range;
- a description of the sample collection allowing the reader to understand whether the samples represent technical or biological replicates (including how many animals, litters, cultures, etc.).
- a statement of how many times the experiment shown was independently replicated in the laboratory.
- definitions of statistical methods and measures:
  - common tests, such as t-test (please specify whether paired vs. unpaired), simple χ2 tests, Wilcoxon and Mann-Whitney tests, can be unambiguously identified by name only, but more complex techniques should be described in the methods section;
  - are tests one-sided or two-sided?
  - are there adjustments for multiple comparisons?
  - exact statistical test results, e.g., P values = x but not P values < x;
  - definition of 'center values' as median or average;
  - definition of error bars as s.d. or s.e.m.

Please complete ALL of the questions below.
Select "Not Applicable" only when the requested information is not relevant for your study.

Materials

Newly Created Materials	Information included in the manuscript?	In which section is the information available? (Reagents and Tools Table, Materials and Methods, Figures, Data Availability Section)
New materials and reagents need to be available; do any restrictions apply?	Yes	Materials and Methods

Antibodies	Information included in the manuscript?	In which section is the information available? (Reagents and Tools Table, Materials and Methods, Figures, Data Availability Section)
For **antibodies** provide the following information:<br>- Commercial antibodies: RRID (if possible) or supplier name, catalogue number and or/clone number<br>- Non-commercial: RRID or citation	Yes	Materials and Methods

DNA and RNA sequences	Information included in the manuscript?	In which section is the information available? (Reagents and Tools Table, Materials and Methods, Figures, Data Availability Section)
**Short novel DNA or RNA including primers, probes**: provide the sequences.	Not Applicable	

Cell materials	Information included in the manuscript?	In which section is the information available? (Reagents and Tools Table, Materials and Methods, Figures, Data Availability Section)
**Cell lines:** Provide species information, strain. Provide accession number in repository **OR** supplier name, catalog number, clone number, and/**OR** RRID.	Yes	Materials and Methods
**Primary cultures:** Provide species, strain, sex of origin, genetic modification status.	Yes	Materials and Methods
Report if the cell lines were recently **authenticated** (e.g., by STR profiling) and tested for mycoplasma contamination.	Not Applicable	

Experimental animals	Information included in the manuscript?	In which section is the information available? (Reagents and Tools Table, Materials and Methods, Figures, Data Availability Section)
**Laboratory animals or Model organisms:** Provide species, strain, sex, age, genetic modification status. Provide accession number in repository **OR** supplier name, catalog number, clone number, **OR** RRID.	Yes	Materials and Methods
**Animal observed in or captured from the field:** Provide species, sex, and age where possible.	Not Applicable	
Please detail **housing and husbandry conditions**.	Yes	Materials and Methods

Plants and microbes	Information included in the manuscript?	In which section is the information available? (Reagents and Tools Table, Materials and Methods, Figures, Data Availability Section)
**Plants:** provide species and strain, ecotype and cultivar where relevant, unique accession number if available, and source (including location for collected wild specimens).	Not Applicable	
**Microbes:** provide species and strain, unique accession number if available, and source.	Not Applicable	

Human research participants	Information included in the manuscript?	In which section is the information available? (Reagents and Tools Table, Materials and Methods, Figures, Data Availability Section)
If collected and within the bounds of privacy constraints report on age, sex and gender or ethnicity for all study participants.	Yes	Table 1

Core facilities	Information included in the manuscript?	In which section is the information available? (Reagents and Tools Table, Materials and Methods, Figures, Data Availability Section)
If your work benefited from core facilities, was their service mentioned in the acknowledgments section?	Yes	Acknowlegement

Design

Study protocol	Information included in the manuscript?	In which section is the information available? (Reagents and Tools Table, Materials and Methods, Figures, Data Availability Section)
If study protocol has been **pre-registered, provide DOI in the manuscript**. For clinical trials, provide the trial registration number **OR** cite DOI.	Not Applicable	
Report the **clinical trial registration number** (at ClinicalTrials.gov or equivalent), where applicable.	Not Applicable	

Laboratory protocol	Information included in the manuscript?	In which section is the information available? (Reagents and Tools Table, Materials and Methods, Figures, Data Availability Section)
Provide DOI OR other citation details if **external detailed step-by-step protocols** are available.	Not Applicable	

Experimental study design and statistics	Information included in the manuscript?	In which section is the information available? (Reagents and Tools Table, Materials and Methods, Figures, Data Availability Section)

Include a statement about **sample size** estimate even if no statistical methods were used.	Yes	Figure legends and Material and Methods
Were any steps taken to minimize the effects of subjective bias when allocating animals/samples to treatment (e.g. **randomization procedure**)? If yes, have they been described?	Yes	Material and Methods
Include a statement about **blinding** even if no blinding was done.	Not Applicable	
Describe **inclusion/exclusion criteria** if samples or animals were excluded from the analysis. Were the criteria pre-established?<br><br>If sample or data points were omitted from analysis, report if this was due to attrition or intentional exclusion and provide justification.	Not Applicable	
For every figure, are **statistical tests** justified as appropriate? Do the data meet the assumptions of the tests (e.g., normal distribution)? Describe any methods used to assess it. Is there an estimate of variation within each group of data? Is the variance similar between the groups that are being statistically compared?	Yes	Figure legends and Material and Methods

Sample definition and in-laboratory replication	Information included in the manuscript?	In which section is the information available?<br>(Reagents and Tools Table, Materials and Methods, Figures, Data Availability Section)
In the figure legends: state number of times the experiment was **replicated** in laboratory.	Yes	Figure legends
In the figure legends: define whether data describe **technical or biological replicates**.	Yes	Figure legends

Ethics

Ethics	Information included in the manuscript?	In which section is the information available?<br>(Reagents and Tools Table, Materials and Methods, Figures, Data Availability Section)
Studies involving **human participants**: State details of **authority granting ethics approval** (IRB or equivalent committee(s), provide reference number for approval.	Yes	Material and Methods
Studies involving **human participants**: Include a statement confirming that **informed consent** was obtained from all subjects and that the experiments conformed to the principles set out in the WMA Declaration of Helsinki and the Department of Health and Human Services Belmont Report.	Yes	Material and Methods (approval of ethics boards)
Studies involving **human participants:** For publication of **patient photos**, include a statement confirming that consent to publish was obtained.	Yes	Material and Methods (approval of ethics boards)
Studies involving experimental **animals**: State details of **authority granting ethics approval** (IRB or equivalent committee(s), provide reference number for approval. Include a statement of compliance with ethical regulations.	Yes	Material and Methods
Studies involving **specimen and field samples:** State if relevant **permits** obtained, provide details of authority approving study; if none were required, explain why.	Not Applicable	

Dual Use Research of Concern (DURC)	Information included in the manuscript?	In which section is the information available?<br>(Reagents and Tools Table, Materials and Methods, Figures, Data Availability Section)
Could your study fall under dual use research restrictions? Please check biosecurity documents and list of **select agents and toxins** (CDC): https://www.selectagents.gov/sat/list.htm	Not Applicable	
If you used a select agent, is the security level of the lab appropriate and reported in the manuscript?	Not Applicable	
If a study is subject to dual use research of concern regulations, is the name of the **authority granting approval and reference number** for the regulatory approval provided in the manuscript?	Not Applicable	

Reporting

The MDAR framework recommends adoption of discipline-specific guidelines, established and endorsed through community initiatives. Journals have their own policy about requiring specific guidelines and recommendations to complement MDAR.

Adherence to community standards	Information included in the manuscript?	In which section is the information available?<br>(Reagents and Tools Table, Materials and Methods, Figures, Data Availability Section)
State if relevant guidelines or checklists (e.g., **ICMJE, MIBBI, ARRIVE, PRISMA**) have been followed or provided.	Not Applicable	
For **tumor marker prognostic studies,** we recommend that you follow the **REMARK** reporting guidelines (see link list at top right). See author guidelines, under 'Reporting Guidelines'. Please confirm you have followed these guidelines.	Not Applicable	
For **phase II and III randomized controlled trials**, please refer to the **CONSORT** flow diagram (see link list at top right) and submit the CONSORT checklist (see link list at top right) with your submission. See author guidelines, under 'Reporting Guidelines'. Please confirm you have submitted this list.	Not Applicable	

Data Availability

Data availability	Information included in the manuscript?	In which section is the information available?<br>(Reagents and Tools Table, Materials and Methods, Figures, Data Availability Section)
Have **primary datasets** been deposited according to the journal's guidelines (see 'Data Deposition' section) and the respective accession numbers provided in the Data Availability Section?	Yes	Data Availability Section
Were **human clinical and genomic datasets** deposited in a public access-controlled repository in accordance to ethical obligations to the patients and to the applicable consent agreement?	Yes	Data Availability Section
Are **computational models** that are central and integral to a study available without restrictions in a machine-readable form? Were the relevant accession numbers or links provided?	Not Applicable	
If publicly available data were reused, provide the respective **data citations in the reference list**.	Not Applicable	
